# Photoredox-Catalyzed Giese Reactions: Decarboxylative Additions to Cyclic Vinylogous Amides and Esters

**DOI:** 10.3390/molecules27020417

**Published:** 2022-01-10

**Authors:** Kevin Dykstra, Alexei Buevich, Qi Gao, Yu-Hong Lam, Jeffrey T. Kuethe

**Affiliations:** 1 Department of Medicinal Chemistry, Merck & Co., Inc., 2000 Galloping Hill Road, Kenilworth, NJ 07033, USA; Kevin_Dykstra@merck.com; 2 Analytical Research and Development, Merck & Co., Inc., 2000 Galloping Hill Road, Kenilworth, NJ 07033, USA; Alexei.Buevich@merck.com (A.B.); Qi.Gao1@merck.com (Q.G.); 3 Department of Computational and Structural Chemistry, Merck & Co., Inc., Rahway, NJ 07065, USA; Yu.Hong.Lam@merck.com; 4 Department of Process Research and Development, Merck & Co., Inc. Rahway, NJ 07065, USA

**Keywords:** photoredox, dihydropyridones, vinylogous amides and esters

## Abstract

An effective strategy has been developed for the photoredox-catalyzed decarboxylative addition of cyclic amino acids to both vinylogous amides and esters leading to uniquely substituted heterocycles. The additions take place exclusively *trans* to the substituent present on the dihydropyridone ring affording stereochemical control about the new carbon-carbon bond. These reactions are operationally simplistic and afford the desired products in good to excellent isolated yields.

## 1. Introduction

**Figure 1:**
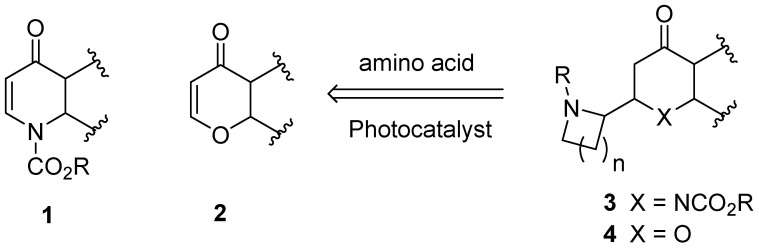
Decarboxylative additions to cyclic vinylogous amides and esters.

Reductive addition of carbon-centered radicals to electron-deficient olefins, known as the Giese reaction, has been utilized in a number of synthetic applications including total syntheses [1,2,3,4,5,6,7]. Visible-light photoredox-catalyzed Giese reactions have garnered a great deal of interest in recent years as a valuable method for the construction of carbon-carbon bonds in an atom-economical manner under relatively mild reaction conditions. Recent examples of carbon-centered radicals utilized in the Giese reaction have been generated via carboxylic acids [8,9,10], trifluoroborate salts [11,12], secondary and tertiary alcohols [13,14,15], organosilicates [16,17,18], alkyl halides [19], and via triplet enone diradicals [20]. Decarboxylative Giese reactions involving readily available amino acids have emerged as a powerful method for the construction of carbon-carbon bonds leading to the formation of molecules not previously accessible by other methods [8,9]. Reactions leading to increasing molecular complexity are extremely valuable synthetic tools and our interest in this area was inspired by the unique reactivity of cyclic vinylogous amides and esters of type **3** and **4** (Figure 1). It was envisioned that addition of carbon-centered radicals generated via a photocatalytic decarboxylation of amino acids would allow for the preparation of unprecedented and novel heterocyclic scaffolds **3** and **4**. In this manuscript we document our investigations in this area.

## 2. Results and Discussion

**Scheme 1:**
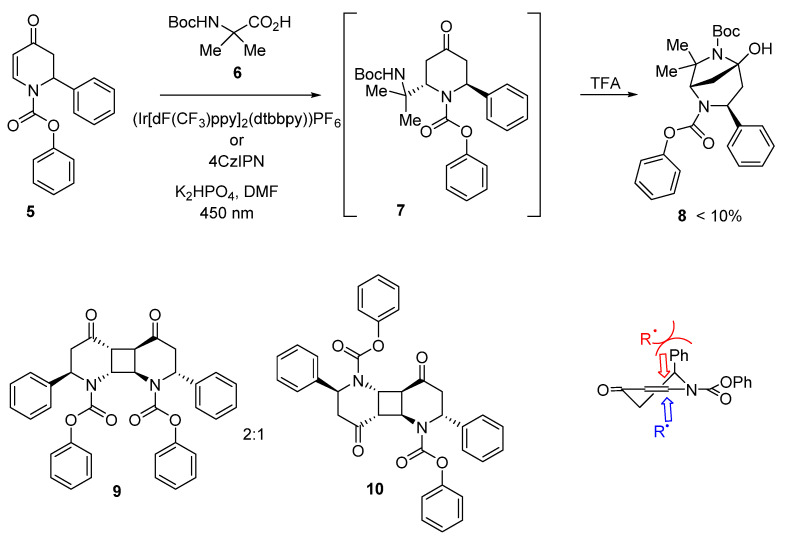
Initial screening and observations.

Our investigations began by examining the reaction between dihydropyridone **5** [21] and amino acid **6** (Scheme 1). A high throughput screen of 24 photocatalysts was conducted employing potassium phosphate dibasic or potassium carbonate as bases and dimtheylformamide (DMF) or dimethylsulfoxide (DMSO) as solvents. The starting materials were dosed into pre-assembled vials containing a separate photocatalyst and base and were irradiated at 450 nm for 24 h. The samples were then analyzed by liquid chromatography mass spectrometry (LCMS). The first screen yielded two “hits” for the desired mass. The photocatalysts of interest were identified as (Ir[dF(CF_3_)ppy]_2_(dtbby))PF_6_ [22] and 1,2,3,5-tetrakis(carbazol-9-yl)-4,6-dicyanobenzene (4CzIPN) [23] employing potassium phosphate dibasic and DMF. These reactions were subsequently scaled up to 100 mg scale and purified by mass-directed HPLC. Interestingly, upon examining the nuclear magnetic resonance (NMR) of the isolated product it was found that the radical added exclusively *trans* to the phenyl group on the dihydropyridone. We speculate that the initially formed product **7** undergoes cyclization to aminal **8** during the mass-directed purification which employed aqueous trifluoroacetic acid (TFA) and acetonitrile. The observation that the radical approached *trans* to the phenyl group was further supported by molecular calculations which demonstrated that a *cis* approach is 3.8 kcal/mol higher in energy than the *trans* approach. This is perfectly in line with the fact that substituents on dihydropyridines bearing a carbamate prefer an axial orientation due to A^1,3^ strain between an equatorial substituent and the carbamate. This is also in line with the fact that Grignard reagents favor the trans products when reacted with dihydropyridones of type **5**. The resulting steric environment of the axial substituent leads to the radical approaching from the opposite face leading to the observed *cis*-isomer. Although the desired product was formed in the reaction, the isolated yield was <10% for the iridium photocatalyst and < 5% for the 4CzIPN catalyst. Reexamination of the crude reaction mixture revealed that at least two other major products had formed but were inseparable from one another. After a series of NMR experiments on the crude reaction mixture the structures were determined to be a 2:1 mixture of 2 + 2 dimers **9** and **10**. It is believed that the initially formed radical generated from amino acid **6** is sufficiently stable or self-quenches and dimerization becomes the major pathway. We speculate that intermolecular photocycloaddition leading to compounds **9** and **10** occurs through energy transfer from the iridium catalyst upon light absorption. The reaction was repeated with several other primary, secondary and tertiary acyclic amino acids and similar results were obtained.

**Scheme 2:**
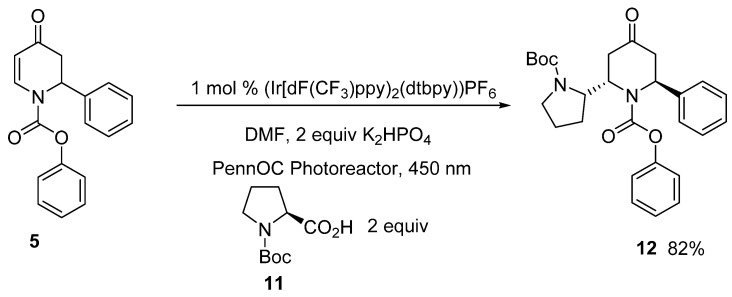
Photocatalytic decoarboxyative addition of cyclic amino acids.

Our attention then turned to screening cyclic amino acids in order to probe whether these would have better reactivity and deliver the desired product in useful yields. The initial photocatalytic screen was performed employing dihydropyridone **5** and Boc L-proline **11** (Scheme 2). The same optimal conditions discovered above were found also in this screen and the desired product was formed in much higher apparent yield. The optimal conditions employed were to irradiate at 450 nm the dihydropyridone **5** in DMF in the presence of 1 mol% Ir[dF(CF_3_)ppy]_2_(dtbby) and 2 equiv of both K_2_HPO_4_ and Boc L-proline for 20 h. Under these conditions, the desired product **12** was obtained in 82% isolated yield and as a 1:1 inseparable mixture of diastereomers about the carbon where the radical was formed. There were only trace amounts of 2 + 2 dimers in the crude NMR and these were easily separable from the products In addition, the *trans* product was the exclusive product formed, there being no detectable amounts of the *cis* products formed in the reaction. The *trans* stereochemistry was further confirmed by NMR.

**Scheme 3:**
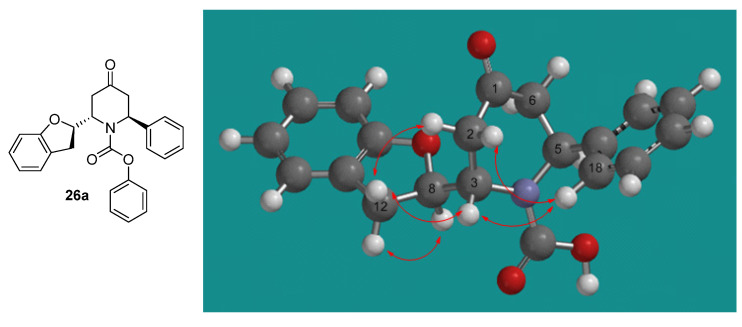
Confirmed stereochemistry of major diastereomer of compound **26**.

With these results in hand, the scope of the transformation was explored employing the optimized conditions. For example, an azetidine radical generated from Boc-protected amino acid **13** cleanly added to dihydropyridone **5** to give product **14** in 90% isolated yield and as an inseparable mixture of diastereomers. (Table 1, entry 1). Interestingly, reaction with amino acid **15** provided the desired product with modest levels of diastereoselectivity (2.8:1) where the major product of **16** could be isolated by fractional crystallization of the mixture from EtOAc/hexane after purification by silica gel chromatography (Table 1, entry 2). In similar fashion, reaction of amino acid **17** with dihydropyridone **5** also provided a 2.8:1 mixture of diastereomeric products **18**; however, these products could not be separated from one another by either silica gel or fractional crystallization (Table 1, entry 3). The morpholine and indoline amino acids **19** and **21** also added to dihydropyridone **5** providing the desired products **20** and **22** in 85% and 69% yields, respectively (Table 1, entries 4,5). In both cases, there were no observable levels of diastereoselectivety and a 1:1 mixture of products was obtained. The diastereomers of **20** were separable by silica gel chromatography; however, the diastereomers of **22** were inseparable. As an extension of these investigations, it was discovered that tetrahydropyran-2-carboxylic acid **23** could also be utilized leading to compound **24** in 58% yield and a separable 2:1 mixture of diastereomers (Table 1, entry 6). In addition, dihydrobenzofuran-2-carboxylic acid **25** underwent smooth decarboxylative radical formation and addition to dihydropyridone **5** to provide product **26** in 82% isolated yields and as a 3:1 separable mixture of diastereomers (Table 1, entry 7). The major diastereomer of compound **26** was subjected to a series of NMR experiments and density functional theory (DFT) calculations in order to determine the configuration of the 3 chiral centers of the major diastereomer. From these experiments it was determined that the major diastereomer 26a had the stereochemistry as depicted in Scheme 3.

**Scheme 4:**
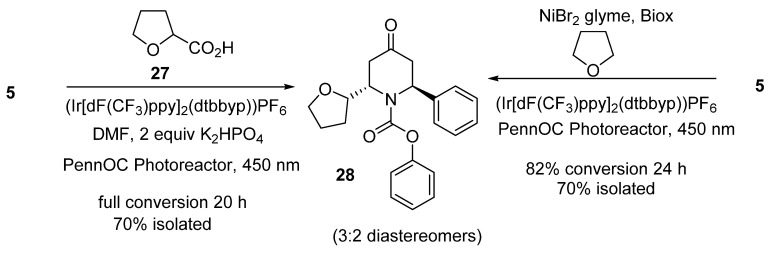
Complementary additions of THF to dihydropyridone **5**.

Reaction of tetrahydrofuran-2-carboxylic acid **27** with dihyropyridone **5** under the standard conditions (Ir[dF(CF_3_)ppy]_2_(dtbby))PF_6_ 1 mol%, 450 nm, K_2_HPO_4_ 2 equiv, 20 h) led to full conversion and afforded the expected product **28** as a separable 3:2 mixture of diasteremers and was isolated in 70% yield (Scheme 4). A complementary approach to this molecule involved the recently reported nickel-catalyzed addition of THF to enones involving an energy-transfer initiated catalysis involving triplet diradicals [20]. Irradiation at 450 nm of dihydropyridone **5** in the presence of 1 mol% (Ir[dF(CF_3_)ppy]_2_(dtbby))PF_6_ in the presence of 5 mol% NiBr_2_ glyme, 15 mol% 2,2′-bis(2-oxazoline (BiOx) in tetrahydrofuran (THF) resulted in 82% conversion after 24 h and afforded compound **28** in 70% isolated yield. Under these conditions, compound **28** was obtained as a 3:2 mixture of diastereomers which was identical to the decarboxylative process and was obtained in similar levels of diastereoselectivity.

**Scheme 5:**
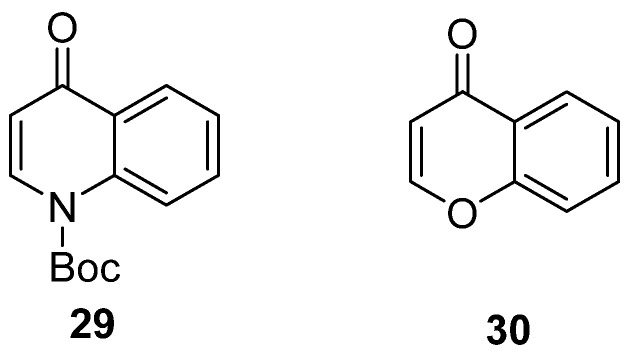
4-oxoquinoline **29** and chromen-4-one **30**.

We next turned our attention to the addition of these carbon-centered radicals to both 4-oxoquinolines **29** [24] and chromen-4-ones **30** to further broaden the scope of the transformation (Scheme 5). As revealed in Table 2, reaction with amino acids **13** and **11** with 4-oxoquinoline **29** under the standard conditions provided products **31** and **32** as a 1:1 mixture of diastereomers in 38% and 55% isolated yields, respectively. The individual diastereomers of product **31** were separable whereas the diastereomers of product **32** could not be separated from one another. Reaction of compound **29** with amino acids **14** and **16** provided the desired products **33** and **34** in slightly higher yield. In each of these cases the observed diastereoselectivity increased to 4:1; however, these diastereomers were inseparable from one another. Reaction of chromenone **30** with the radicals generated from amino acids **11** and **21**, was also successful providing the addition products **35** and **36** in good yields and as inseparable mixtures of diastereomers. In addition, the radical generated from tetrahydrofuran-2-carboxylic acid gave product **37** in 68% isolated yield where the individual diastereomers could be separated from each other. Finally, reaction of chromenone **30** with amino acid **15** afforded heterocycle **38** in 92% yield and as a 1:1 mixture of separable diastereomers.

## 3. Materials and Methods

All anhydrous solvents were supplied by Sigma Aldrich in Sureseal^®^ bottles and used without further purification. All commercially available chemicals were used as received. Reactions were monitored by ultra-performance liquid chromatography UPLC employing an Agilent Technologies 1290 Infinity II UPLC with a Waters Aquity UPLC DEH C18 column (1.7 mm, 2.1 × 100 mm, 0.4 mL/min, 40 ℃ solvent A 0.1% H_3_PO_4_/water: B MeCN, 90:10 to 10:90 A:B over 8 min). Silica gel chromatography was performed with a 24-gram pre-packaged cartridge on a Teledyne ISCO CombiFlash Rf using a gradient of 0–100% methyl tert-butyl ether (MTBE)/hexane. NMR spectra were obtained on a Bruker 500 MHz spectrometer. Elemental analysis was performed at Intertek Pharmaceutical Services. HMRS were obtained at Merck & Co., Inc., Kenilworth, NJ, USA.

*Preparation of Trans-phenyl-1-(tert-butoxycarbonyl)pyrrolidin-2-yl)-4-oxo-6-phenylpiperidine-1-carboxylate* (**12**). According the the general procedure, reaction of 200 mg (10.68 mmol) of dihydropyridone **5** with 294 mg (1.36 mmol) of *N*-Boc-L-proline **11** in the presence of 238 mg (1.36 mmol) of HK_2_PO_4_ and 10 mg (0.012 mmol) of (Ir[dF(CF_3_)ppy]2(dtbpy))PF_6_ provided 260 mg (82%) of compound **12** as a colorless oil and as inseparable 1:1 mixture of diastereomers and rotamers: ^1^H NMR (500 MHz, CDCl_3_) δ 7.36 (m, 2H), 7.30–7.12 (m, 7H), 6.74 (d, 2H, *J* = 7.9 Hz), 5.73 and 5.61 (d, 1H, *J* = 6.4 and 6.6 Hz), 4.65–4.47 (m, 1.17H), 4.39–4.29 (m, 0.64H), 3.97 (m, 0.66H), 3.60–3.49 (m, 0.80H), 3.47–3.31 (m, 1H), 3.20 (br m, 0.30H), 2.85 (m, 1.3 H), 2.69–2.54 (m, 1.27H), 2.23 (m, 0.57H), 2.05–1.87 (m, 2.6H), 1.68 (m, 0.62H), 1.63–1.45 (m, 9H); ^13^C-NMR (CDCl_3_, 125 MHz) δ 206.6, 155.6, 155.2, 151.1, 143.5, 129.3, 129.1, 128.9, 128.8, 127.4, 127.2, 127.0, 125.5, 125.3, 125.2, 125.1, 121.8, 121.6, 121.5, 80.1, 79.5, 60.9, 56.5, 56.2, 55.5, 54.5, 53.7, 47.4, 47.2, 46.1, 46.0, 44.8, 41.0, 40.9, 39.7, 29.1, 29.0, 28.6, 28.4, 27.0, 23.3, 23.0. HRMS Calcd. For C_27_H_33_N_2_O_5_ [M = H}: 465.2389. Found: 465.2380.

*Preparation of Trans-1-(tert-butoxycarbonyl)azetidin-2-yl)-4-oxo-6-phenylpiperidine-1-carboxylate* (**14**). According the the general procedure, reaction of 160 mg (0.55 mmol) of dihydropyridone **5** with 220 mg (1.10 mmol) *N*-Boc-azetidine-2-carboxylic acid **13** in the presence of 190 mg (1.13 mmol) of HK_2_PO_4_ and 9.4 mg (8.4 mmol) of (Ir[dF(CF_3_)ppy]2(dtbpy))PF_6_ provided 220 mg (90%) of compound **14** as colorless oil and as an inseparable 1:1 mixture of diastereomers and rotamers: ^1^H NMR (500 MHz, CDCl_3_) δ 7.42–7.27 (m, 7H), 7.19 (br m, 2H), 6.95 (br m, 1H), 5.78 (m, 1H), 5.04 (m, 0.5H), 4.93 (m, 0.5H), 4.60 (m, 0.5H), 4.38 (m, 0.5H), 3.91 (m, 1.5H), 3.75 (dt, 1.5H, *J* = 9.0 and 6.7 Hz), 3.42 (m, 0.5H), 2.99–2.81 (m, 2H), 2.72 (m, 0.5H), 2.54 (m, 1.5H), 1.94 (m, 1H), 1.51 and 1.49 (s, 9H); ^13^C-NMR (CDCl_3_, 125 MHz) δ 205.8 and 205.1, 157.8, 151.0, 142.1, 129.4, 129.3, 129.2, 129.0, 128.9, 127.4, 127.3, 125.6, 125.4, 125.3, 125.2, 121.6, 80.4, 64.9, 64.4, 56.4, 55.2, 54.5, 47.3, 45.8, 44.2, 39.4, 37.7, 28.5 and 28.4, 21.3, 20.0. HRMS Cacld. For C_26_H_31_N_2_O_5_ [M + H]: 451.2233. Found: 451.2239.

*Preparation of Trans-1-(tert-butyl-1-phenyl-4-oxo-6-phenyl-[2,2’-bipiperidine]-1,1’dicarboxylate* (**16**). According the the general procedure, reaction of 133 mg (0.45 mmol) of dihydropyridone **5** with 158 mg (0.91 mmol) *N*-Boc-pipecolic acid **15** in the presence of 208 mg (0.91 mmol) of HK_2_PO_4_ and 7.5 mg (6.7 mmol) of (Ir[dF(CF_3_)ppy]2(dtbpy))PF_6_ provided 183 mg (83%) of compound **16** as separable 2.8:1 mixture of diastereomers and rotamers: First Isomer to elute: colorless oil; ^1^H NMR (500 MHz, CDCl_3_) δ 7.39 (m, 2H), 7.24 (m, 5H), 7.13 (m, 1H), 6.62 (br m, 2H), 6.66 and 5.52 (br m, 1H), 5.18 and 5.07 (br m, 1H), 4.42 (br m, 1H), 4.16 (br m, 1H), 3.99 (d, 1H, *J* = 12.4 Hz), 3.55–3.32 (br m, 1H), 2.89–2.55 (m, 2H), 1.74–1.48 (m, 15H);); ^13^C-NMR (CDCl_3_, 125 MHz) δ 206.2, 158.9, 129.1, 129.0, 128.9, 127.4, 127.2, 125.5, 125.4, 125.0, 121.5, 121.4, 56.8, 53.6, 49.9, 45.5, 41.3, 40.7, 28.5, 28.1, 25.6, 24.8, 18.9. HRMS calcd for C_28_H_34_N_2_O_5_ [M + H]: 479.2546. Found: 479.2552. Second Isomer to elute: colorless solid; ^1^H NMR (500 MHz, CDCl_3_) δ 7.50 (m, 2H), 7.25 (m, 5H), 7.16 (m, 1H), 6.74 (br m, 2H), 5.66 (m, 1H), 5.11 (m, 1H), 4.70 (br m, 0.5H), 4.50 (br m, 0.5H), 4.21 (d, 0.5H, *J* = 12.7 Hz), 4.01 (d, 0.5H, *J* = 12.4 Hz), 3.69 (m, 0.5H), 3.43 (m, 0.5H), 2.89–2.54 (m, 4H), 1.94 (br m, 2H), 1.72–1.53 (m, 3H), 1.46 (s, 9H); ^13^C-NMR (CDCl_3_, 125 MHz) δ 204.3, 155.3, 145.3, 150.8, 129.3, 129.2, 129.0, 128.9, 127.6, 127.4, 125.7, 125.6, 125.0, 121.4, 80.9, 80.2, 56.4, 56.3, 54.8, 53.2, 49.8, 49.4, 45.4, 45.2, 40.6, 39.7, 39.6, 39.0, 28.5, 28.4, 28.2, 25.7, 25.3, 25.0, 24.7, 19.4, 19.3, 18.9. Anal. Calcd. For C_28_H_34_N_2_O_5_: C, 70.27; H, 7.16; N, 5.85. Found, C, 69.97; H, 6.99; N, 5.82.

*Preparation of Trans-tert-butyl-4-oxo-1-(phenoxycarbonyl)-6-phenylpiperidin-2-yl)-3,4-dihydroquinoline-1(2H)-carboxylate* (**18**). According the the general procedure, reaction of 164 mg (0.56 mmol) of dihydropyridone **5** with 310 mg (1.12 mmol) of *N*-Boc-tetrahydroquinoline-2-carboxylic acid **17** in the presence of 195 mg (1.12 mmol) of HK_2_PO_4_ and 13 mg (0.011 mmol) of (Ir[dF(CF_3_)ppy]2(dtbpy))PF_6_ provided 202 mg (68%) of compound **18** as colorless oil and an inseparable 2.8:1 mixture of diastereomers and rotamers: ^1^H NMR (500 MHz, CDCl_3_) δ 7.45–7.40 (m, 12H), 6.75 and 6.88 (br m, 2H), 5.79 and 5.73 (d, 1H, *J* = 5.6 and 6.9 Hz), 5.11 and 4.84 (m, 1H), 4.64–4.57 (br m, 1H), 4.03 and 3.57 (m, 1H), 3.01–2.89 (m, 2H), 2.57–2.38 (m, 3H), 2.00 (m, 1H), 1.54 and 1.51 (s, 9H); ^13^C-NMR (CDCl_3_, 125 MHz) δ 206.1 and 204.6, 155.1 and 154.7, 151.2 and 150.9, 143.8 and 142.2, 137.1 and 136.3, 131.6, 129.4, 129.1, 128.9, 128.8, 128.4 and 128.3, 127.5 and 127.3, 126.2, 126.1, 125.9, 125.7, 125.2, 125.1, 124.8, 124.7, 121.6 and 121.4, 81.7 and 81.1, 57.0, 56.5, 55.5, 54.9, 45.7 and 45.0, 41.1, 39.3, 30.3, 28.4 and 28.3, 26.9, 26.2, 24.2. HRMS Calcd. For C_32_H_35_N_2_O_5_ [M + H]: 527.2546 Found: 527.2553.

*Preparation of Trans-tert-butyl-4-oxo-1-(phenoxycarbonyl)-6-phenylpiperidin-2-yl)morpholine-4-carboxylate* (**20**). According the the general procedure, reaction of 158 mg (0.539 mmol) of dihydropyridone **5** with 249 mg (1.08 mmol) of 4-Boc-3-morpholinecarboxylic acid **19** in the presence of 188 mg (1.08 mmol) of HK_2_PO_4_ and 9.1 mg (8.1 mmol) of (Ir[dF(CF_3_)ppy]2(dtbpy))PF_6_ provided 310 mg (65%) of compound **20** as a separable 1:1 mixture of diastereomers and rotamers in 85% combined yield. Isomer #1: colorless oil; ^1^H NMR (500 MHz, CDCl_3_) δ 7.45–7.24 (m, 7H), 7.14 (m, 1H), 6.62 (br m, 2H), 5.65 and 5.51 (d, 1H, *J* = 6.8 and 7.2 Hz), 5.40–5.31 (m, 1H), 4.15–3.47 (m, 7H), 3.10–3.02 (m, 1H), 2.93–2.75 (m, 2H), 1.61 and 1.54 (m, 9H), 1.50 (m, 1H); ^13^C-NMR (CDCl_3_, 125 MHz) δ 206.0, 205.0, 155.5, 150.9, 143.9, 129.3, 129.1, 129.0, 128.9, 127.6, 127.3, 125.7, 125.5, 125.1, 125.0, 121.8, 121.5, 80.9, 80.6, 67.5, 66.9, 66.6, 57.0, 56.2, 55.9, 53.9, 48.6, 45.5, 44.9, 41.1, 40.8, 39.3, 28.7, 28.5, 28.3, 28.1. HRMS Calcd. For C_27_H_33_N_2_O_5_ [M + H]: 480.2260. Found: 480.2257. Isomer #2: colorless oil; ^1^H NMR (500 MHz, CDCl_3_) δ 7.39 (m, 2H), 7.28 (m, 5H), 7.16 (m, 1H), 6.87 (br m, 2H), 5.69 (m, 1H), 5.27 (m, 1H), 4.48 (d, 0.5H, *J* = 7.9 Hz), 4.30 (d, 0.5H, *J* = 8.2 Hz), 4.20 (d, 0.5H, *J* = 12.0 Hz), 4.13 (d, 0.5H, *J* = 12.0 Hz), 4.03 (m, 0.5H), 3.90 (m, 1H), 3.81 (d, 0.5H, *J* = 12.5 Hz), 3.64 (m, 1.7H), 3.50 (m, 1.3H), 3.33 (dd, 0.5H, *J* = 16.9 and 6.9 Hz), 3.09 (m, 0.5H), 3.00–2.69 (m, 3H), 2.60 (m, 0.5H), 1.51 (m, 1H), 1.48 (s, 9H). HRMS Calcd. For C_27_H_33_N_2_O_5_ [M + H]: 481.2339. Found: 481.2242.

*Preparation of Trans-tert-butyl-4-oxo-1-(phenoxycarbonyl)-6-phenylpiperidine-2-yl)indoline-1-carboxylate* (**22**). According the the general procedure, reaction of 166 mg (0.56 mmol) of pyridone **5** with 298 mg (1.13 mmol) *N*-Boc-indoline-2-carboxylic acid 21 in the presence of 197 mg (1.13 mmol) of HK_2_PO_4_ and 9.4 mg (8.4 mmol) of (Ir[dF(CF_3_)ppy]2(dtbpy))PF_6_ provided 201 mg (69%) of compound **22** as colorless oil and as an inseparable 1:1 mixture of diastereomers and rotamers: ^1^H NMR (500 MHz, CDCl_3_) δ 7.49–6.70 (m, 15H), 5.91 and 5.82 (d, 1H, *J* = 7.5 and 6.5 Hz), 5.62 (br m, 0.5H), 5.45 (br m, 1H), 4.66 (m, 1H), 4.57 (m, 1H), 3.55 (dd, 1H, *J* = 16.5 and 10.1 Hz), 3.41 (dd, 0.5H, *J* = 16.0 and 8.3 Hz), 3.29 (dd, 1.2H, *J* = 16.8 and 7.4H), 2.99-2.90 (br m, 2.3H), 2.73 (br dd, 2.25H, *J* = 29.2 and 17.5 Hz), 2.49 (dd, 1.2H, *J* = 17.5 and 7.3 Hz), 2.23 (d, 1H, *J* = 17.8 Hz), 1.65 and 1.63 (s, 9H); ^13^C-NMR (CDCl_3_, 125 MHz) δ 206.4, 203.5, 153.5, 152.5, 151.2, 142.6, 142.5, 130.4, 129.6, 129.5, 129.3, 129.1, 128.9, 128.3, 127.7, 127.4, 125.6, 125.3, 125.1, 124.5, 123.8, 123.2, 121.7, 121.3, 82.0, 62.5, 60.3, 58.6, 57.5, 56.7, 51.7, 46.1, 44.0, 43.1, 40.1, 37.8, 34.8, 32.6, 28.5, 28.4. HRMS Calcd. For C_31_H_33_N_2_O_5_ [M + H]: 513.2389. Found: 513.2379.

*Preparation of Trans-phenyl-4-oxo-2-phenyl-2-tetrahydro-2H-pyran-2-yl)piperidine-1-carboxylate* (**24**). According the the general procedure, reaction of 160 mg (0.55 mmol) of dihydropyridone **5** with 142 mg (1.09 mmol) tetrahydropyran-2-carboxylic acid **23** in the presence of 190 mg (1.09 mmol) of HK_2_PO_4_ and 6.0 mg (8.2 mmol) of (Ir[dF(CF_3_)ppy]2(dtbpy))PF_6_ provided 120 mg (58%) of compound **24** as a separable 2:1 mixture of diastereomers: Major Isomer: colorless oil; ^1^H NMR (500 MHz, CDCl_3_) δ 7.45–7.21 (m, 8.5H), 6.99 (br m, 1.5H), 5.80 (s, 1H), 4.44 (s, 1H), 4.00 (d, 1H, *J* = 10.8 Hz), 3.90 (d, 1H, *J* = 11.5 Hz), 3.61 (m, 1H), 3.44 (m, 1H), 2.95 (dd, 1H, *J* = 17.4 and 1.8 Hz), 2.67 (d, 1H, *J* = 17.4 Hz), 2.49 (br dd, 1H, *J* = 17.4 and 7.1 Hz), 1.89 (m, 1H), 1.66–1.49 (m, 4H), 1.34 (m, 1H); ^13^C-NMR (CDCl_3_, 125 MHz) δ 206.1, 155.1, 151.0, 142.1, 129.3, 128.9, 127.3, 125.6, 125.3, 121.7, 79.4, 68.5, 57.0, 54.4, 45.1, 37.5, 28.3, 25.6, 23.4. HRMS Calcd. For C_23_H_26_NO_4_ [M + H]: 379.1784. Found: 379.1784. Minor Isomer: colorless oil; ^1^H NMR (500 MHz, CDCl_3_) δ 7.38 (t, 2H, *J* = 7.6 Hz), 7.26 (m, 5H), 7.17 (t, 1H, *J* = 7.3 Hz), 6.83 (br m, 2H), 5.74 (d, 1H, *J* = 5.7 Hz), 4.87 (m, 1H), 4.04 (d, 1H, *J* = 8.5 Hz), 3.65 (br m, 1H), 3.50–3.39 (m, 2H), 2.85–2.76 (m, 2H), 2.61 (dd, 1H, *J* = 17.7 and 1.5 Hz), 1.92 (m, 2H), 1.64–1.48 (m, 4H);); ^13^C-NMR (CDCl_3_, 125 MHz) 206.3, 151.0, 145.2, 129.2, 128.9, 127.2, 125.5, 125.2, 121.6, 81.6, 69.1, 55.4, 45.8, 41.3, 28.8, 25.9, 23.4. HRMS Calcd. For C_23_H_26_NO_4_ [M + H]: 380.1862. Found: 380.1849.

*Preparation of Trans-phenyl 2-(2,3-dihydrobenzofuran-2-yl)-4-oxo-6-phenylpiperidine-1-carboxylate* (**26**). According the the general procedure, reaction of 158 mg (0.54 mmol) of dihydropyridone **5** with 177 mg (1.08 mmol) of 2,3-dihydrobenzofuran-2-carboxylic acid **25** in the presence of 188 mg (1.08 mmol) of HK_2_PO_4_ and 9.1 mg (8.1 mmol) of (Ir[dF(CF_3_)ppy]2(dtbpy))PF_6_ provided 310 mg (65%) of compound **26** as a separable 3:1 mixture of diastereomers and rotamers in 82% combined yield. Major isomer: colorless solid; ^1^H NMR (500 MHz, CDCl_3_) δ 7.40 (m, 4H), 7.35 (m, 3H), 7.24 (t, *J* = 7.1 Hz, 1H), 7.16 (dd, *J* = 17.3, 7.7 Hz, 2H), 7.04 (br m, 2H), 6.89 (t, *J* = 7.4 Hz, 1H), 6.74 (d, *J* = 8.0 Hz, 1H), 5.93 (d, *J* = 5.6 Hz, 1H), 5.49 (t, *J* = 8.8 Hz, 1H), 4.70 (d, *J* = 6.9 Hz, 1H), 3.75 (dd, *J* = 17.8, 6.4 Hz, 1H), 3.52 (dd, *J* = 16.3, 10.2 Hz, 1H), 3.09 (dd, *J* = 17.8, 1.9 Hz, 1H), 2.95 (dd, *J* = 16.3, 7.6 Hz, 1H), 2.55 (dd, *J* = 17.6, 7.4 Hz, 1H), 2.41 (d, *J* = 17.6 Hz, 1H). ^13^C-NMR (CDCl_3_, 125 MHz) δ 205.3, 159.0, 150.9, 129.4, 129.3, 129.1, 129.0, 128.6, 128.3, 127.5, 127.4, 125.8, 125.6, 125.3, 125.2, 124.9, 121.6, 121.4, 121.3, 121.2, 109.5, 58.0, 55.4, 45.5, 44.7, 36.1, 33.1. Anal. Calcd. For C_26_H_23_NO_4_: C, 75.53; H, 5.61; N, 3.39. Found: C, 75.39; H, 5.59; N, 3.33. Minor isomer: colorless solid contaminated with some of the major isomer; ^1^H NMR (500 MHz, CDCl_3_) δ 7.40 (m, 4H), 7.35 (m, 3H), 7.24 (t, *J* = 7.1 Hz, 1H), 7.16 (dd, *J* = 17.3, 7.7 Hz, 2H), 7.04 (br m, 2H), 6.94 (d, *J* = 7.4 Hz, 1H), 6.82 (d, *J* = 8.0 Hz, 2H), 5.70 (d, *J* = 6.5 Hz, 1H), 5.11 (t, *J* = 5.1 Hz, 1H), 4.95 (td, *J* = 8.9, 4.8 Hz, 1H), 3.51 (d, *J* = 10.2 Hz, 2H), 3.35 (d, *J* = 8.6 Hz, 2H), 2.79 (d, *J* = 18.0 Hz, 1H); ^13^C-NMR (CDCl_3_, 125 MHz) δ 205.1, 204.4, 159.1, 158.4, 150.9, 150.8, 141.6, 129.7, 129.4, 129.3, 129.1, 129.0, 128.6, 127.5, 127.4, 126.5, 125.8, 125.7, 125.3, 125.2, 125.1, 125.0, 124.9, 121.7, 121.5, 121.3, 121.2, 109.5, 86.0, 84.0, 58.1, 55.4, 54.3, 36.1, 33.1. HRMS Calcd. For C_26_H_24_NO_4_ [M + H]: 414.4810. Found: 414.4799.

*Preparation of Trans-phenyl-4-oxo-2-phenyl-6-tetrahydrofuran-2-yl)piperidine-1-carboxylate* (**28**). According the the general procedure, reaction of 150 mg (0.51 mmol) of dihydropyridone **5** with 119 mg (1.02 mmol) tetrahydrofuran2-carboxylic acid **27** in the presence of 178 mg (1.02 mmol) of HK_2_PO_4_ and 8.6 mg (7.6 mmol) of (Ir[dF(CF_3_)ppy]2(dtbpy))PF_6_ provided 131 mg (70%) of compound **28** as a separable 1:1 mixture of diastereomers: Major isomer: colorless oil; ^1^H NMR (500 MHz, CDCl_3_) δ 7.40–7.26 (m, 7H), 7.21 (m, 1H), 7.00 (br m, 2H), 5.83 (d, 1H, *J* = 5.6 Hz), 4.65 (d, 1H, *J* = 6.5 Hz), 4.57 (t, 1H, *J* = 7.6 Hz), 3.73 (m, 2H), 3.67 (dd, 1H, *J* = 17.8 and 6.5 Hz), 3.00 (dd, 1H, *J* = 17.8 and 2.0 Hz), 2.53 (dd, 1H, *J* = 17.4 and 6.7 Hz), 2.45 (d, 1H, *J* = 16.8 Hz), 2.16 (m, 1H), 1.90 (m, 2H), 1.56 (m, 1H); ^13^C-NMR (CDCl_3_, 125 MHz) δ 205.6, 151.0, 142.0, 129.3, 129.0, 127.3, 125.6, 125.3, 121.7, 80.9, 69.1, 56.6, 44.6, 36.8, 28.8, 25.8. HRMS Calcd. For C_22_H_24_NO_4_ [M + H]: 366.1705. Found: 366.1699. Minor isomer: colorless oil; ^1^H NMR (500 MHz, CDCl_3_) δ 7.40–7.20 (m, 7H), 7.17 (m, 1H), 6.86 (br m, 2H), 5.76 (d, 1H, *J* = 6.2 Hz), 4.81 (t, 1H, *J* = 6.2 Hz), 4.00–3.90 (m, 2H), 3.82 (q, 1H, *J* = 7.7 Hz), 3.48 (m, 1H), 2.87 (m, 2H), 2.60 (d, 1H, *J* = 17.9 Hz), 2.08 (m, 2H), 1.95 (m, 1H), 1.72 (m, 1H); ^13^C-NMR (CDCl_3_, 125 MHz) δ 205.9, 151.1, 142.7, 129.3, 128.9, 127.3, 125.5, 125.2, 121.5, 82.2, 68.1, 55.3, 54.3, 45.6, 41.4, 29.4, 25.9. HRMS Calcd. For C_22_H_24_NO_4_ [M + H]: 366.1705. Found: 366.1712.

*Preparation of Tert-butyl 2-(1-tert-butoxycarbonyl)azetidin-2-yl)-4-oxo-3,4-dihydroquinoline-1(2H)-carboxylate* (**31**). According the the general procedure, reaction of 159 mg (0.65 mmol) of 4-oxoquinolinone **29** with 261 mg (1.23 mmol) of *N*-Boc-azetidine-2-carboxylic acid **13** in the presence of 182 mg (1.23 mmol) of HK_2_PO_4_ and 10.9 mg (9.72 mmol) of (Ir[dF(CF_3_)ppy]2(dtbpy))PF_6_ provided 100 mg (38%) of compound **31** as a separable mixture of diatereomers. Isomer A: ^1^H NMR (500 MHz, CDCl_3_) δ 7.98 (dd, *J* = 7.8, 1.4 Hz, 1H), 7.63 (d, *J* = 8.2 Hz, 1H), 7.54–7.45 (m, 1H), 7.17 (t, *J* = 7.5 Hz, 1H), 5.12–5.01 (m, 1H), 4.26–4.14 (m, 1H), 3.96–3.78 (m, 2H), 3.19 (d, *J* = 18.3 Hz, 1H), 2.98 (dd, *J* = 18.3, 6.2 Hz, 1H), 2.26 (p, *J* = 10.0, 9.6 Hz, 1H), 2.08 (ddt, *J* = 11.3, 8.8, 5.7 Hz, 1H), 1.58 (s, 9H), 1.42 (s, 9H). ^13^C-NMR (CDCl_3_, 125 MHz) δ 192.6, 157.1, 153.1, 141.6, 133.8, 126.8, 125.7, 124.8, 124.0, 61.7, 57.7, 46.9, 20.3. HRMS Calcd. For C_22_H_31_N_2_O_5_: 403.4990. Found: 403.4986. Isomer B: ^1^H NMR (500 MHz, CDCl_3_) δ 7.91 (d, *J* = 7.8 Hz, 1H), 7.78 (d, *J* = 8.3 Hz, 1H), 7.51 (t, *J* = 7.8 Hz, 1H), 7.13 (t, *J* = 7.5 Hz, 1H), 5.09 (t, *J* = 6.3 Hz, 1H), 4.39 (dt, *J* = 8.6, 5.6 Hz, 1H), 3.75–3.47 (m, 2H), 3.12 (dd, *J* = 18.3, 7.4 Hz, 1H), 2.89 (d, *J* = 18.3 Hz, 1H), 2.28 (s, 1H), 2.01 (d, *J* = 22.3 Hz, 1H), 1.57 (s, 9H), 1.36 (s, 9H). ^13^C-NMR (CDCl_3_, 125 MHz) δ 192.7, 156.0, 153.4, 142.7, 133.7, 126.3, 125.0, 123.6, 82.1, 56.1, 46.8, 40.0, 28.4, 28.2, 19.4. HRMS Calcd. For C_22_H_31_N_2_O_5_ [M + H]: 403.4990. Found: 403.4999.

*Preparation of Tert-butyl 2-(1-(tert-butoxycarbonyl)pyrrolidin-2-yl)-4-oxo-3,4-dihydroquinoline-1(2H) Carboxylate* (**32**). According the the general procedure, reaction of 128 mg (0.52 mmol) of 4-oxoquinolinone **29** with 225 mg (1.04 mmol) of *N*-Boc-L-proline 11 in the presence of 182 mg (1.04 mmol) of HK_2_PO_4_ and 8.8 mg (7.83 mmol) of (Ir[dF(CF_3_)ppy]2(dtbpy))PF_6_ provided 120 mg (55%) of compound **32** as a colorless oil and an inseparable 1:1 mixture of diastereomers and rotamers: ^1^H NMR (500 MHz, CDCl_3_) δ 8.05–7.71 (br m, 1H), 7.78, 7.60 and 7.5 (m, 2H), 7.20–7.10 (m, 1H), 4.87 and 4.60 (m, 1H), 4.15–3.93 (m, 1.90H), 3.04 (dd, 0.26H, *J* = 17.8 and 6.9 Hz), 2.94–2.89 (m, 2H), 2.05–1.85 (br m, 3.7H), 1.68 (m, 1H), 1.56 and 1.55 (s, 9H), 1.35 (br m, 9H); ^13^C-NMR (CDCl_3_, 125 MHz) δ 192.8, 192.7, 155.1, 154.6, 153.4, 153.2, 143.7, 141.3, 133.8, 133.5, 132.8, 127.0, 126.3, 126.2, 125.2, 125.1, 124.2, 123.2, 82.4, 81.0, 59.1, 57.8, 57.2, 56.6, 53.9, 46.8, 46.2, 45.9, 40.9, 40.8, 40.2, 39.7, 28.4, 28.3, 28.2, 28.0, 23.8, 23.5, 22.7, 22.3. HRMS Calcd. For C_23_H_33_N_2_O_5_ [M + H]: 417.5260. Found: 417.5251.

*Preparation of Tert-butyl (2-(1-(tert-butoxycarbonyl)piperidin-2-yl)-4-oxo-3,4-dihydroquinoline-1(2H)-carboxylate* (**33**). According the the general procedure, reaction of 164 mg (0.67 mmol) of 4-oxoquinolinone **29** with 310 mg (1.37 mmol) of *N*-Boc-pipecolic acid 14 in the presence of 233 mg (1.37 mmol) of HK_2_PO_4_ and 11 mg (10.0 mmol) of (Ir[dF(CF_3_)ppy]2(dtbpy))PF_6_ provided 160 mg (55%) of compound **33** as a colorless oil and as inseparable 4:1 mixture of diastereomers and rotamers: ^1^H NMR (500 MHz, CDCl_3_) δ 8.03 and 8.97 (d, 1H, *J* = 7.8 Hz), 7.71–7.45 (m, 2H), 7.20 (m, 1H), 5.36 and 5.24 (m, 1H), 4.36 and 4.21 (m, 1H), 4.13 (m, 0.8H), 4.01–3.80 (m, 0.4H), 3.14–3.01 (m, 0.7H), 2.91 (dd, 0.8 H, *J* =18.3 and 6.0 Hz), 2.78–2.57 (m, 1.8H), 1.84–1.64 (m, 5H), 1.51 (s, 10.8H), 1.44–1.35 (m, 2H), 1.33 (s, 6H); ^13^C-NMR (CDCl_3_, 125 MHz) δ 192.9, 192.5, 192.1, 154.4, 152.9, 142.1, 141.1, 140.8, 134.3, 133.8, 133.5, 127.2, 126.9, 126.5, 126.2, 125.9, 125.8, 125.5, 125.2, 124.5, 124.3, 123.8, 82.4, 80.6, 51.5, 50.7, 50.3, 50.1, 48.8, 48.7, 40.7, 40.4, 39.9, 39.4, 38.7, 28.5, 28.3, 27.9, 26.0, 25.2, 25.1, 24.7, 19.2. HRMS Calcd. For C_24_H_35_N_2_O_5_ [M + H]: 431.5530. Found: 431.5528.

*Preparation of Tert-butyl-3-(1-(tert-butoxycarbonyl)-4-oxo-1,2,3,4-tetrahydroquinolin-2-yl)morpholine-4-carboxylate* (**34**). According the (the general procedure, reaction of 106 mg (0.43 mmol) of 4-oxoquinolinone **29** with 200 mg (0.87 mmol) of 4-Boc-3-morpholinecarboxylic acid **16** in the presence of 151 mg (0.87 mmol) of HK_2_PO_4_ and 7.3 mg (6.5 mmol) of (Ir[dF(CF_3_)ppy]2(dtbpy))PF_6_ provided 122 mg (65%) of compound **34** as a colorless foam and as inseparable 4:1 mixture of diastereomers and rotamers: ^1^H NMR (500 MHz, CDCl_3_) δ 8.01 and 8.00 (m, 1H), 7.79–7.60 (m, 1H), 7.54 (m, 1H), 7.20 (m, 1H), 5.57 and 5.50 (m, 1H), 4.10 and 4.02 (m, 1.2H), 3.94–3.74 (m, 3H), 3.57–3.34 (m, 2.5H), 3.15 (dd, 0.5H, *J* = 17.9 and 5.5 Hz), 3.07–2.93 (m, 1.3H), 2.82 (t, 0.3H, *J* = 17.7 and 5.5 Hz), 2.69 and 2.55 (d, 0.51H, *J* = 18.3 Hz), 1.59 (m, 9.5H), 1.46 and 1.41 (s, 4H), 1.36 (s, 5H), 1.10 (s, 1.8 Hz); ^13^C-NMR (CDCl_3_, 125 MHz) δ 192.7, 192.1, 191.6, 154.1, 154.0, 152.9, 152.8, 142.2, 141.2, 140.9, 134.5, 134.0, 133.6, 127.3, 127.1, 126.6, 126.2, 125.7, 125.5, 125.3, 125.1, 124.5, 124.2, 124.0, 123.9, 82.9, 82.7, 82.4, 81.1, 67.4, 67.0, 66.9, 66.7, 66.4, 66.0, 65.8, 51.5, 50.9, 50.2, 49.6, 49.3, 40.6, 40.2, 38.9, 38.7, 28.3, 28.1, 27.9. HRMS Calcd. For C_23_H_33_N_2_O_5_ [M + H]: 433.5250. Found: 433.5243.

*Preparation of Tert-butyl 2-(4-oxochroman-2-yl)pyrrolidine-1-carboxylate* (**35**). According the the general procedure, reaction of 157 mg (1.07 mmol) of 4*H*-chromen-4-one **30** with 462 mg (2.15 mmol) of *N*-Boc-L-proline **11** in the presence of 374 mg (2.15 mmol) of HK_2_PO_4_ and 18 mg (0.016 mmol) of(Ir[dF(CF_3_)ppy]2(dtbpy))PF_6_ provided 310 mg (65%) of compound **35** as a colorless foam and as inseparable 1:1 mixture of diastereomers and rotamers: ^1^H NMR (500 MHz, CDCl_3_) δ 7.90 (m, 1H), 7.49 (m, 1H), 7.00 (br m, 2H), 4.85–4.54 (br m, 1H), 4.27 (br m, 0.5H), 4.05 (br m, 0.5H), 3.69–3.35 (br m, 2H), 2.78 (m, 1H), 2.67 (m, 1H), 2.25 (m, 0.5H), 2.14–1.88 (br m, 3.5H), 1.48 (s, 9H); ^13^C-NMR (CDCl_3_, 125 MHz) δ 192.5, 192.0, 161.5, 156.2, 155.0, 135.8, 126.9, 126.8, 121.2, 120.9, 118.0, 117.8, 80.1, 79.5, 78.9, 78.4, 77.9, 59.8, 58.8, 47.3, 47.1, 46.7, 42.0, 39.9, 39.4, 28.5, 27.7, 26.6, 25.7, 24.5, 24.2, 23.6, 23.3. HRMS Calcd. For C_18_H_24_NO_4_ [M + H]: 317.1627. Found: 317.1624.

*Preparation of Tert-butyl-2-(4-oxochroman-2-yl)indoline-1-carboxylate* (**36**). According the the general procedure, reaction of 157 mg (1.07 mmol) of 4*H*-chromen-4-one **30** with 424 mg (2.15 mmol) of *N*-Boc-indoline-2-carboxylic acid **21** in the presence of 374 mg (2.15 mmol) of HK_2_PO_4_ and 18 mg (0.016 mmol) of Ir[dF(CF_3_)ppy]2(dtbpy))PF_6_ provided 240 mg (61%) of compound **36** as a colorless oil as an inseparable 1:1 mixture of diastereomers and rotamers: ^1^H NMR (500 MHz, CDCl_3_) δ 7.87 (m, 1H), 7.48 (m, 1H), 7.20 (m, 2H), 7.05–6.89 (m, 3H), 4.95 (br m, 1H), 4.74 and 4.70 (br m, 1H), 3.42 (m, 1H), 3.34–3.23 (m, 1H), 2.90 (dd, *J* = 15.0, 10.0 Hz, 0.5 H), 2.72 (dd, *J* = 15.0, 5.0 Hz, 0.5H), 2.62 (dd, *J* = 15.0 and 14.0 Hz, 0.5H), 2.49 (dd, *J* = 15.0, 5.0 Hz, 0.5H), 1.60 (s, 9H). ^13^C-NMR (CDCl_3_, 125 MHz) δ 191.9, 191.5, 161.4, 161.3, 136.0, 135.8, 127.7, 127.4, 127.0, 126.9, 124.7, 124.5, 123.1, 123.0, 121.5, 121.1, 120.9, 118.1, 117.9, 115.9, 115.7, 78.1, 77.1, 61.5, 60.3, 39.9, 37.1, 28.4. HRMS Calcd. For C_22_H_24_NO_4_ [M + H]: 366.4370. Found: 366.4366.

*Preparation of 2-(Tetrahydrofuran-2-yl)chroman-4-one* (**37**). According the the general procedure, reaction of 133 mg (0.91 mmol) of 4*H*-chromen-4-one **30** with 211 mg (1.82 mmol) of tetrahydrofuran2-carboxylic acid **27** in the presence of 317 mg (1.82 mmol) of HK_2_PO_4_ and 15 mg (0.14 mmol) of (Ir[dF(CF_3_)ppy]2(dtbpy))PF_6_ provided 135 mg (68%) of compound **37** as a separable 1:1 mixture of diastereomers and as colorless oils: Isomer 1: ^1^H NMR (500 MHz, CDCl_3_) δ 7.90 (dd, *J* = 7.8, 1.3 Hz, 1H), 7.54–7.44 (m, 1H), 7.07–6.97 (m, 2H), 4.41 (dt, *J* = 11.9, 4.5 Hz, 1H), 4.20 (q, *J* = 6.7 Hz, 1H), 3.95 (q, *J* = 6.7 Hz, 1H), 3.86 (q, *J* = 6.8 Hz, 1H), 2.90–2.72 (m, 2H), 2.21–2.07 (m, 1H), 1.95 (M, 3H). ^13^C-NMR (CDCl_3_, 125 MHz) δ 192.2, 161.4, 136.0, 126.9, 121.4, 121.1, 118.0, 79.7, 79.4, 69.0, 39.0, 27.6, 25.7. Isomer 2: ^1^H NMR (500 MHz, CDCl_3_) δ 7.90 (d, *J* = 7.8 Hz, 1H), 7.50 (t, *J* = 7.8 Hz, 1H), 7.14–6.97 (m, 2H), 4.43 (ddd, *J* = 13.1, 5.0, 2.9 Hz, 1H), 4.14 (q, *J* = 7.0 Hz, 1H), 4.03–3.72 (m, 2H), 2.93 (dd, *J* = 16.7, 13.2 Hz, 1H), 2.68 (dd, *J* = 16.7, 2.8 Hz, 1H), 2.15–1.83 (m, 4H). ^13^C-NMR (CDCl_3_, 125 MHz) δ 192.1, 161.3, 136.1, 126.8, 121.3, 120.9, 118.1, 79.8, 79.7, 68.9, 39.8, 27.5, 25.9.

*Preparation of tert-Butyl 2-(4-oxochroman-2-yl)piperidine-1-carboxylate* (**38**). According the the general procedure, reaction of 239 mg (1.64 mmol) of 4*H*-chromen-4-one **30** with 750 mg (3.27 mmol) of *N*-Boc-pipecolic acid **15** in the presence of 570 mg (3.27 mmol) of HK_2_PO_4_ and 18.3 mg (0.016 mmol) of (Ir[dF(CF_3_)ppy]2(dtbpy))PF_6_ provided 500 mg (92%) of compound **38** as a separable 1:1 mixture of diastereomers and rotamers: Isomer #1: colorless solid; ^1^H NMR (500 MHz, CDCl_3_) δ 7.92 (dd, 1H, *J* = 7.18 and 1.6 Hz), 7.50 (m, 1H), 7.05 (t, 1H, *J* = 7.5 Hz), 7.01 (d, 1H, *J* = 8.3 Hz), 4.74 (m, 1H), 4.50 (br m, 1H), 4.14 (br m, 1H), 2.75 (m, 3H), 2.23 (m, 1H), 1.75–1.60 (m, 5H), 1.49 (s, 9H); ^13^C-NMR (CDCl_3_, 125 MHz) δ 191.9, 161.1, 154.8, 135.9, 127.0, 121.5, 121.2, 117.9, 80.1, 74.8, 52.7, 39.9, 28.4, 25.1, 24.2, 19.2. Anal. Cald. For C_19_H_25_NO_4_: C, 68.86; H, 7.60; N, 4.23. Found: C, 68.96; H, 7.55; N, 4.19. Isomer #2: colorless oil; ^1^H NMR (500 MHz, CDCl_3_) δ 7.90 (dd, 1H, *J* = 7.9 and 1.6 Hz), 7.49 (m, 1H), 7.04 (m, 1H), 6.97 (d, 1H, *J* =8.3 Hz), 4.73 (q, 1H, *J* = 7.7 Hz), 4.50 (br m, 1H), 4.15 (br m, 1H), 2.96 (br m, 1H), 2.77 (m, 2H), 1.78 (m, 2H), 1.70 (m, 3H), 1.53 (m, 1H), 1.49 (s, 9H); ^13^C-NMR (CDCl_3_, 125 MHz) δ 192.0, 161.3, 155.5, 136.0, 126.8, 121.4, 120.9, 118.1, 79.6, 53.0, 40.7, 39.9, 28.4, 25.8, 25.0, 19.8. Anal.Calcd. for C_19_H_26_NO_4_ [M + H]: 332.1862. Found: 332.1870.

## 4. Conclusions

In conclusion, we have demonstrated that the photoredox-catalyzed decarboxylative formation of carbon-centered radicals from cyclic amino acids followed by conjugate addition to both cyclic vinylogous amides and esters provides access to novel heterocyclic structures. This versatile method is both mild and efficient giving rise to structural complexity previously inaccessible through current synthetic methodologies. Further manipulation of the products toward more complex synthetic targets is possible and will be disclosed in due course.

## Figures and Tables

**Table 1:** Scope of amino acid additions to dihydropyridone **5**.

Entry	Amino Acid	Product	Yield, dr
1	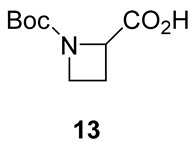	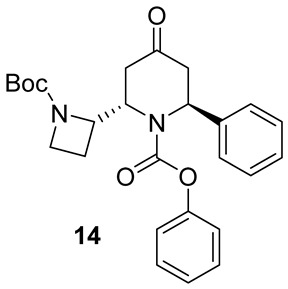	90%, 1:1
2	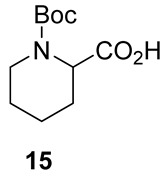	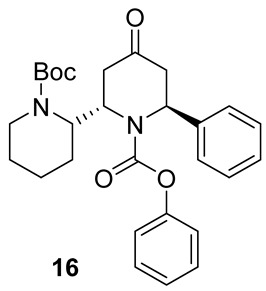	83%, 2.8:1
3	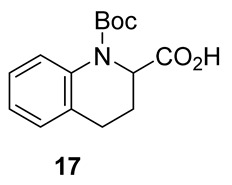	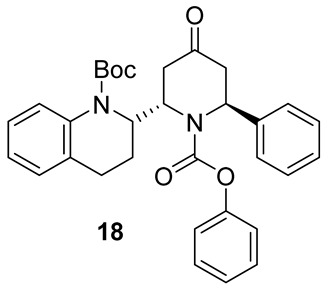	68%, 2.8:1
4	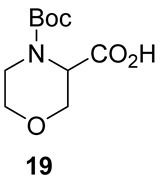	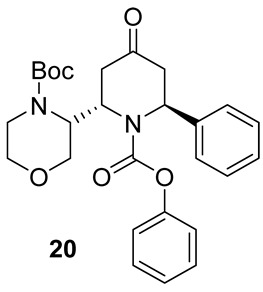	85%, 1:1
5	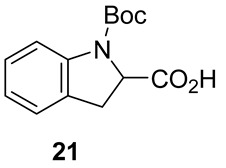	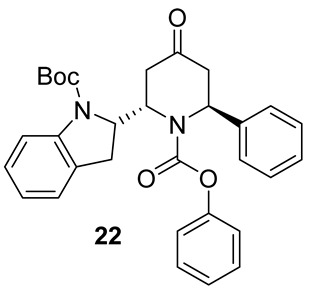	69%, 1:1
6	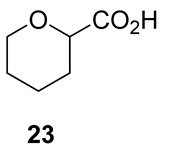	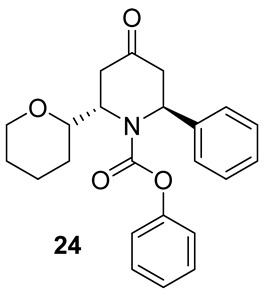	58%, 2:1
7	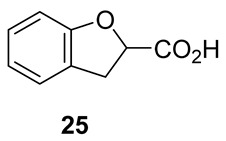	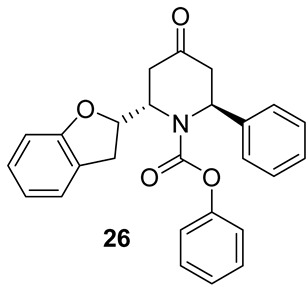	82%, 3:1

**Table 2:** Photocatalytic decarboxylative additions to 4-Oxoquinline **29** and chromenone **30**.

Entry	Starting Material	Amino Acid	Product	Yield, dr
1	**29**	**13**	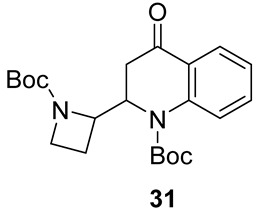	38%, 1:1
2	**29**	**11**	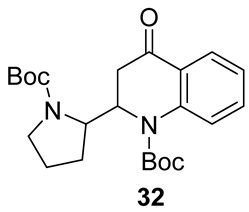	55%, 1:1
3	**29**	**14**	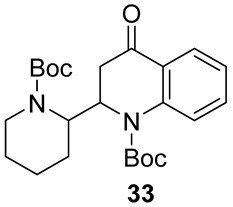	61%, 4:1
4	**29**	**16**	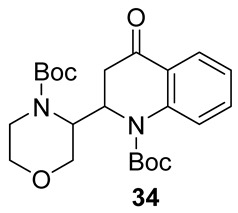	65%, 4:1
5	**30**	**11**	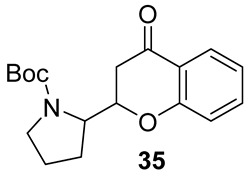	65%, 1:1
6	**30**	**21**	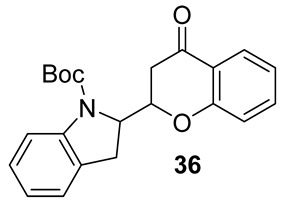	61%, 1:1
7	**30**	**27**	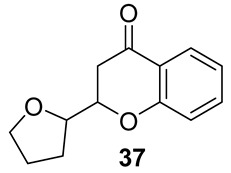	68%, 1:1
8	**30**	**15**	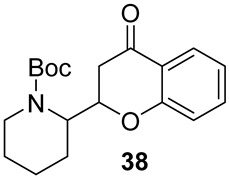	92%, 1:1

## Data Availability

Data are available in Electronic Supporting Information (ESI), and for additional details, please contact the authors.

## Supplementary Materials

The following supporting information can be downloaded. Full characterization (^1^H NMR and ^13^C NMR spectra) of all new compounds.
