# Supplementary material for: Photoredox-Catalyzed Giese Reactions: Decarboxylative Additions to Cyclic Vinylogous Amides and Esters
Source: Molecules. 2022 Jan 10;27(2):417. doi: 10.3390/molecules27020417 (PMC8777773; doi:10.3390/molecules27020417)
Supplement: Supplementary file 1 [file molecules-27-00417-s001.zip › molecules-1509025-supplementary.pdf]

Supplimentary Material

Photoredox-Catalyzed Giese Reactions: Decarboxylative Additions of Cyclic Vinylogous Amides and Esters

Kevin Dykstra, Alexei Buevich, Qi Gao, Yu-Hong Lam, and Jeffrey T. Kuethe*

1. ^{1}H NMR and ^{13}NMR of all new compounds pages 2-51
2. Characterization of Compound **8** pages 52-57
3. Characeterization of Compounds **9** and **10** pages 58-73
4. Characterization of Compound **26a** pages 74-80

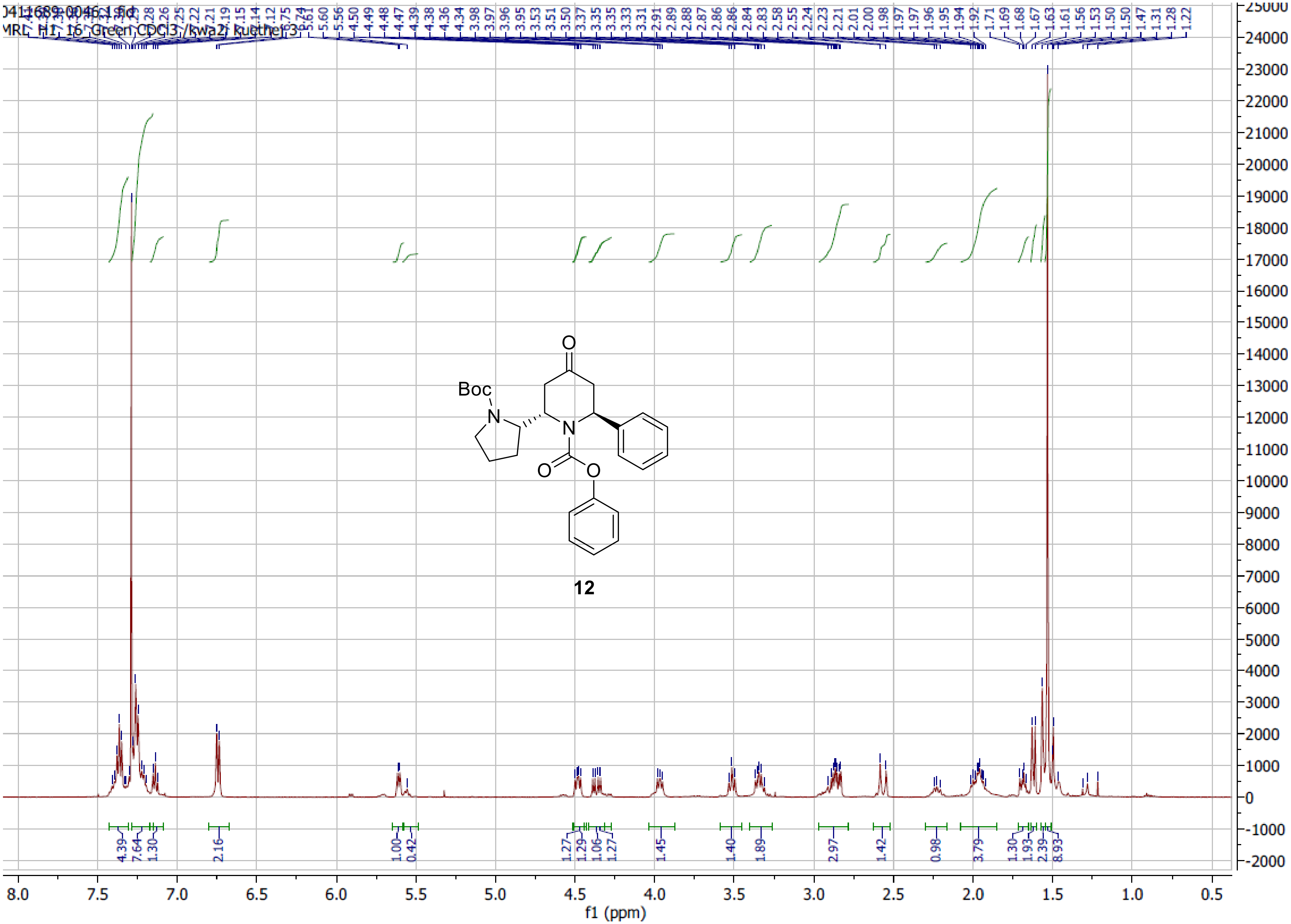

Boc
N
N
O
O
O
12
4.39
7.64
1.30
2.16
1.00
0.42
1.27
1.29
1.06
1.27
1.45
1.40
1.89
2.97
1.42
0.98
3.79
1.30
1.93
2.39
8.93
f1 (ppm)

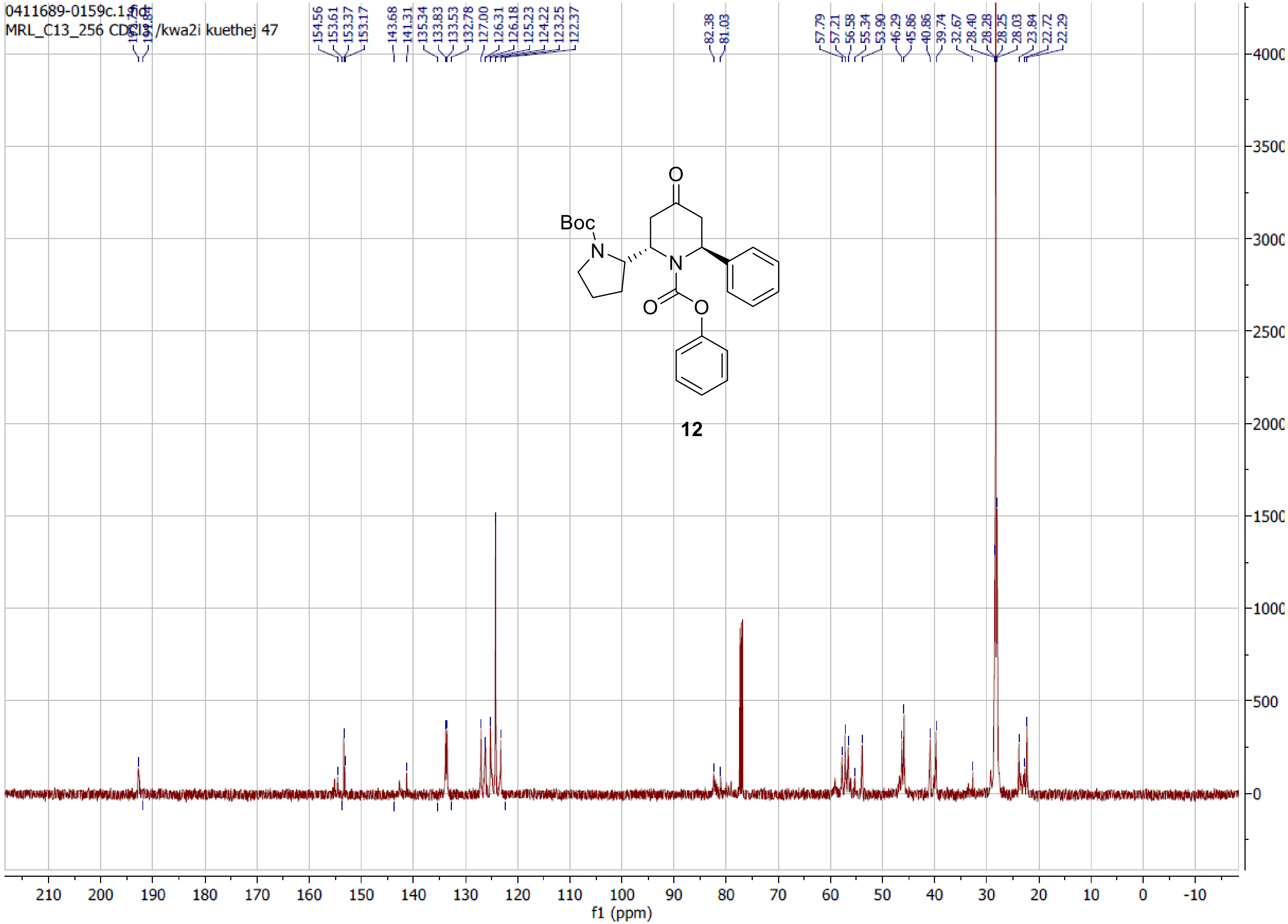

0411689-0159c.1.fid
MRL_C13_256 CDCl3 /kwa2i kuethej 47
154.56
153.61
153.37
153.17
143.68
141.31
135.34
133.83
133.53
132.78
127.00
126.31
126.18
125.23
124.22
123.25
122.37
82.38
81.03
57.79
57.21
56.58
55.34
53.90
46.29
45.86
40.86
39.74
32.67
28.40
28.28
28.25
28.03
23.84
22.72
22.29
Boc
N
O
N
O
O
12
f1 (ppm)

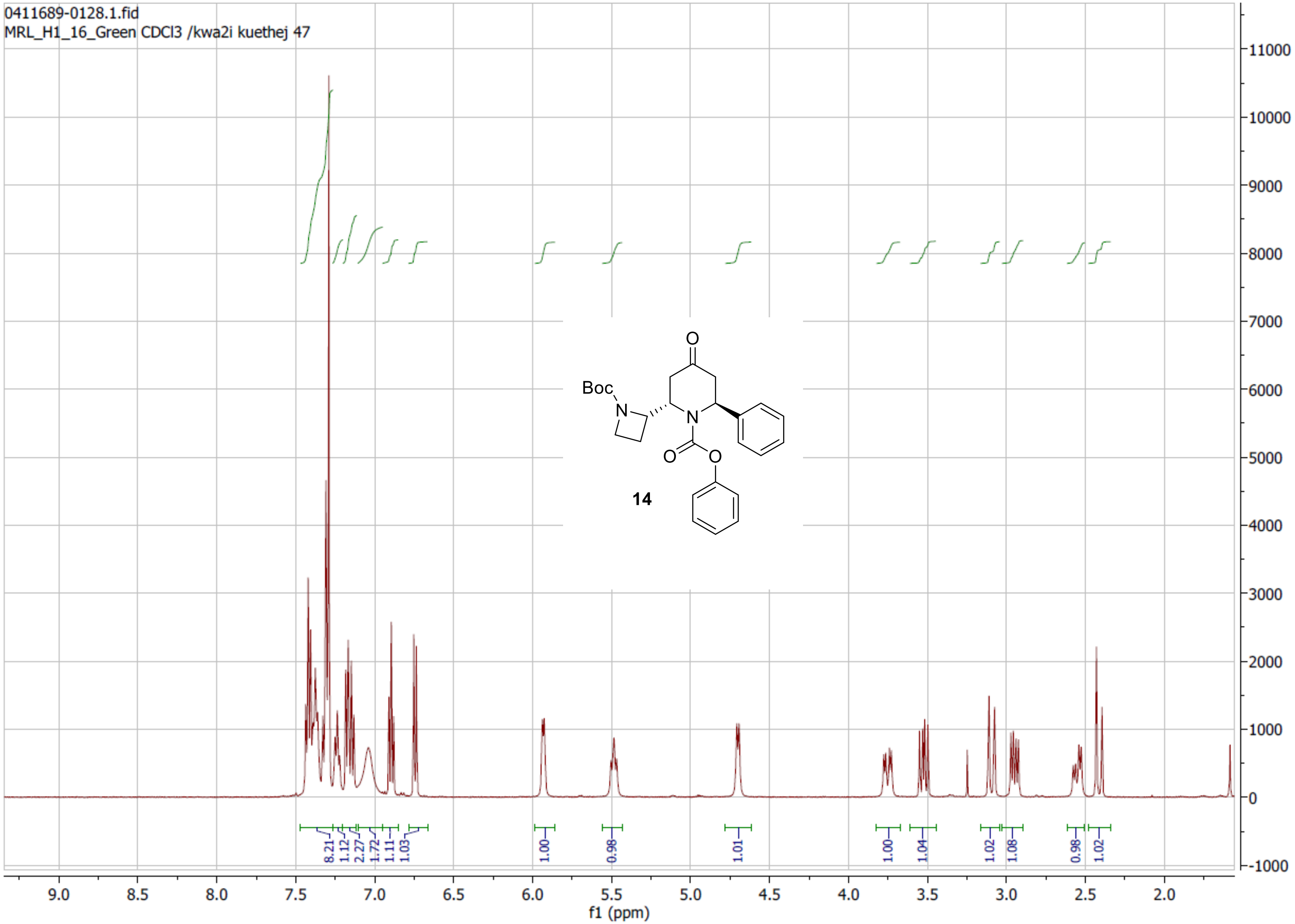
0411689-0128.1.fid
MRL_H1_16_Green CDCl3 /kwa2i kuethej 47
Boc
N
N
O
O
O
14
8.21
1.12
2.27
1.72
1.11
1.03
1.00
0.98
1.01
1.00
1.04
1.02
1.08
0.98
1.02
9.0
8.5
8.0
7.5
7.0
6.5
6.0
5.5
5.0
4.5
4.0
3.5
3.0
2.5
2.0
f1 (ppm)
11000
10000
9000
8000
7000
6000
5000
4000
3000
2000
1000
0
-1000

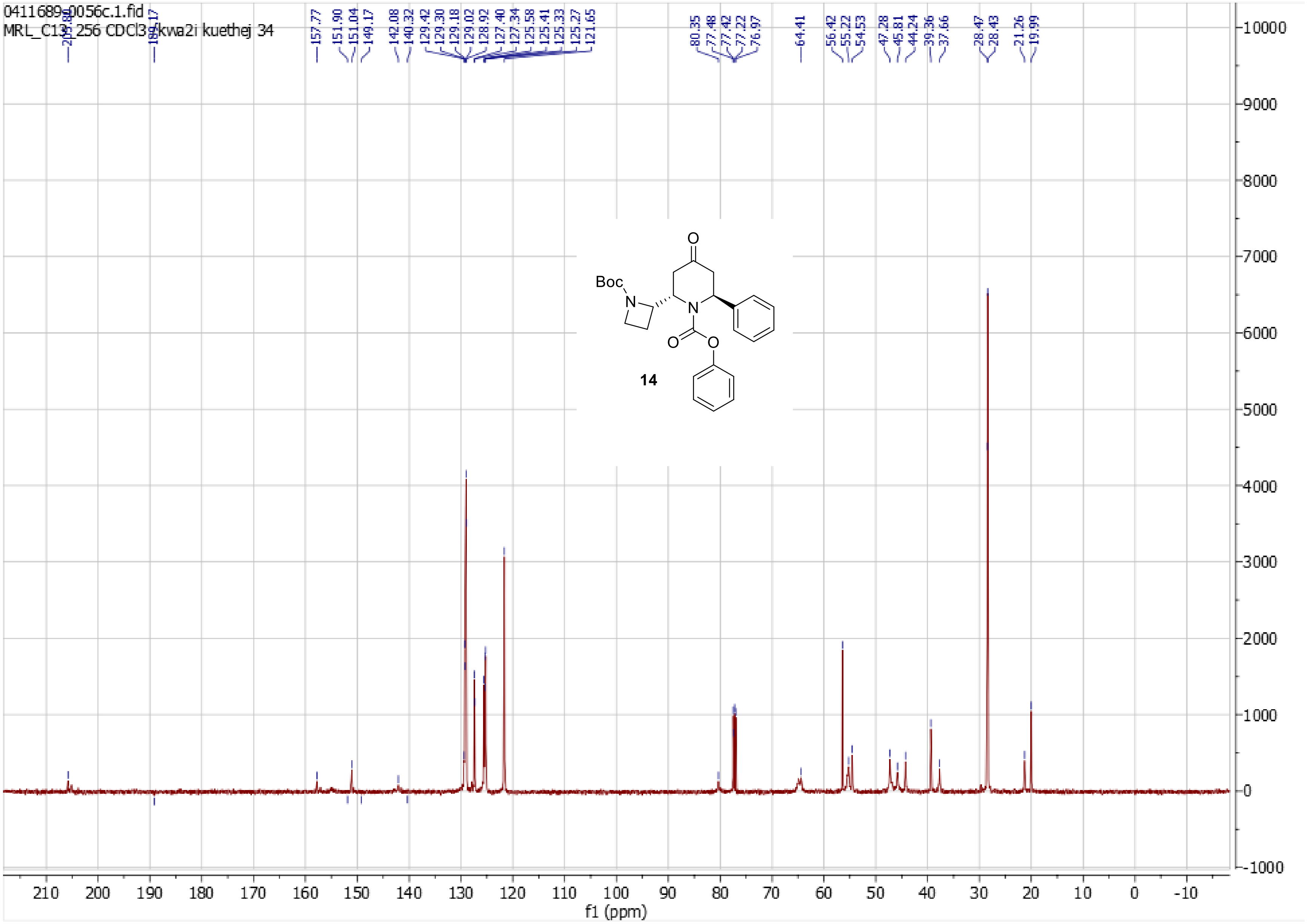
0411689-0056c.1.fid
MRL_C13_256 CDCl3 kwa2i kuethej 34
157.77
151.90
151.04
149.17
142.08
140.32
129.42
129.30
129.18
129.02
128.92
127.40
127.34
125.58
125.41
125.33
125.27
121.65
80.35
77.48
77.42
77.22
76.97
64.41
56.42
55.22
54.53
47.28
45.81
44.24
39.36
37.66
28.47
28.43
21.26
19.99
Boc
N
O
N
O
O
14
f1 (ppm)

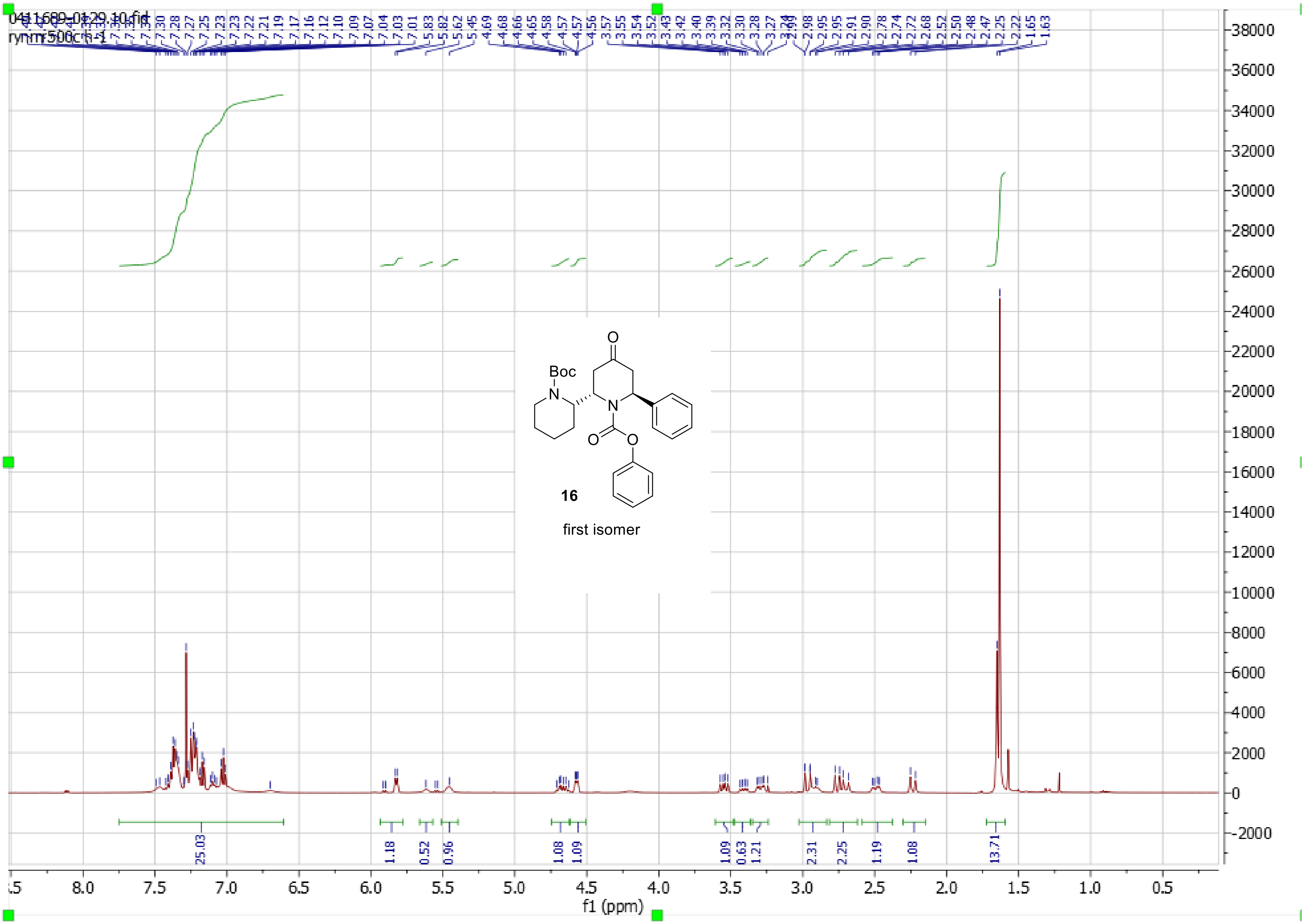

Boc
N
O
N
O
O
16
first isomer
f1 (ppm)

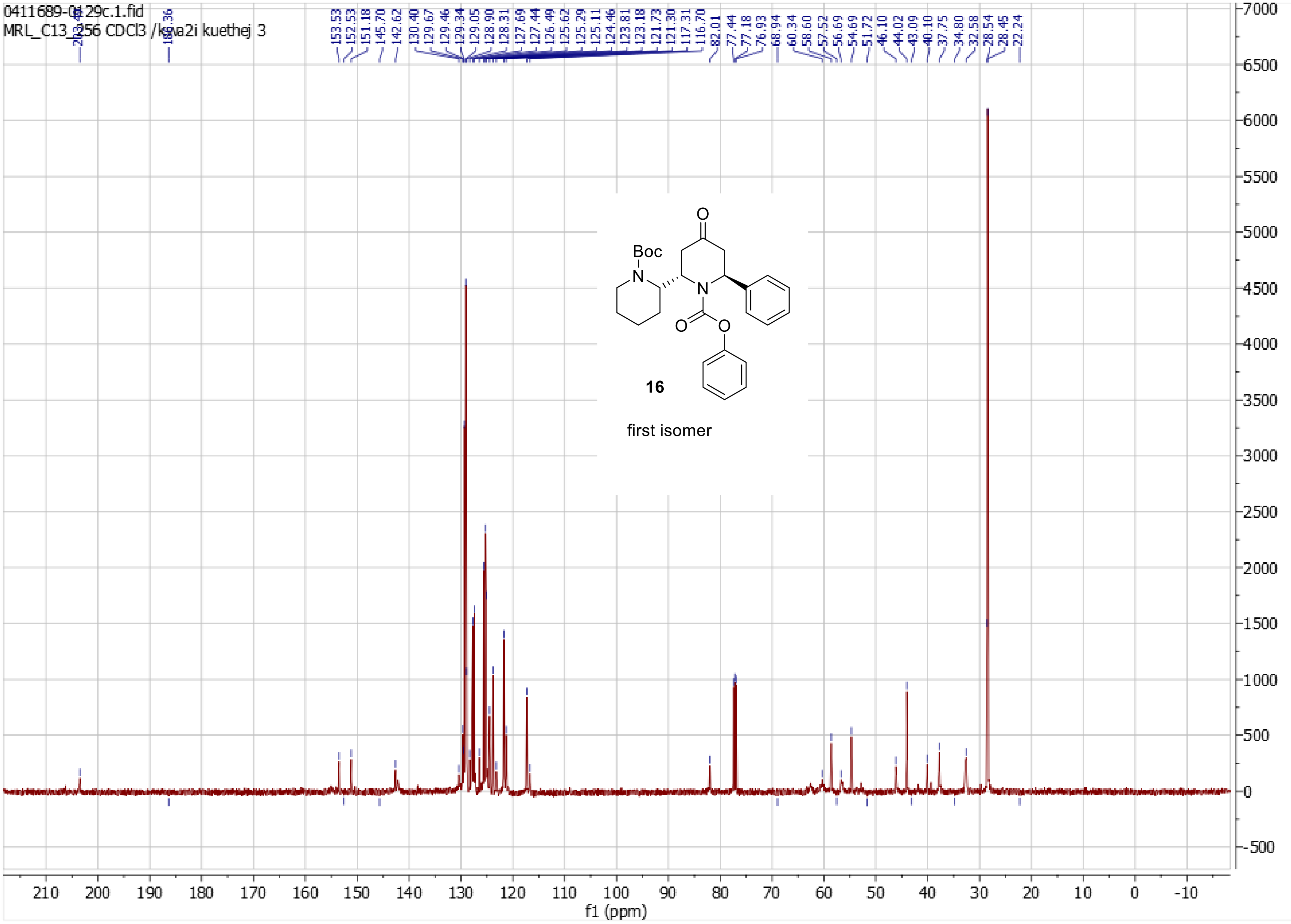

0411689-0129c.1.fid
MRL_C13_256 CDCl3 /kwa2i kuethej 3
153.53
152.53
151.18
145.70
142.62
130.40
129.67
129.46
129.34
129.05
128.90
128.31
127.69
127.44
126.49
125.62
125.29
125.11
124.46
123.81
123.18
121.73
121.30
117.31
116.70
82.01
77.44
77.18
76.93
68.94
60.34
58.60
57.52
56.69
54.69
51.72
46.10
44.02
43.09
40.10
37.75
34.80
32.58
28.54
28.45
22.24
Boc
N
O
16
first isomer
f1 (ppm)

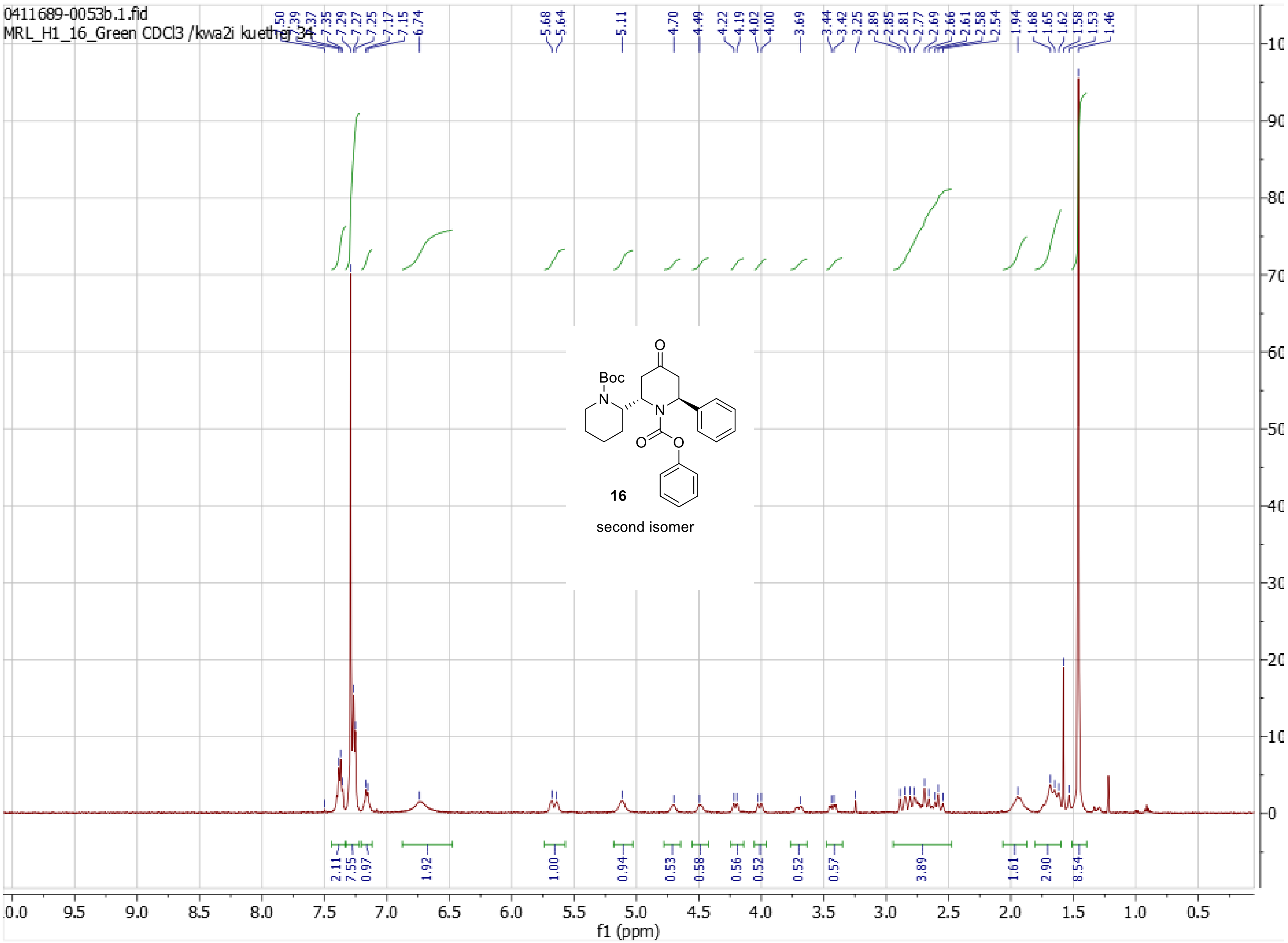

0411689-0053b.1.fid
MRL_H1_16_Green CDCl3 /kwa2i kuether 34
7.50 7.39 7.37 7.35 7.29 7.27 7.25 7.17 7.15 6.74
5.68 5.64
5.11
4.70
4.49
4.22 4.19 4.02 4.00
3.69
3.44 3.42 3.25
2.89 2.85 2.81 2.77 2.69 2.66 2.61 2.58 2.54
1.94
1.68 1.65 1.62 1.58 1.53 1.46
Boc
N
O
16
second isomer
2.11 7.55 0.97 1.92 1.00 0.94 0.53 0.58 0.56 0.52 0.52 0.57 3.89 1.61 2.90 8.54
f1 (ppm)

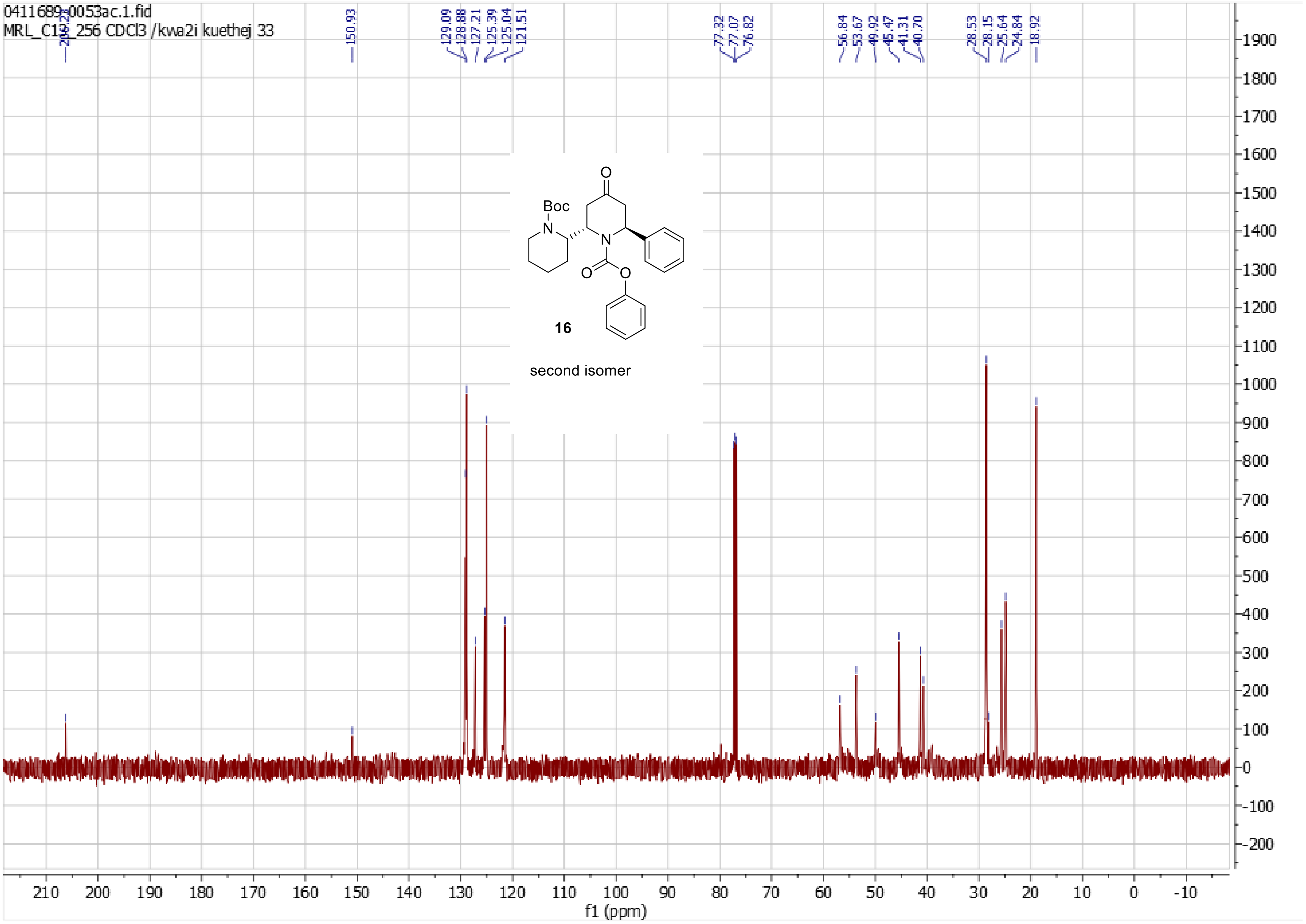
0411689-0053ac.1.fid
MRL_C13_256 CDCl3 /kwa2i kuethej 33
150.93
129.09
128.88
127.21
125.39
125.04
121.51
77.32
77.07
76.82
56.84
53.67
49.92
45.47
41.31
40.70
28.53
28.15
25.64
24.84
18.92
Boc
16
second isomer
f1 (ppm)

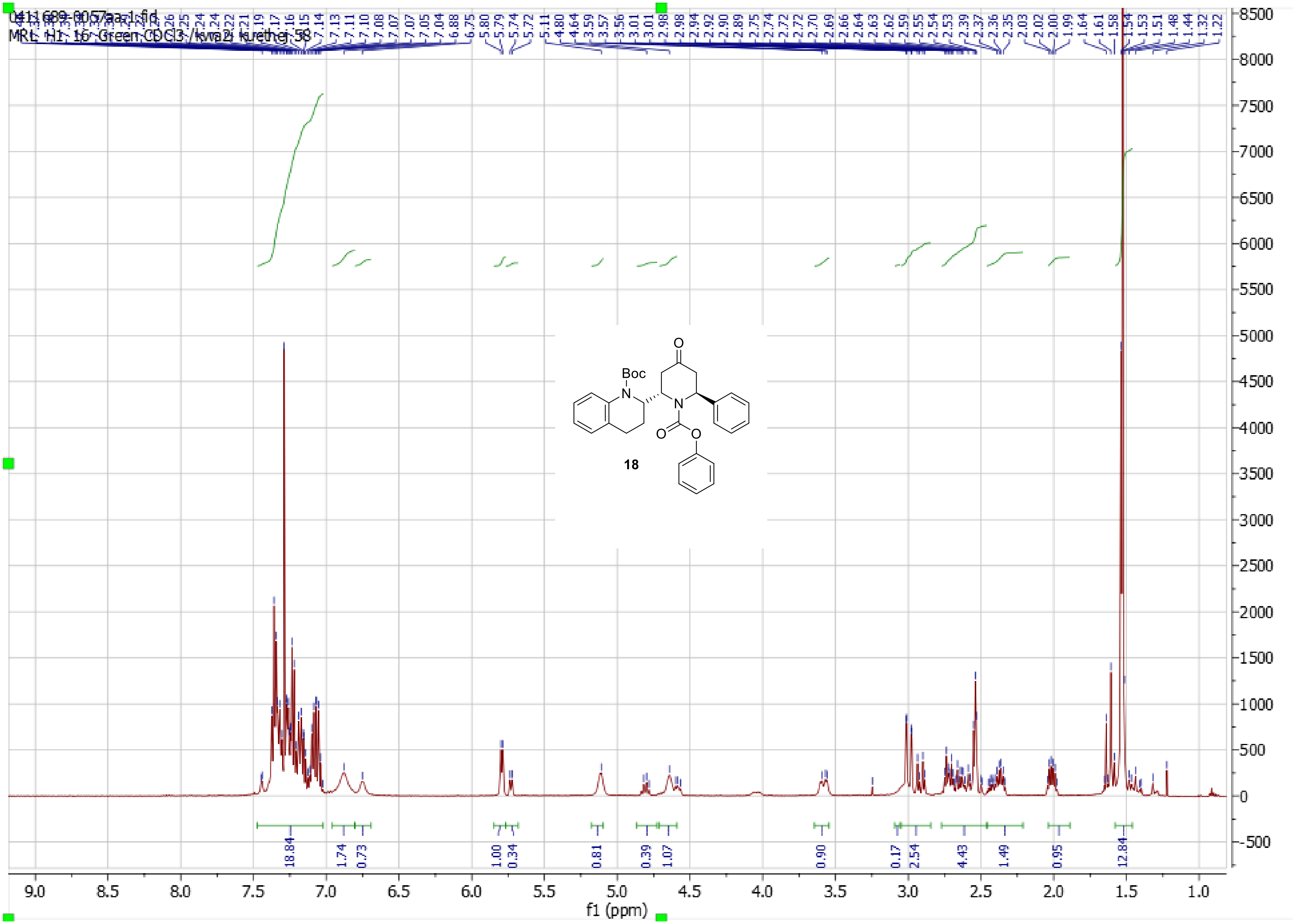
Boc
N
N
O
O
O
18
18.84
1.74
0.73
1.00
0.34
0.81
0.39
1.07
0.90
0.17
2.54
4.43
1.49
0.95
12.84
f1 (ppm)

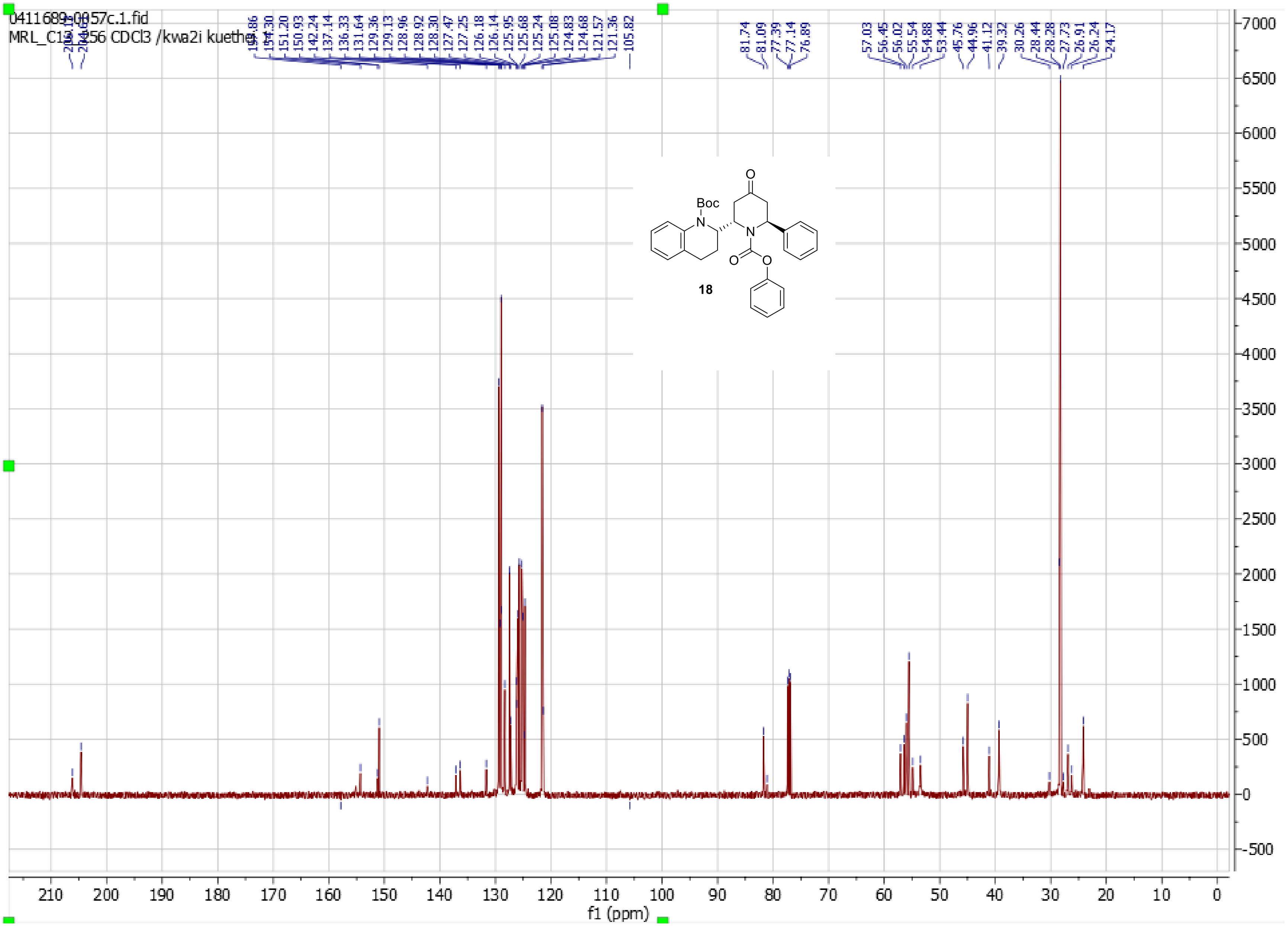

0411689-0057c.1.fid
MRL_C13_256 CDCl3 /kwa2i kuethe 1
154.30
151.20
150.93
142.24
137.14
136.33
131.64
129.36
129.13
128.96
128.92
128.30
127.47
127.25
126.18
126.14
125.95
125.68
125.24
125.08
124.83
124.68
121.57
121.36
105.82
81.74
81.09
77.39
77.14
76.89
57.03
56.45
56.02
55.54
54.88
53.44
45.76
44.96
41.12
39.32
30.26
28.44
28.28
27.73
26.91
26.24
24.17
Boc
N
O
18
f1 (ppm)

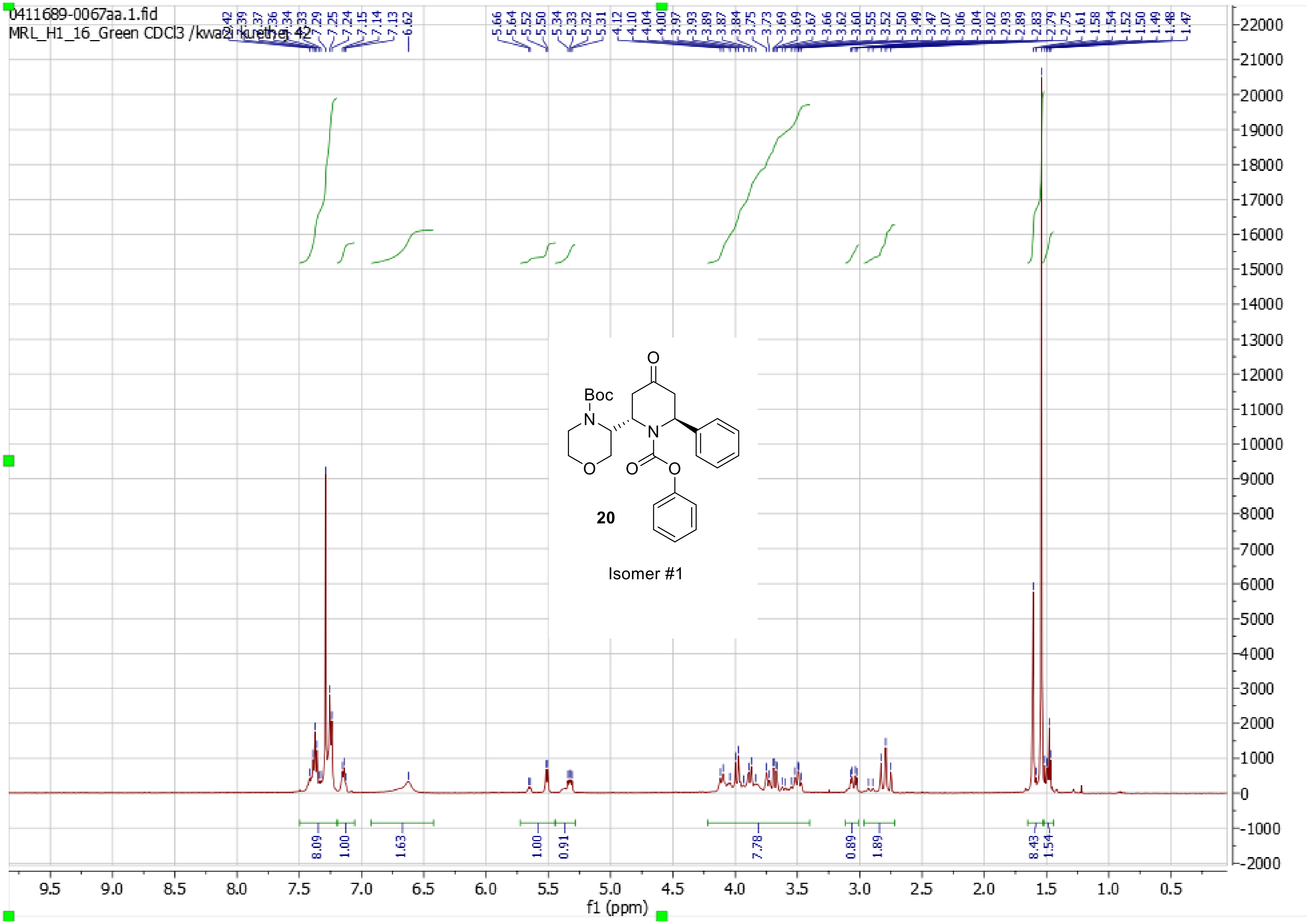
0411689-0067aa.1.fid
MRL_H1_16_Green CDCl3 /kwa2i kuethe 42
7.42 7.39 7.37 7.36 7.34 7.33 7.29 7.25 7.24 7.15 7.14 7.13 6.62
5.66 5.64 5.52 5.50 5.34 5.33 5.32 5.31 4.12 4.10 4.04 4.00 3.97 3.93 3.89 3.87 3.84 3.75 3.73 3.69 3.69 3.67 3.66 3.62 3.60 3.55 3.52 3.50 3.49 3.47 3.07 3.06 3.04 3.02 2.93 2.89 2.83 2.79 2.75 1.61 1.58 1.54 1.52 1.50 1.49 1.48 1.47
O
Boc
N
N
O
O
O
20
Isomer #1
8.09 1.00 1.63 1.00 0.91 7.78 0.89 1.89 8.43 1.54
f1 (ppm)

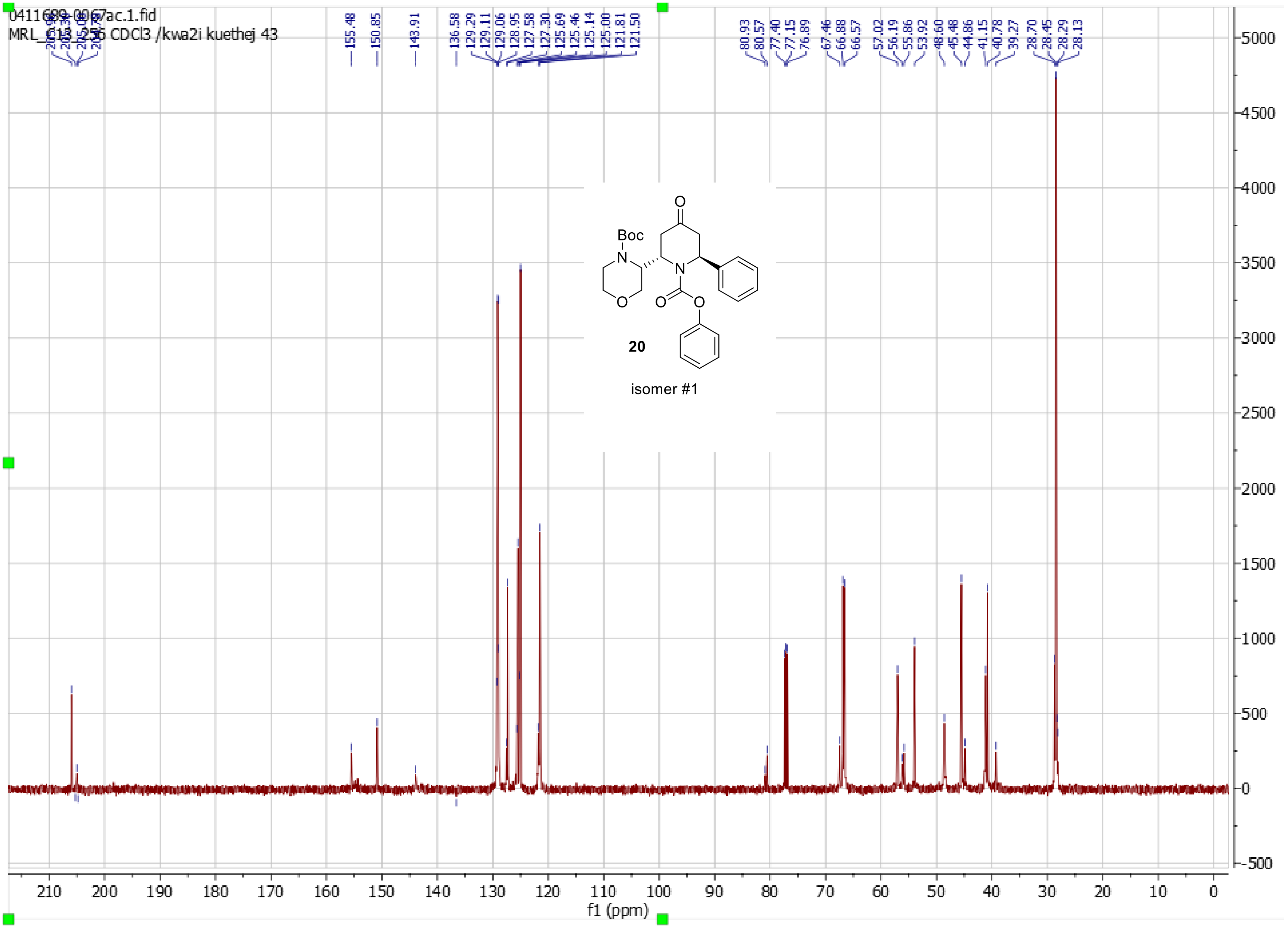
0411689-0067ac.1.fid
MRL_C13_256 CDCl3 /kwa2i kuethej 43
155.48
150.85
143.91
136.58
129.29
129.11
129.06
128.95
127.58
127.30
125.69
125.46
125.14
125.00
121.81
121.50
80.93
80.57
77.40
77.15
76.89
67.46
66.88
66.57
57.02
56.19
55.86
53.92
48.60
45.48
44.86
41.15
40.78
39.27
28.70
28.45
28.29
28.13
Boc
N
O
N
O
O
O
20
isomer #1
f1 (ppm)

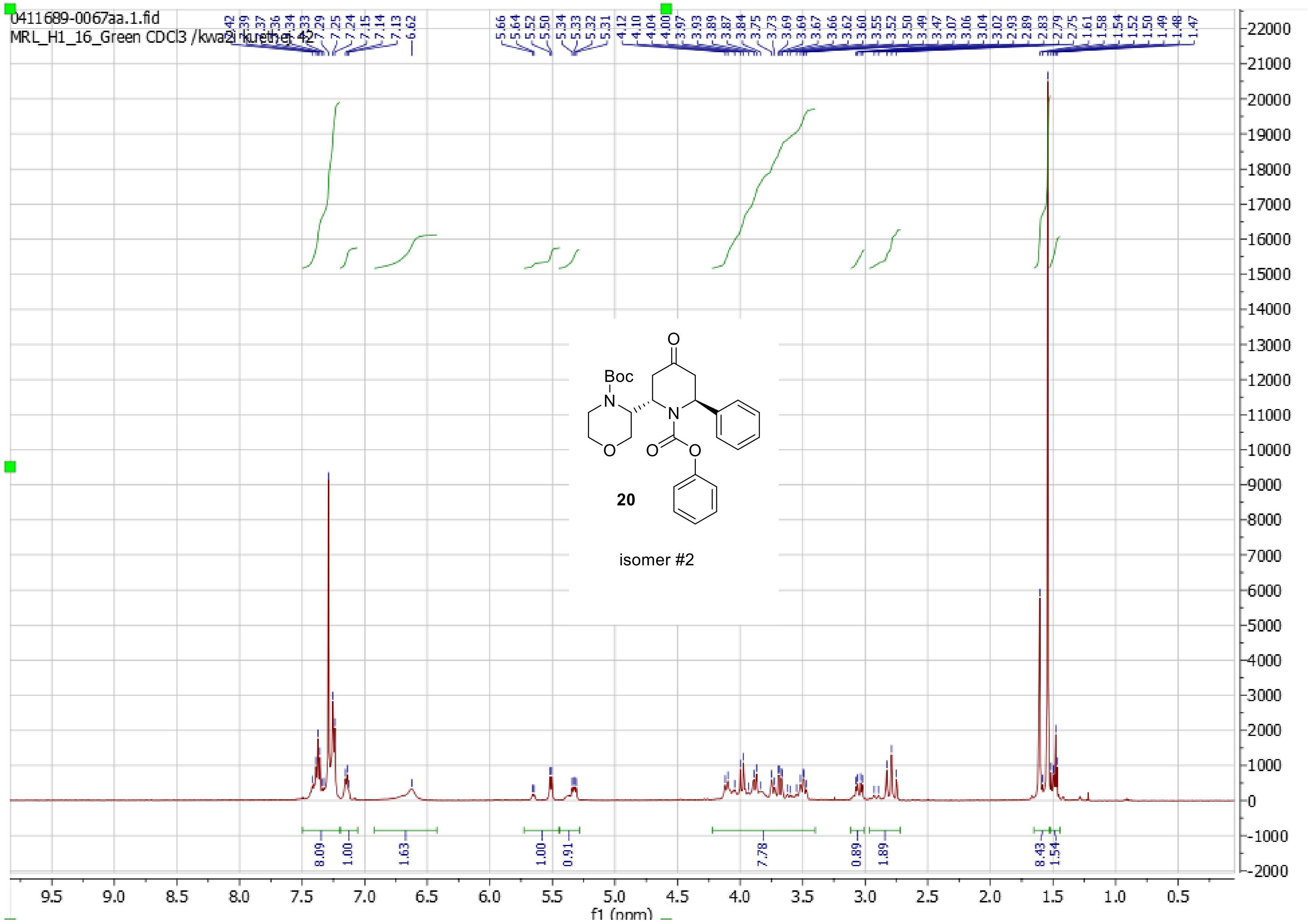
0411689-0067aa.1.fid
MRL_H1_16_Green CDCl3 /kwa2i kurethei 42
20
isomer #2
f1 (ppm)

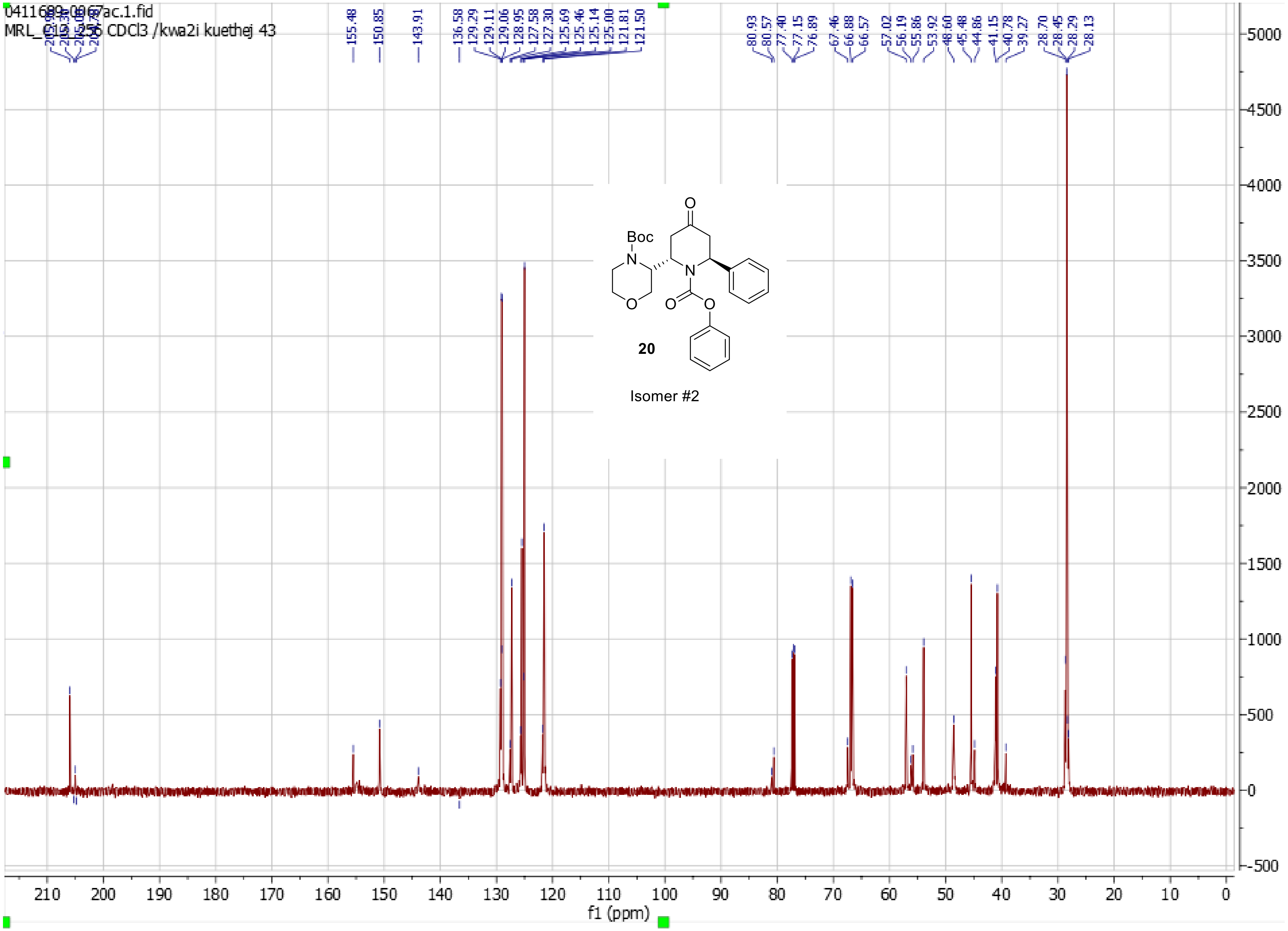

0411689-0067ac.1.fid
MRL_C13_256 CDCl3 /kwa2i kuethej 43
155.48
150.85
143.91
136.58
129.29
129.11
129.06
128.95
127.58
127.30
125.69
125.46
125.14
125.00
121.81
121.50
80.93
80.57
77.40
77.15
76.89
67.46
66.88
66.57
57.02
56.19
55.86
53.92
48.60
45.48
44.86
41.15
40.78
39.27
28.70
28.45
28.29
28.13
Boc
N
O
N
O
O
O
20
Isomer #2
f1 (ppm)

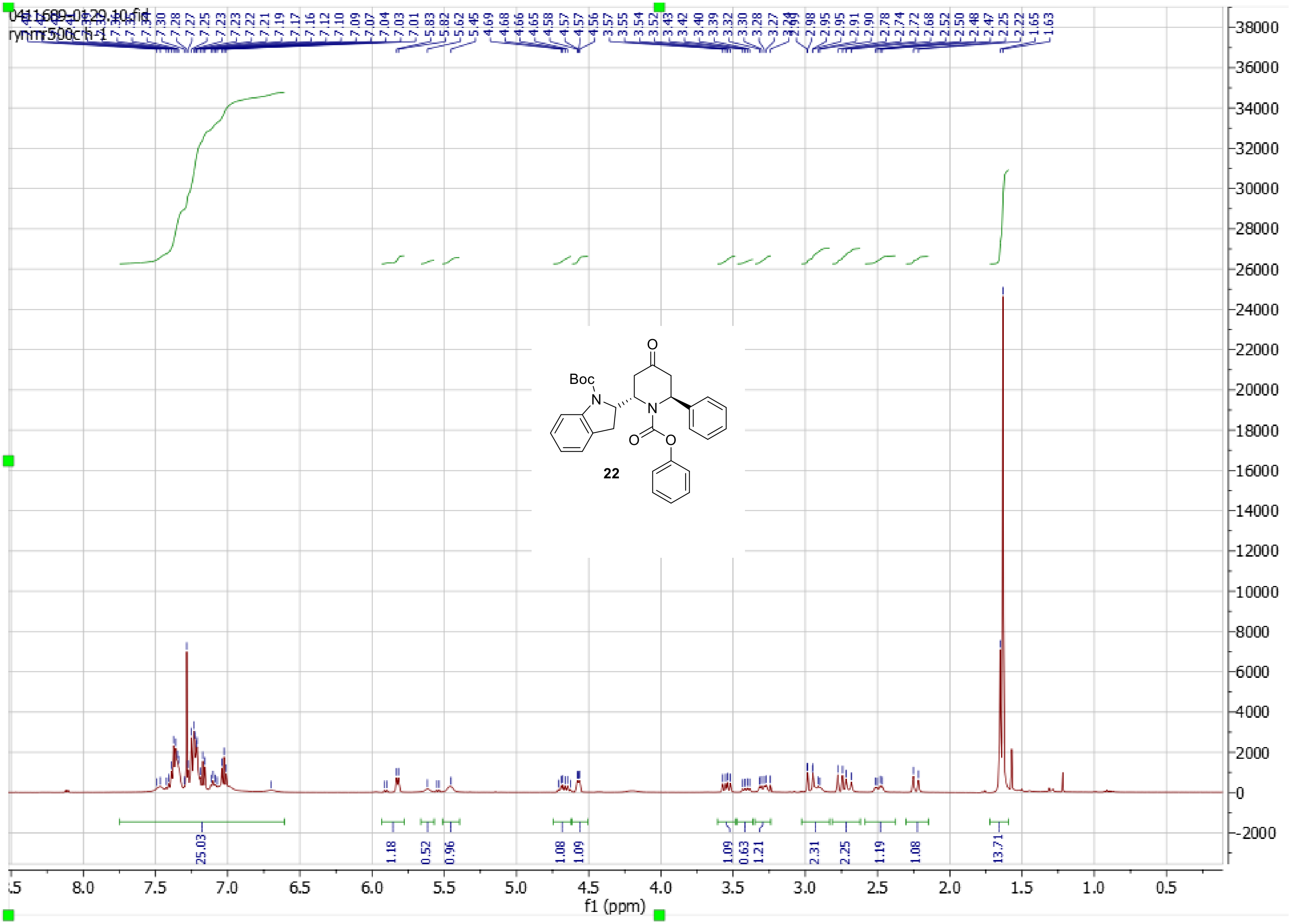

rynmr500cr-1
Boc
N
N
O
O
O
22
25.03
1.18
0.52
0.96
1.08
1.09
1.09
0.63
1.21
2.31
2.25
1.19
1.08
13.71
8.0
7.5
7.0
6.5
6.0
5.5
5.0
4.5
4.0
3.5
3.0
2.5
2.0
1.5
1.0
0.5
f1 (ppm)
38000
36000
34000
32000
30000
28000
26000
24000
22000
20000
18000
16000
14000
12000
10000
8000
6000
4000
2000
0
-2000

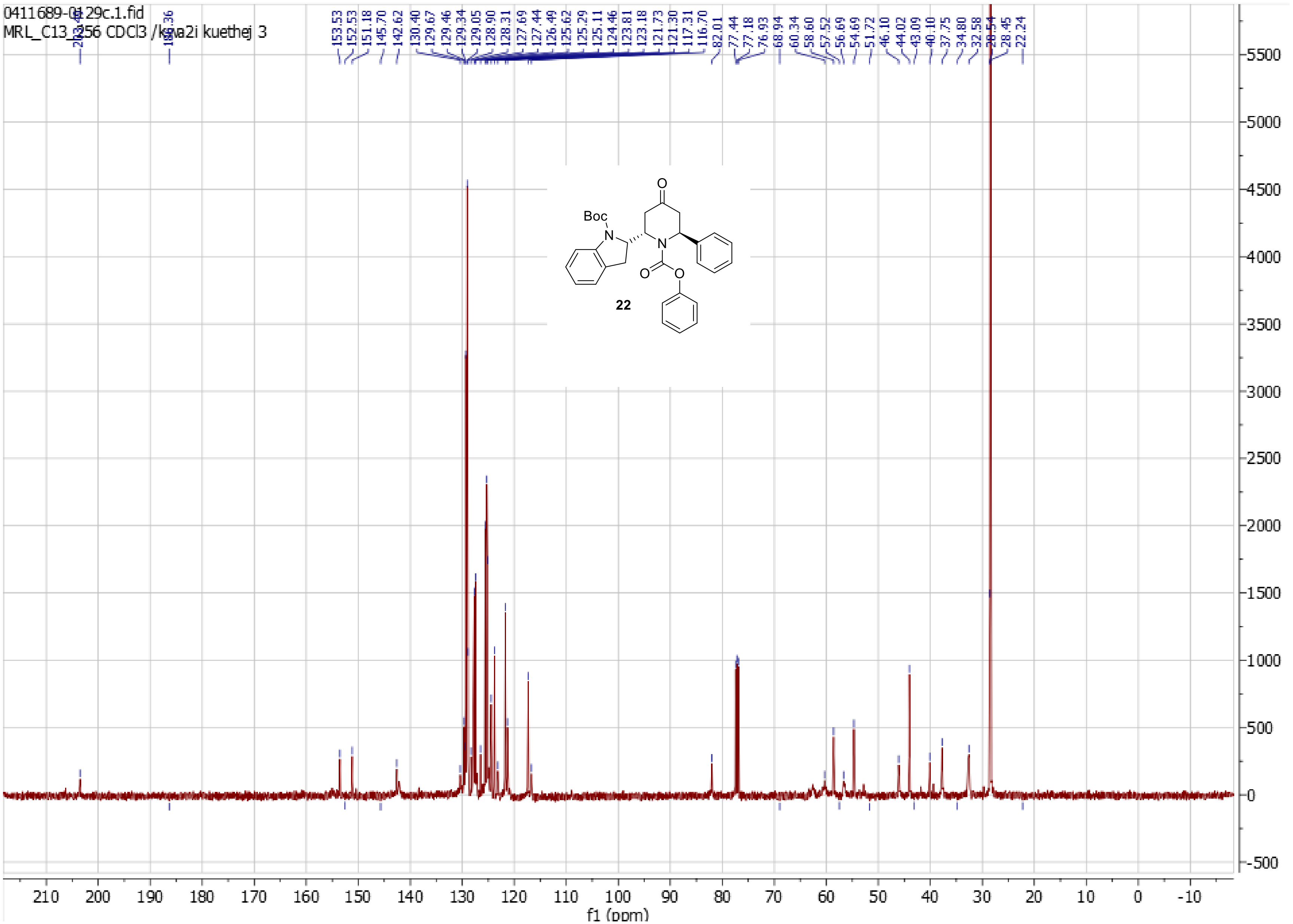
0411689-0129c.1.fid
MRL_C13_256 CDCl3 /kgva2i kuethej 3
153.53
152.53
151.18
145.70
142.62
130.40
129.67
129.46
129.34
129.05
128.90
128.31
127.69
127.44
126.49
125.62
125.29
125.11
124.46
123.81
123.18
121.73
121.30
117.31
116.70
82.01
77.44
77.18
76.93
68.94
60.34
58.60
57.52
56.69
54.69
51.72
46.10
44.02
43.09
40.10
37.75
34.80
32.58
28.54
28.45
22.24
Boc
N
O
22
f1 (ppm)

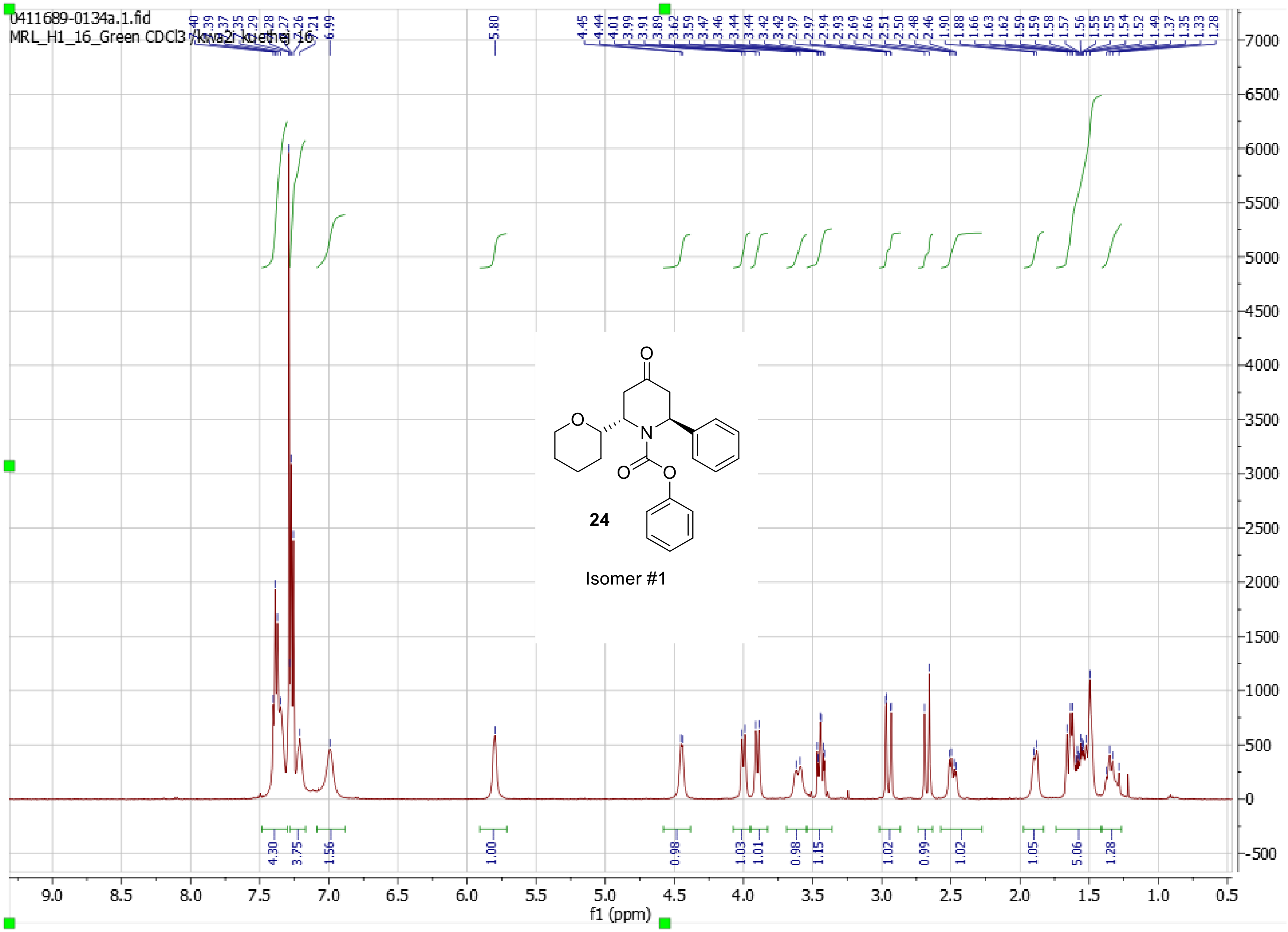
0411689-0134a.1.fid
MRL_H1_16_Green CDCl3
24
Isomer #1
f1 (ppm)
4.30
3.75
1.56
1.00
0.98
1.03
1.01
0.98
1.15
1.02
0.99
1.02
1.05
5.06
1.28

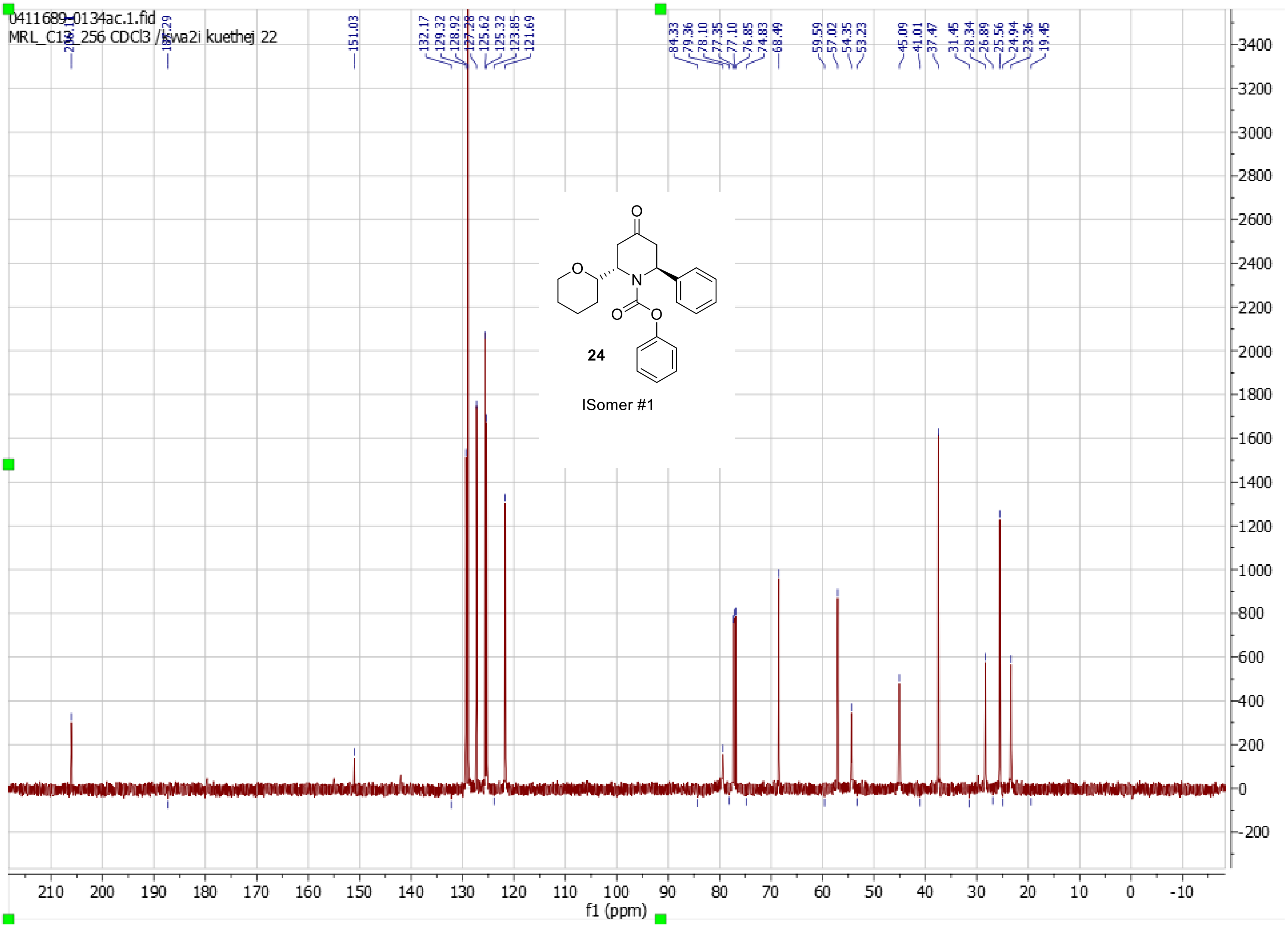

0411689-0134ac.1.fid
MRL_C13_256 CDCl3 /kwa2i kuethej 22
151.03
132.17
129.32
128.92
127.28
125.62
125.32
123.85
121.69
84.33
79.36
78.10
77.35
77.10
76.85
74.83
68.49
59.59
57.02
54.35
53.23
45.09
41.01
37.47
31.45
28.34
26.89
25.56
24.94
23.36
19.45
O
N
O
O
O
24
ISomer #1
f1 (ppm)

0411689-0134b.1.fid
MRL_H1_16_Green CDCl3 /kwa2i kuethej 24

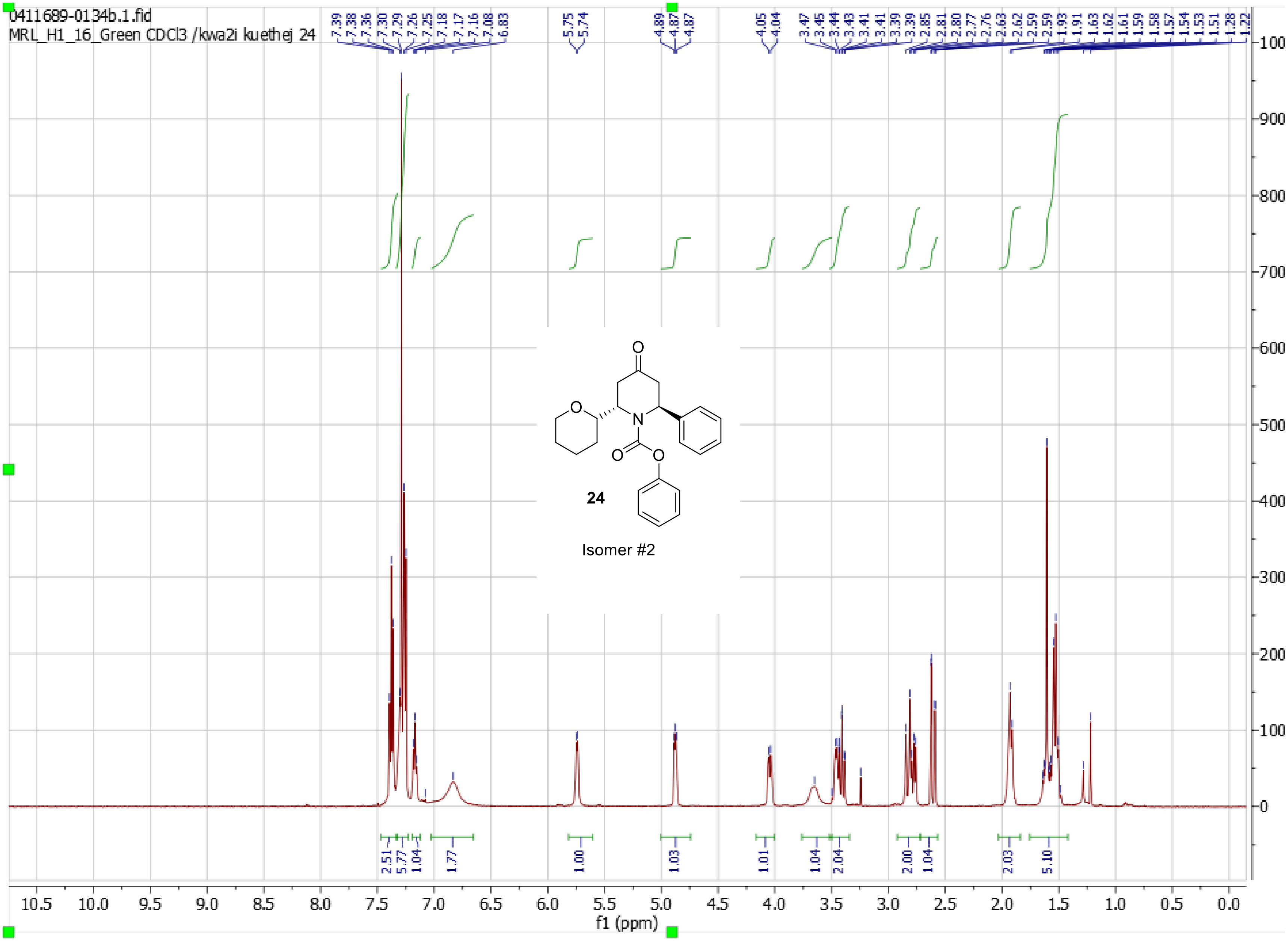

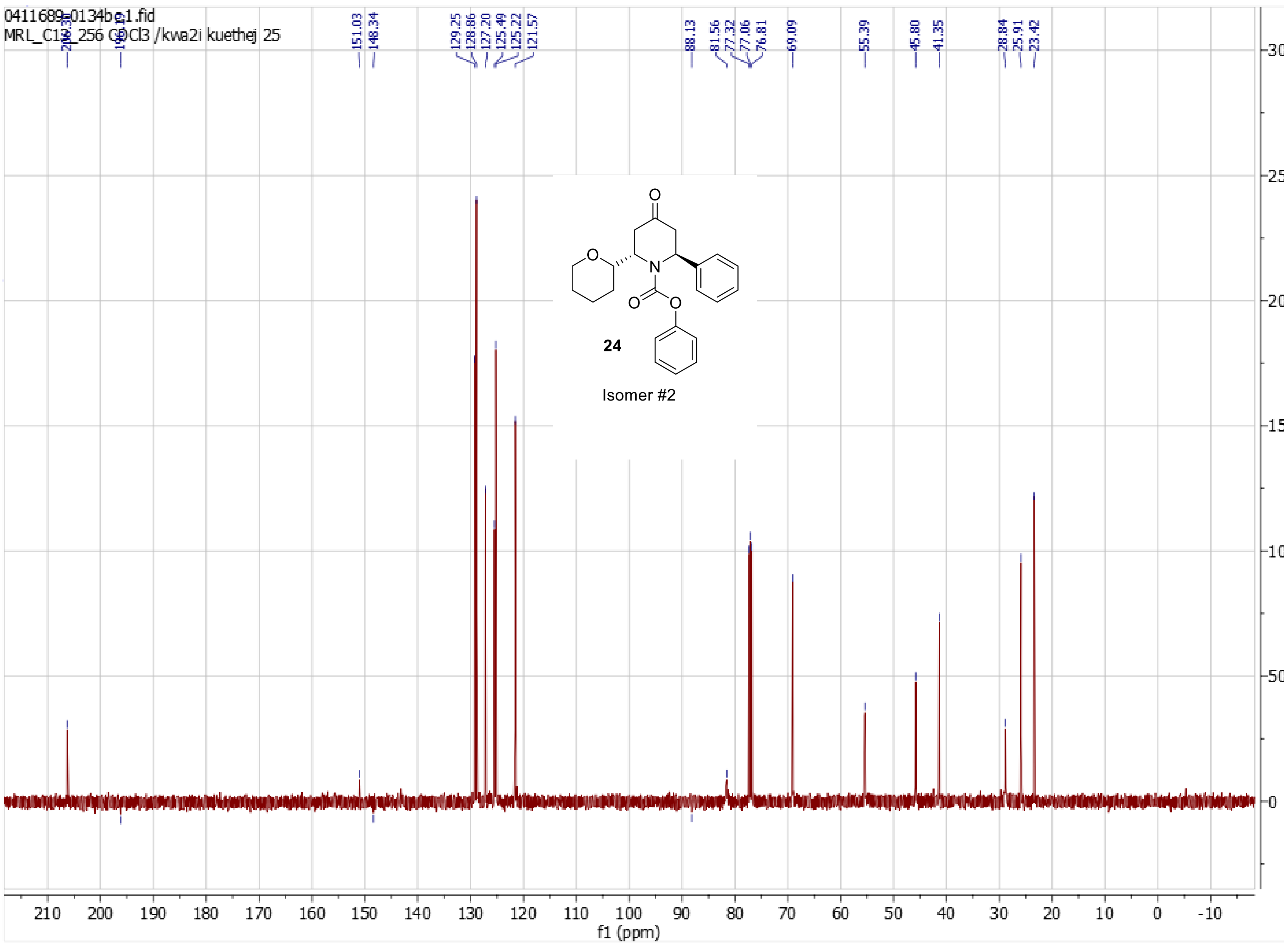
0411689-0134bc1.fid
MRL_C13_256 CDCl3 /kwa2i kuethej 25
151.03
148.34
129.25
128.86
127.20
125.49
125.22
121.57
88.13
81.56
77.32
77.06
76.81
69.09
55.39
45.80
41.35
28.84
25.91
23.42
24
Isomer #2
f1 (ppm)

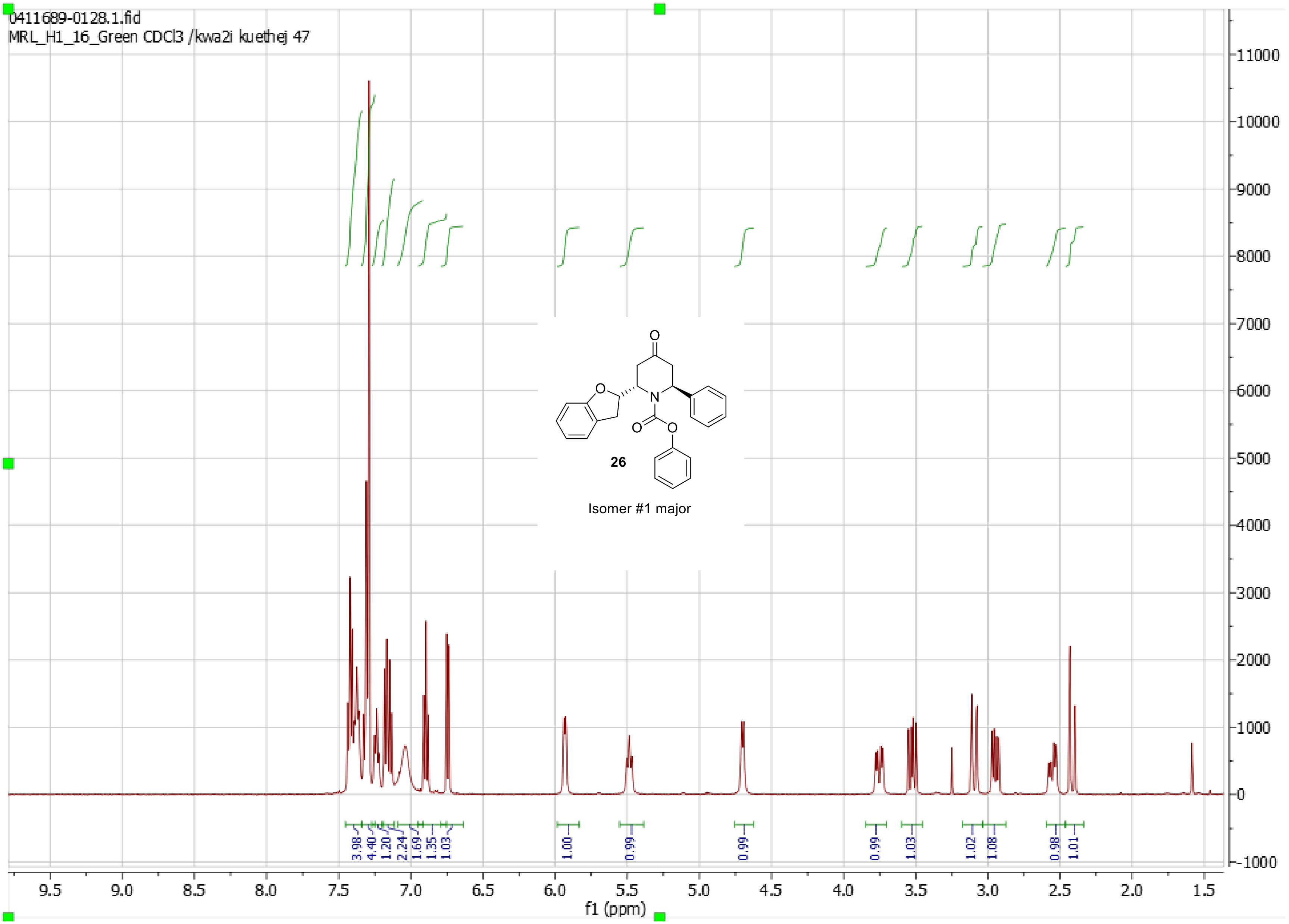

0411689-0128.1.fid
MRL_H1_16_Green CDCl3 /kwa2i kuethej 47
26
Isomer #1 major
3.98
4.40
1.20
2.24
1.69
1.35
1.03
1.00
0.99
0.99
0.99
1.03
1.02
1.08
0.98
1.01
f1 (ppm)

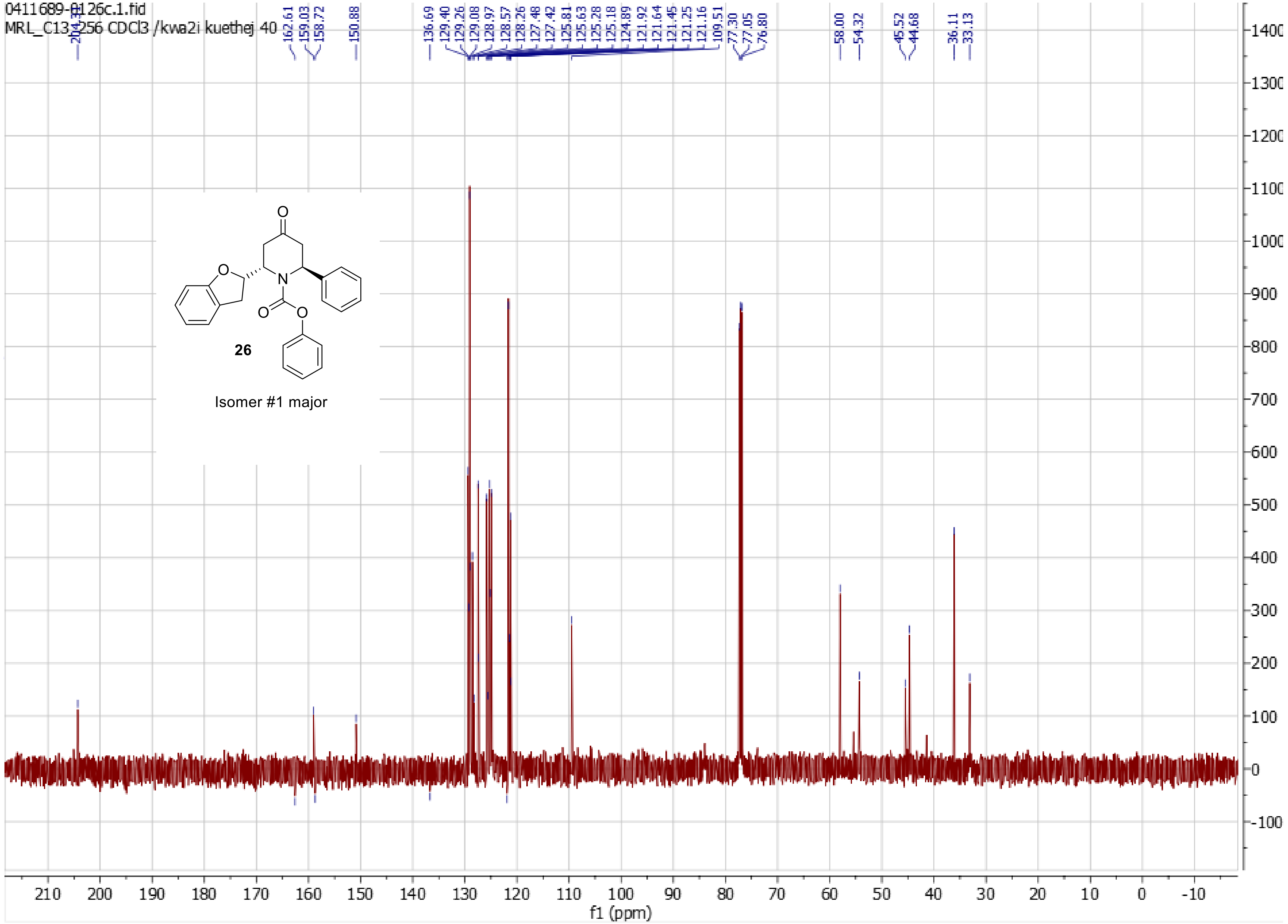
0411689-0126c.1.fid
MRL_C13_256 CDCl3 /kwa2i kuethej 40
204.33
162.61
159.03
158.72
150.88
136.69
129.40
129.26
129.08
128.97
128.57
128.26
127.48
127.42
125.81
125.63
125.28
125.18
124.89
121.92
121.64
121.45
121.25
121.16
109.51
77.30
77.05
76.80
58.00
54.32
45.52
44.68
36.11
33.13
O
O
N
O
O
26
Isomer #1 major
f1 (ppm)

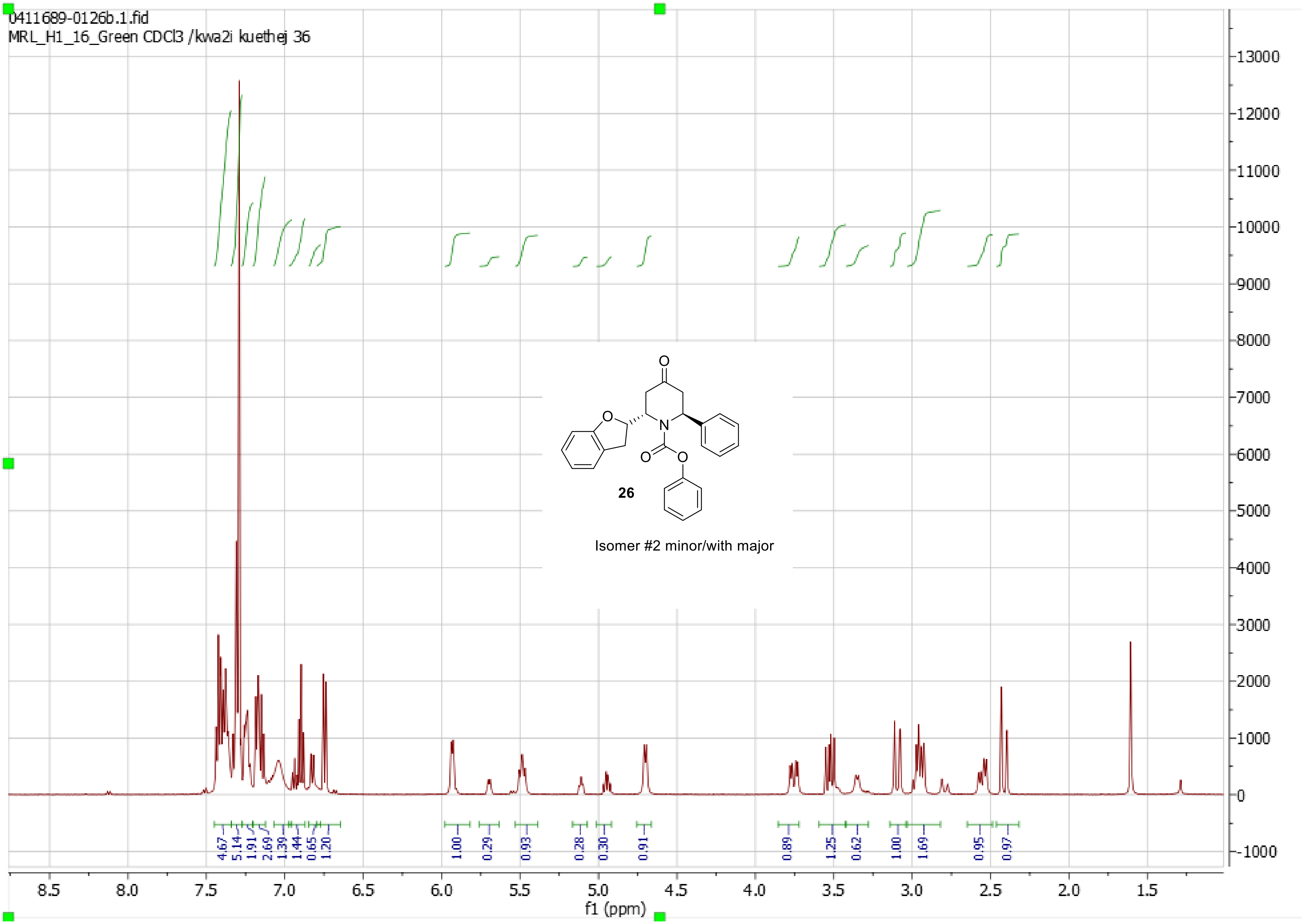

0411689-0126b.1.fid
MRL_H1_16_Green CDCl3 /kwa2i kuethej 36
26
Isomer #2 minor/with major
4.67
5.14
1.91
2.69
1.39
1.44
0.65
1.20
1.00
0.29
0.93
0.28
0.30
0.91
0.89
1.25
0.62
1.00
1.69
0.95
0.97
f1 (ppm)

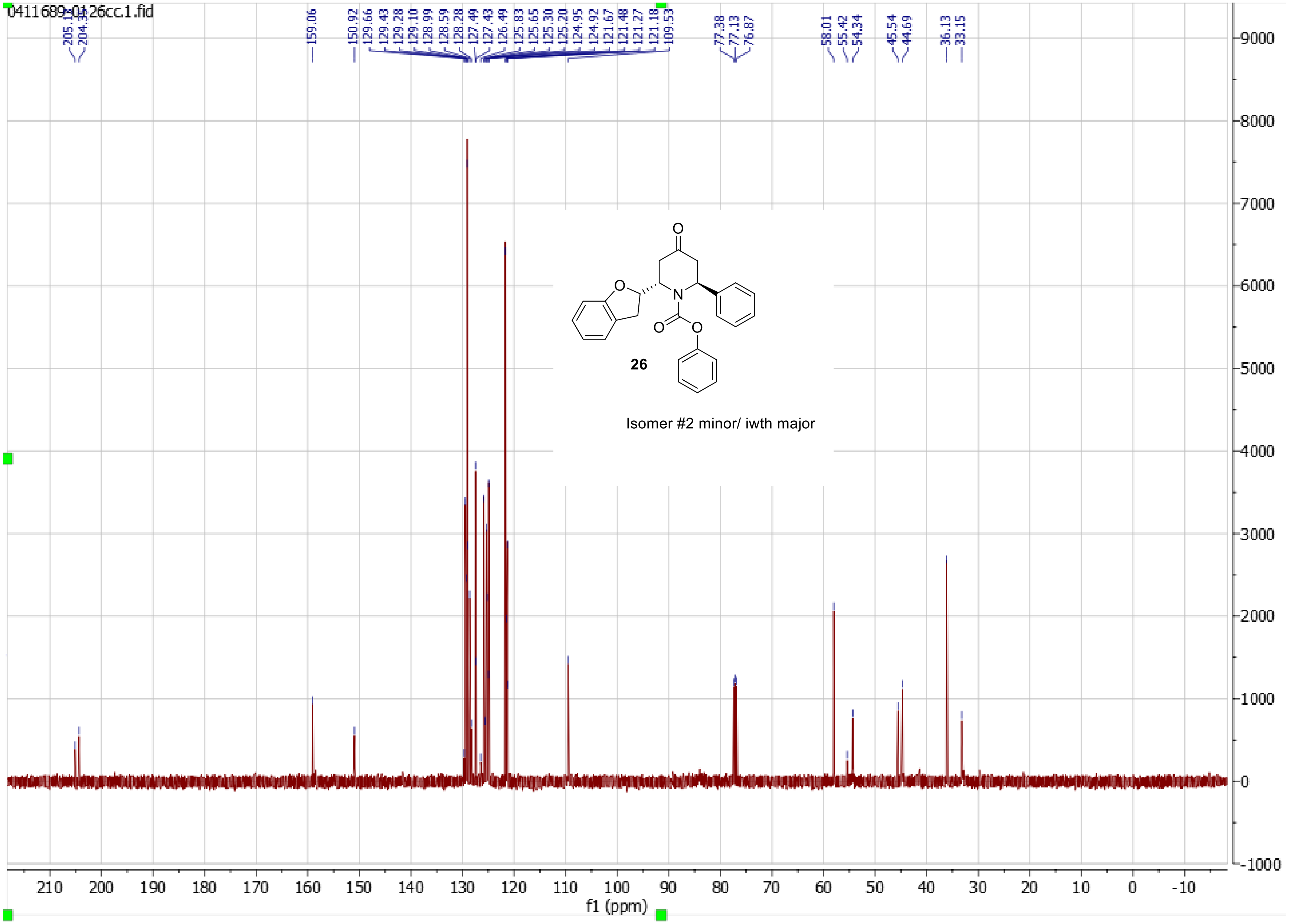
0411689-0126cc.1.fid
205.1
204.3
159.06
150.92
129.66
129.43
129.28
129.10
128.99
128.59
128.28
127.49
127.43
126.49
125.83
125.65
125.30
125.20
124.95
124.92
121.67
121.48
121.27
121.18
109.53
77.38
77.13
76.87
58.01
55.42
54.34
45.54
44.69
36.13
33.15
26
Isomer #2 minor/ iwth major
f1 (ppm)

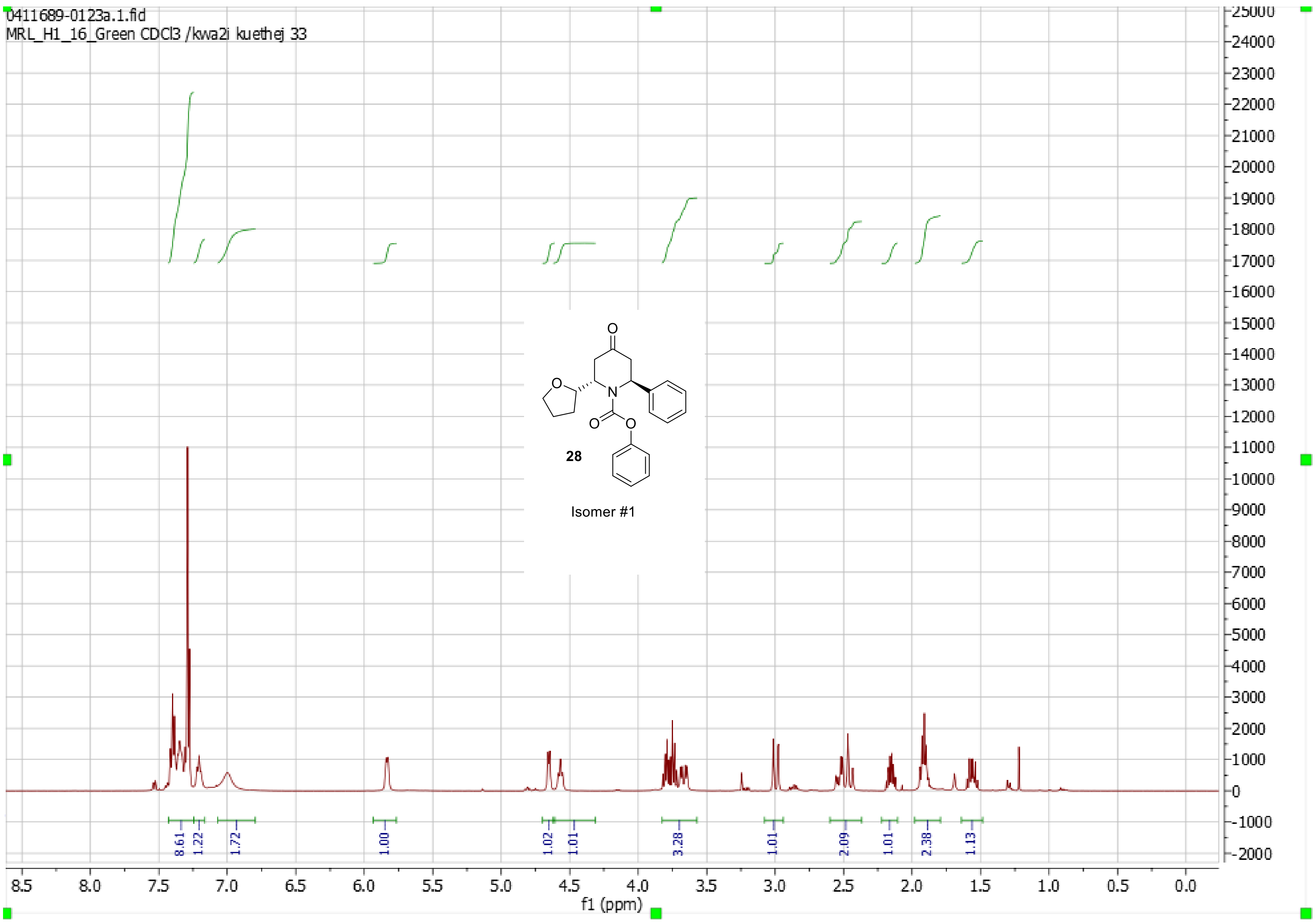
0411689-0123a.1.fid
MRL_H1_16_Green CDCl3 /kwa2i kuethej 33
28
Isomer #1
8.61
1.22
1.72
1.00
1.02
1.01
3.28
1.01
2.09
1.01
2.38
1.13
f1 (ppm)

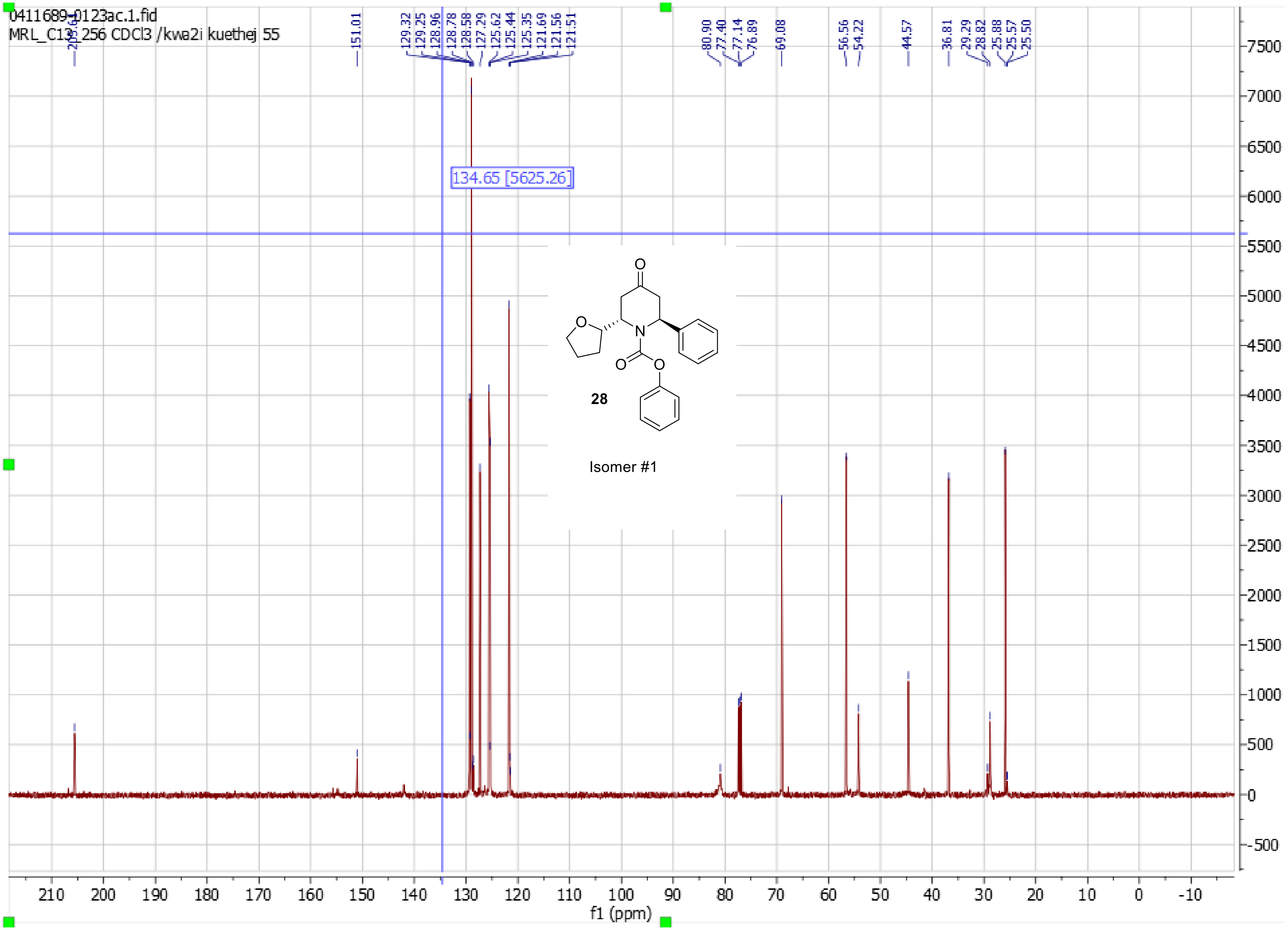

0411689-0123ac.1.fid
MRL_C13_256 CDCl3 /kwa2i kuethej 55
151.01
129.32
129.25
128.96
128.78
128.58
127.29
125.62
125.44
125.35
121.69
121.56
121.51
80.90
77.40
77.14
76.89
69.08
56.56
54.22
44.57
36.81
29.29
28.82
25.88
25.57
25.50
134.65 [5625.26]
28
Isomer #1
f1 (ppm)

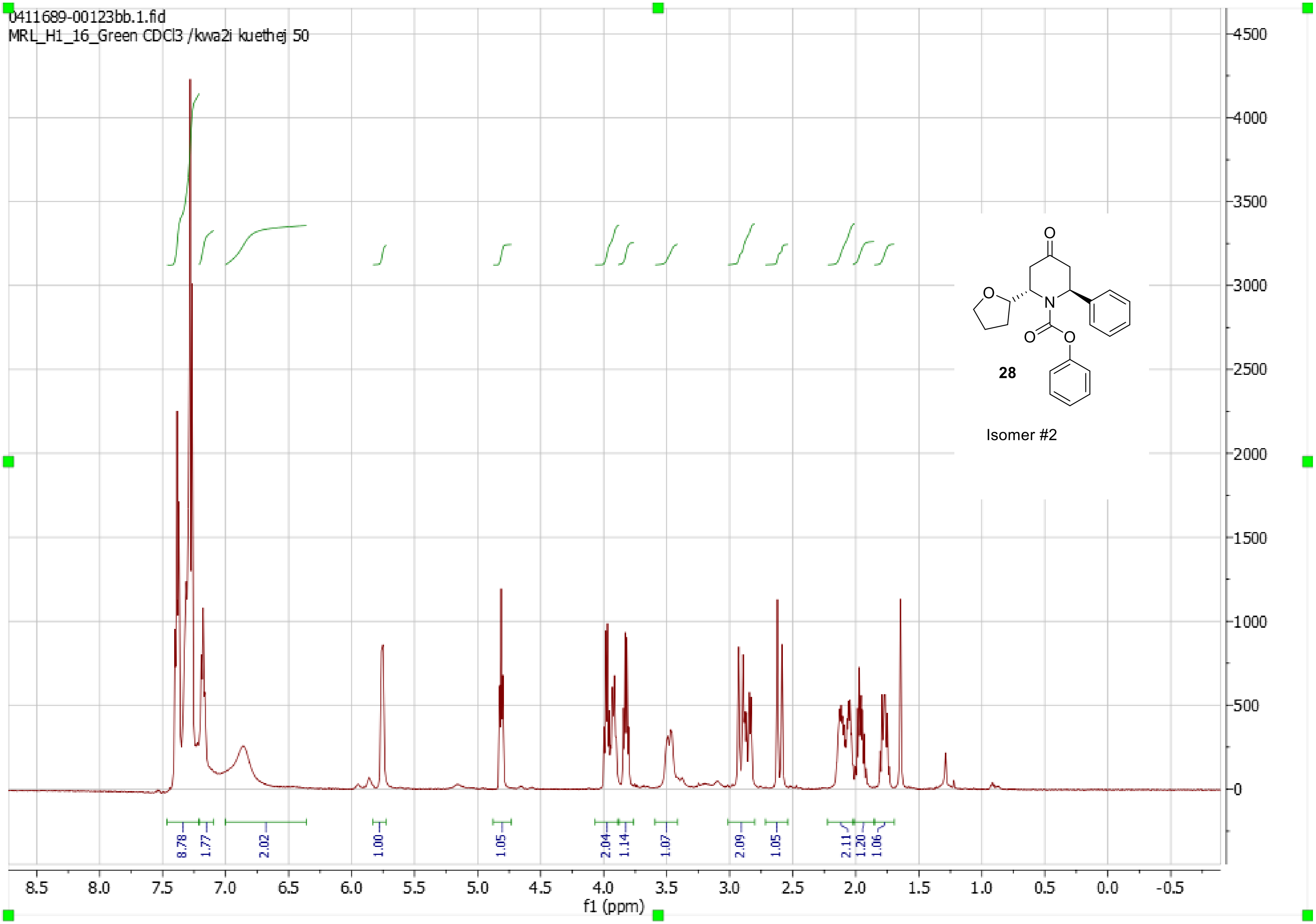

0411689-00123bb.1.fid
MRL_H1_16_Green CDCl3 /kwa2i kuethej 50
28
Isomer #2
8.78
1.77
2.02
1.00
1.05
2.04
1.14
1.07
2.09
1.05
2.11
1.20
1.06
f1 (ppm)

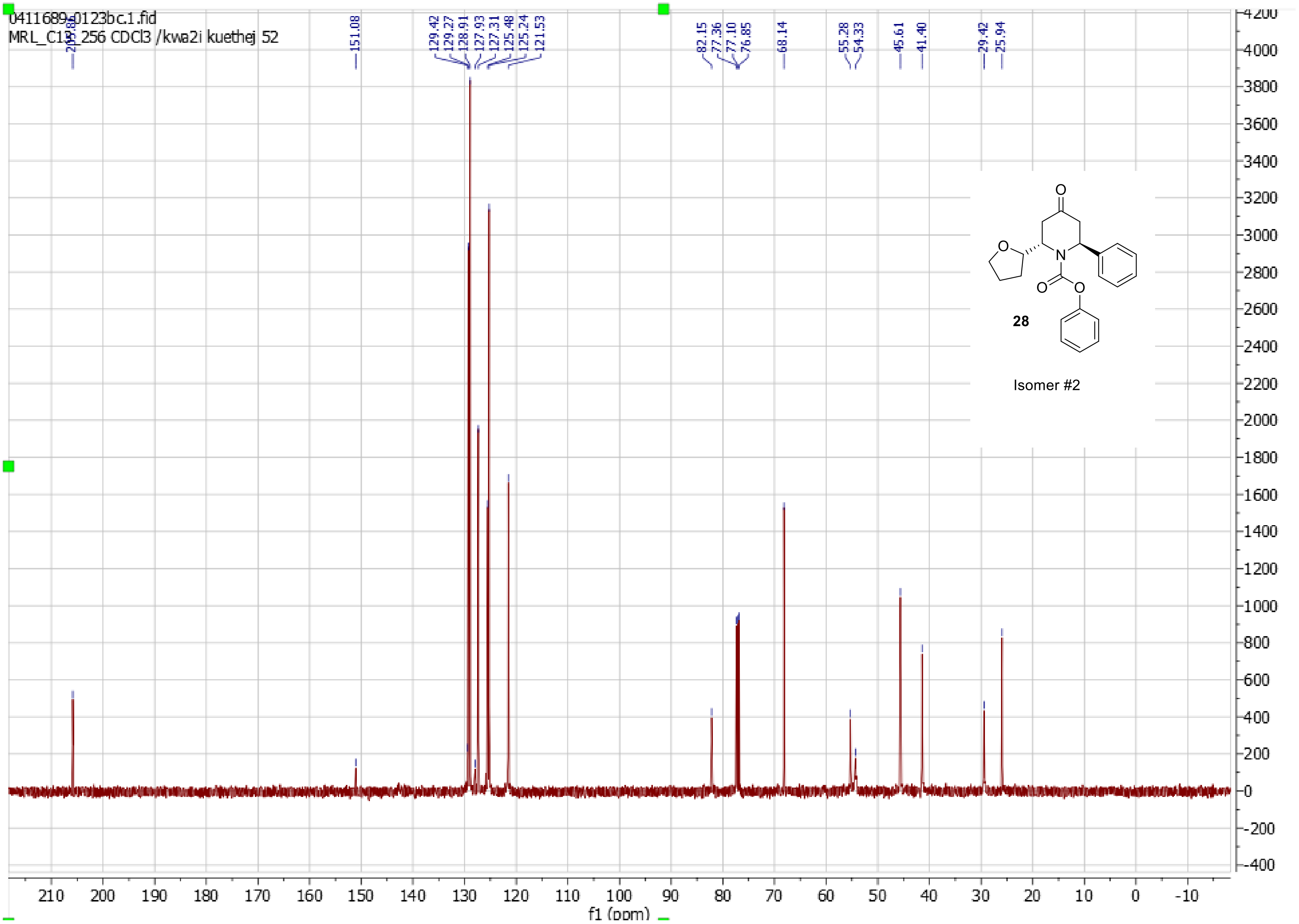
0411689-0123bc.1.fid
MRL_C13_256 CDCl3 /kwa2i kuethej 52
205.80
151.08
129.42
129.27
128.91
127.93
127.31
125.48
125.24
121.53
82.15
77.36
77.10
76.85
68.14
55.28
54.33
45.61
41.40
29.42
25.94
28
Isomer #2
f1 (ppm)

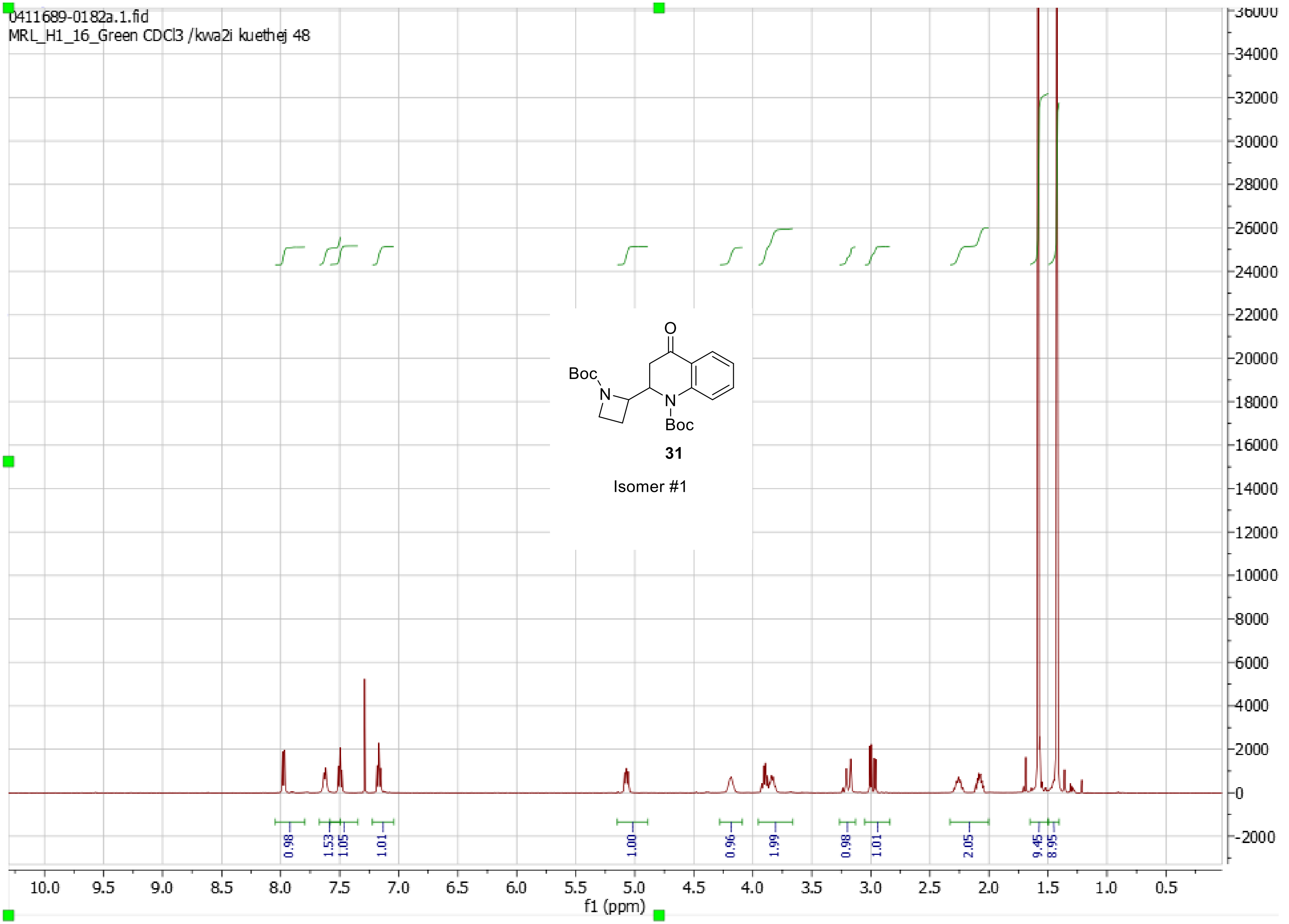
0411689-0182a.1.fid
MRL_H1_16_Green CDCl3 /kwa2i kuethej 48
Boc
N
O
N
Boc
31
Isomer #1
0.98
1.53
1.05
1.01
1.00
0.96
1.99
0.98
1.01
2.05
9.45
8.95
10.0
9.5
9.0
8.5
8.0
7.5
7.0
6.5
6.0
5.5
5.0
4.5
4.0
3.5
3.0
2.5
2.0
1.5
1.0
0.5
f1 (ppm)
36000
34000
32000
30000
28000
26000
24000
22000
20000
18000
16000
14000
12000
10000
8000
6000
4000
2000
0
-2000

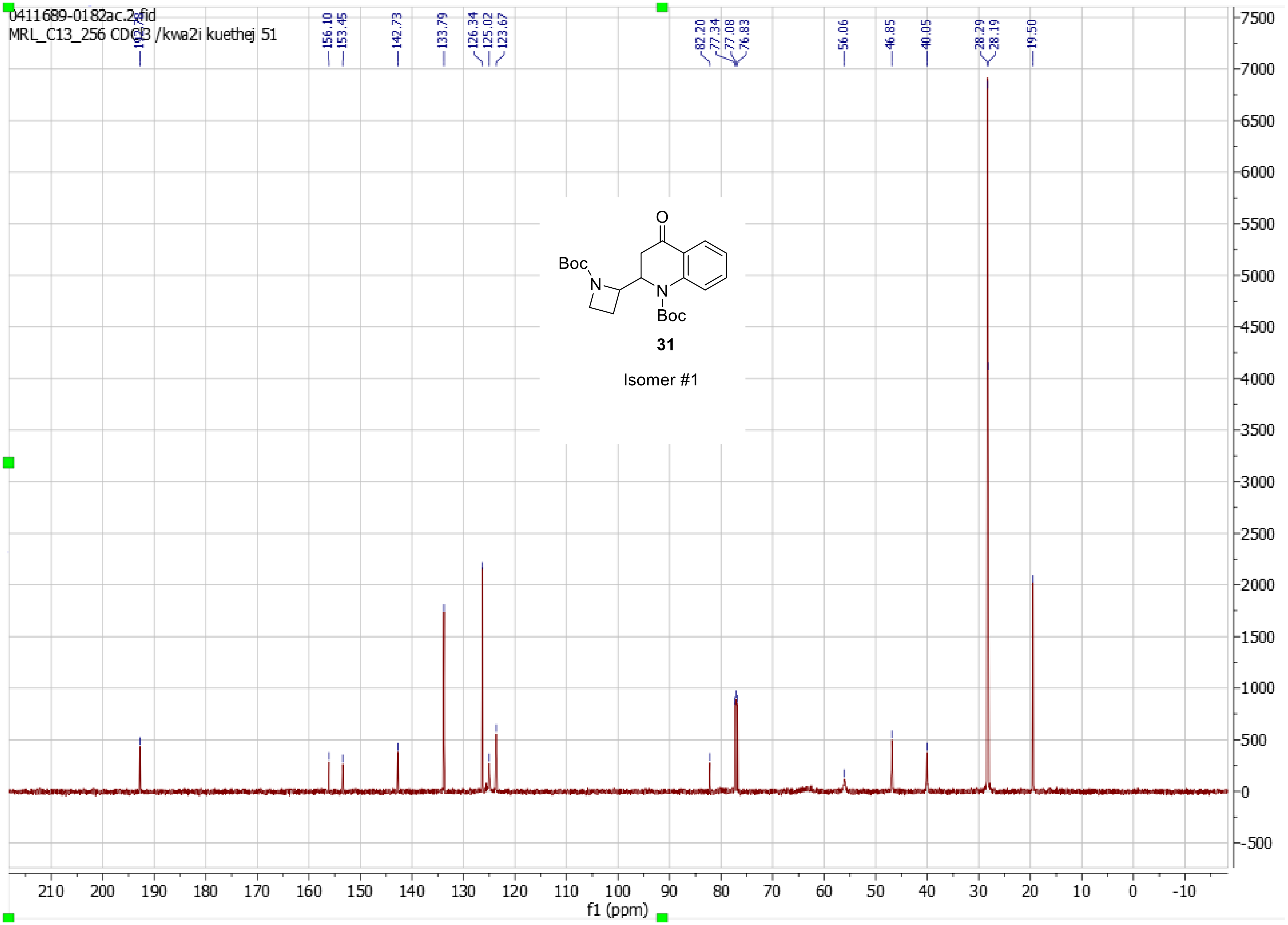
0411689-0182ac.2.fid
MRL_C13_256 CDCl3 /kwa2i kuethej 51
156.10
153.45
142.73
133.79
126.34
125.02
123.67
82.20
77.34
77.08
76.83
56.06
46.85
40.05
28.29
28.19
19.50
Boc
N
O
N
Boc
31
Isomer #1
f1 (ppm)

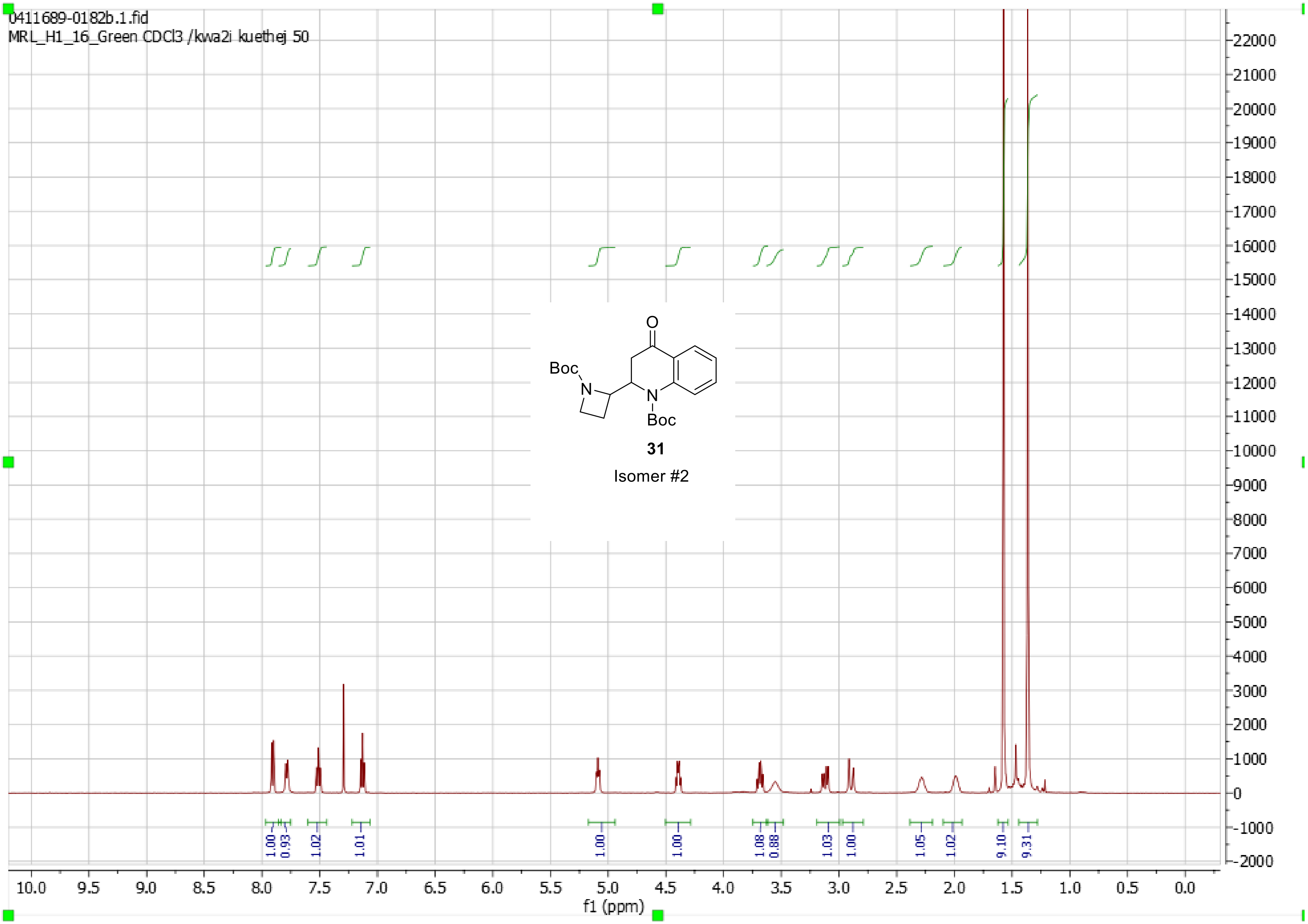

0411689-0182b.1.fid
MRL_H1_16_Green CDCl3 /kwa2i kuethej 50
Boc
N
O
N
Boc
31
Isomer #2
1.00
0.93
1.02
1.01
1.00
1.00
1.08
0.88
1.03
1.00
1.05
1.02
9.10
9.31
10.0 9.5 9.0 8.5 8.0 7.5 7.0 6.5 6.0 5.5 5.0 4.5 4.0 3.5 3.0 2.5 2.0 1.5 1.0 0.5 0.0
f1 (ppm)
22000 21000 20000 19000 18000 17000 16000 15000 14000 13000 12000 11000 10000 9000 8000 7000 6000 5000 4000 3000 2000 1000 0 -1000 -2000

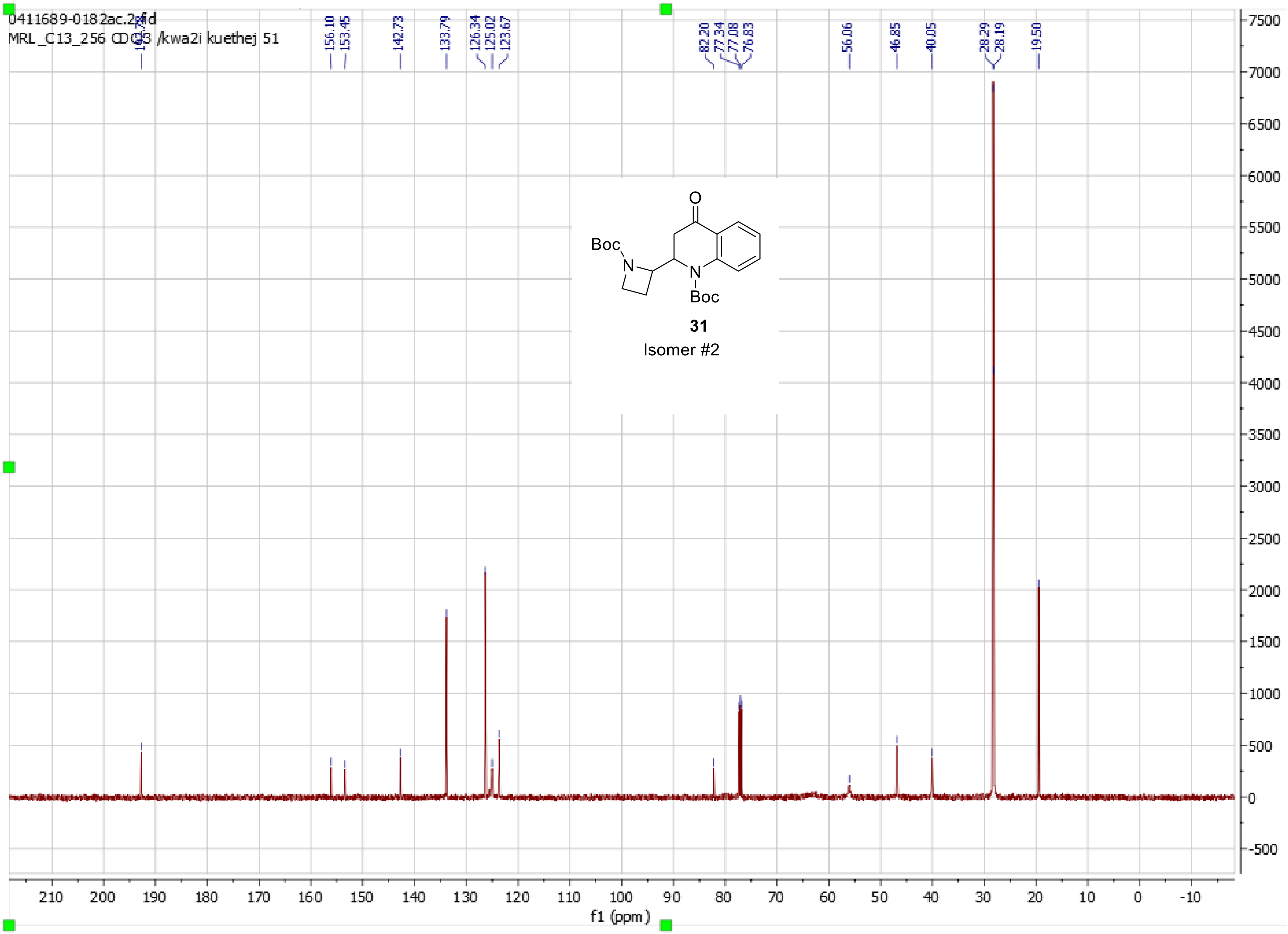
0411689-0182ac.2.fid
MRL_C13_256 CDCl3 /kwa2i kuethej 51
156.10
153.45
142.73
133.79
126.34
125.02
123.67
82.20
77.34
77.08
76.83
56.06
46.85
40.05
28.29
28.19
19.50
Boc
N
O
N
Boc
31
Isomer #2
f1 (ppm)

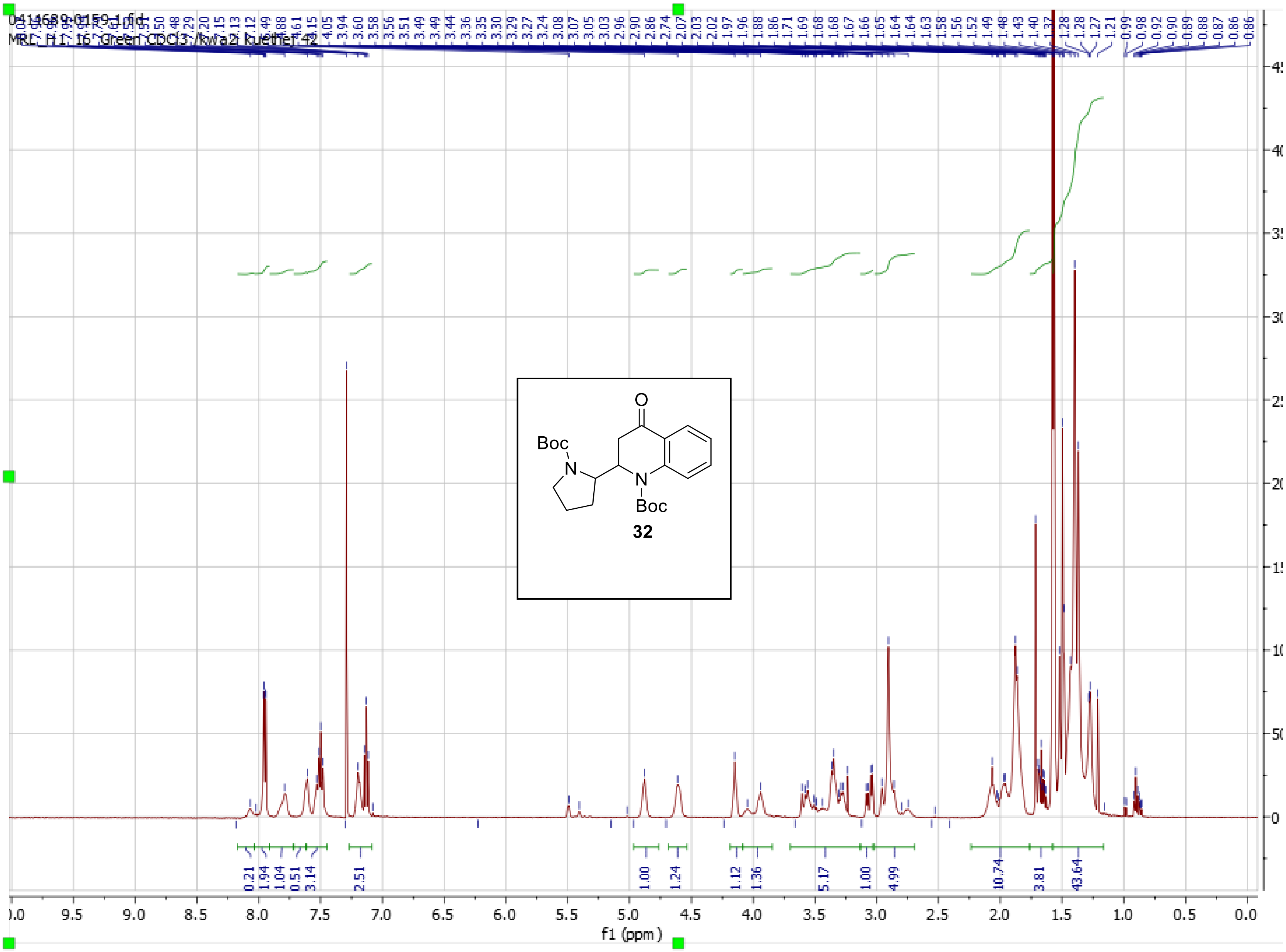

O
Boc
N
N
Boc
32
f1 (ppm)

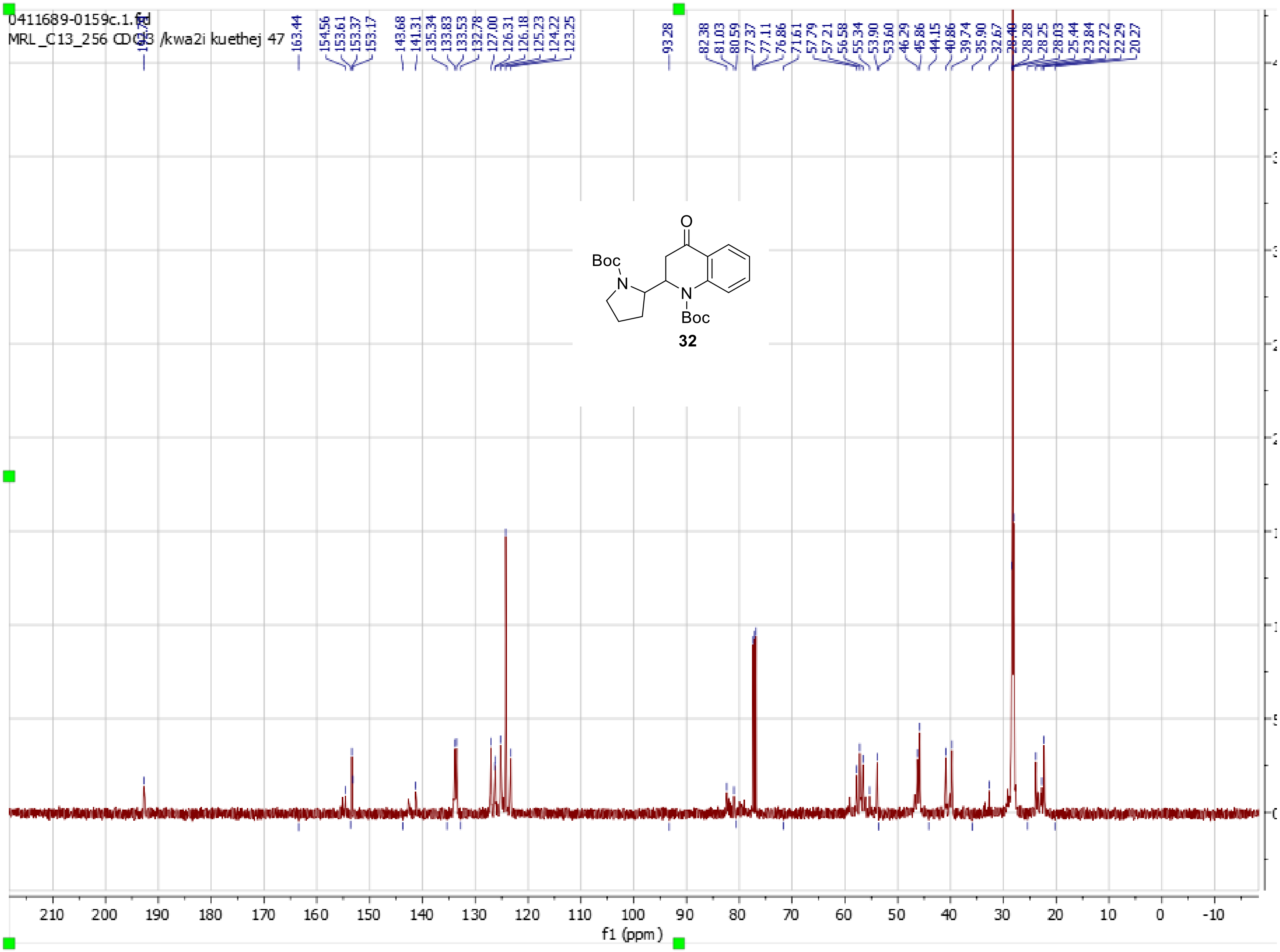

0411689-0159c.1.fid
MRL_C13_256 CDCl3 /kwa2i kuethej 47
163.44
154.56
153.61
153.37
153.17
143.68
141.31
135.34
133.83
133.53
132.78
127.00
126.31
126.18
125.23
124.22
123.25
93.28
82.38
81.03
80.59
77.37
77.11
76.86
71.61
57.79
57.21
56.58
55.34
53.90
53.60
46.29
45.86
44.15
40.86
39.74
35.90
32.67
28.40
28.28
28.25
28.03
25.44
23.84
22.72
22.29
20.27
O
Boc
N
N
Boc
32
210
200
190
180
170
160
150
140
130
120
110
100
90
80
70
60
50
40
30
20
10
0
-10
f1 (ppm)

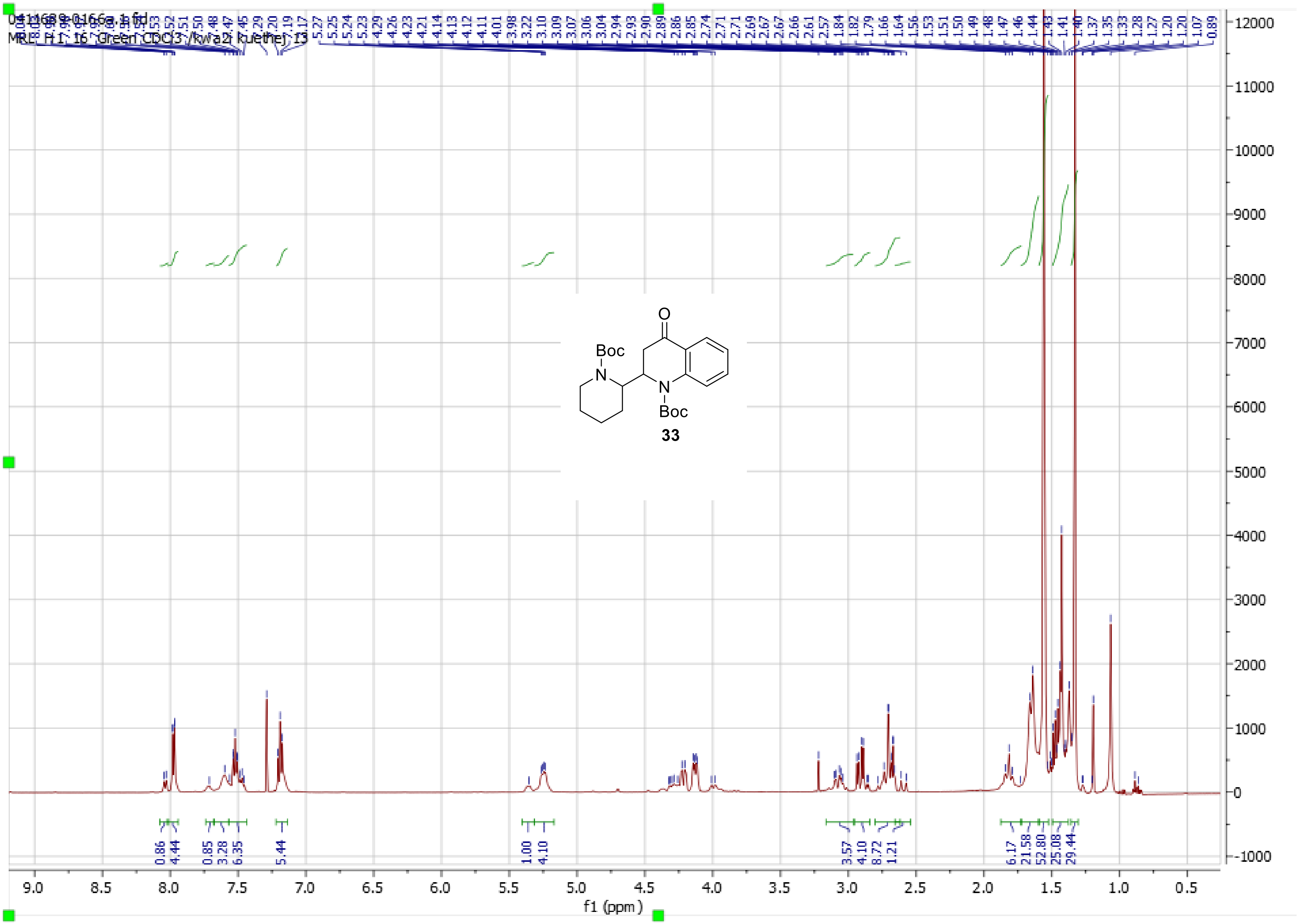

Boc
N
O
N
Boc
33
f1 (ppm)

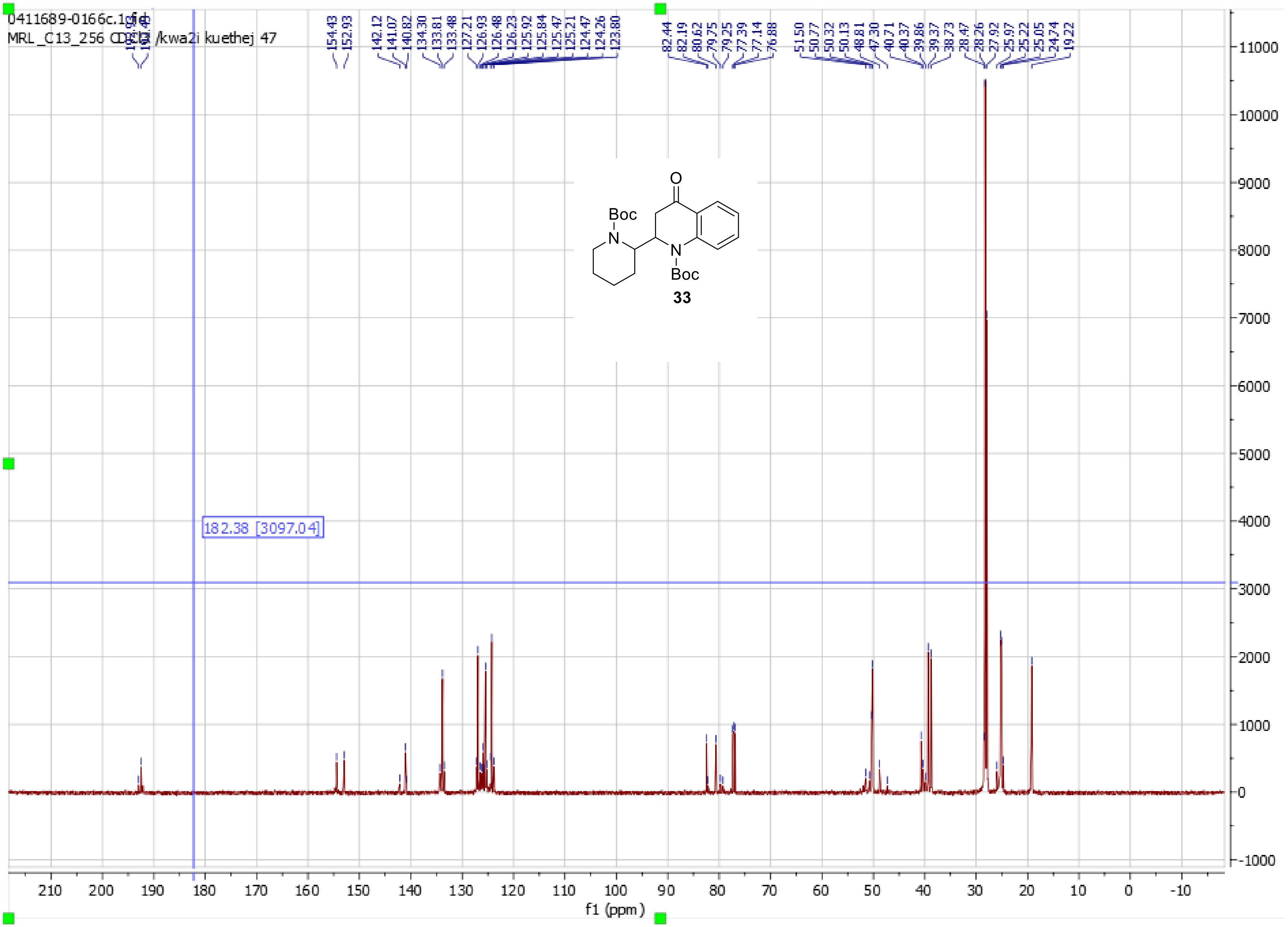
0411689-0166c.1.fid
MRL_C13_256 CDCl3 /kwa2i kuethej 47
154.43
152.93
142.12
141.07
140.82
134.30
133.81
133.48
127.21
126.93
126.48
126.23
125.92
125.84
125.47
125.21
124.47
124.26
123.80
82.44
82.19
80.62
79.75
79.25
77.39
77.14
76.88
51.50
50.77
50.32
50.13
48.81
47.30
40.71
40.37
39.86
39.37
38.73
28.47
28.26
27.92
25.97
25.22
25.05
24.74
19.22
Boc
N
O
Boc
33
182.38 [3097.04]
f1 (ppm)

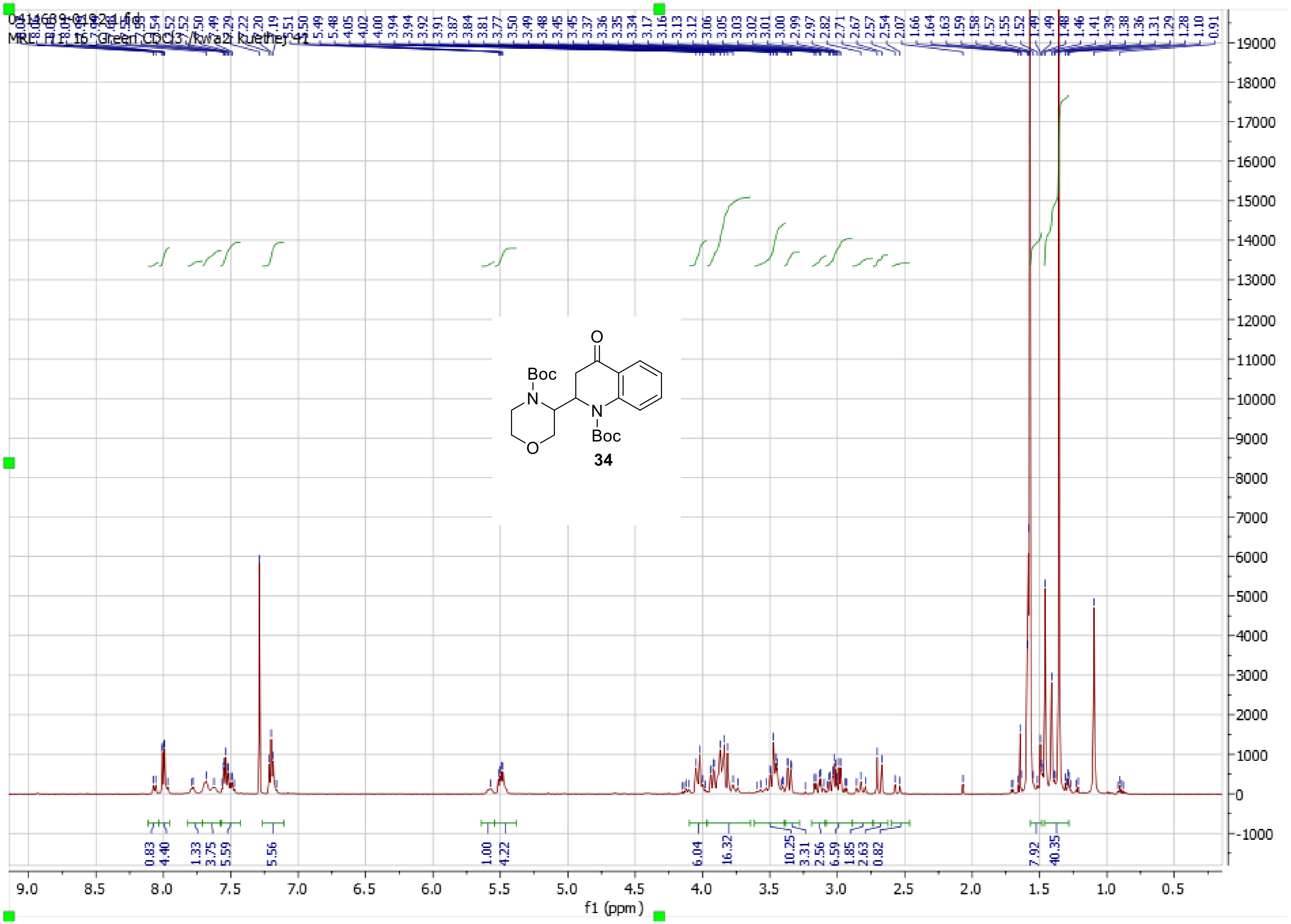

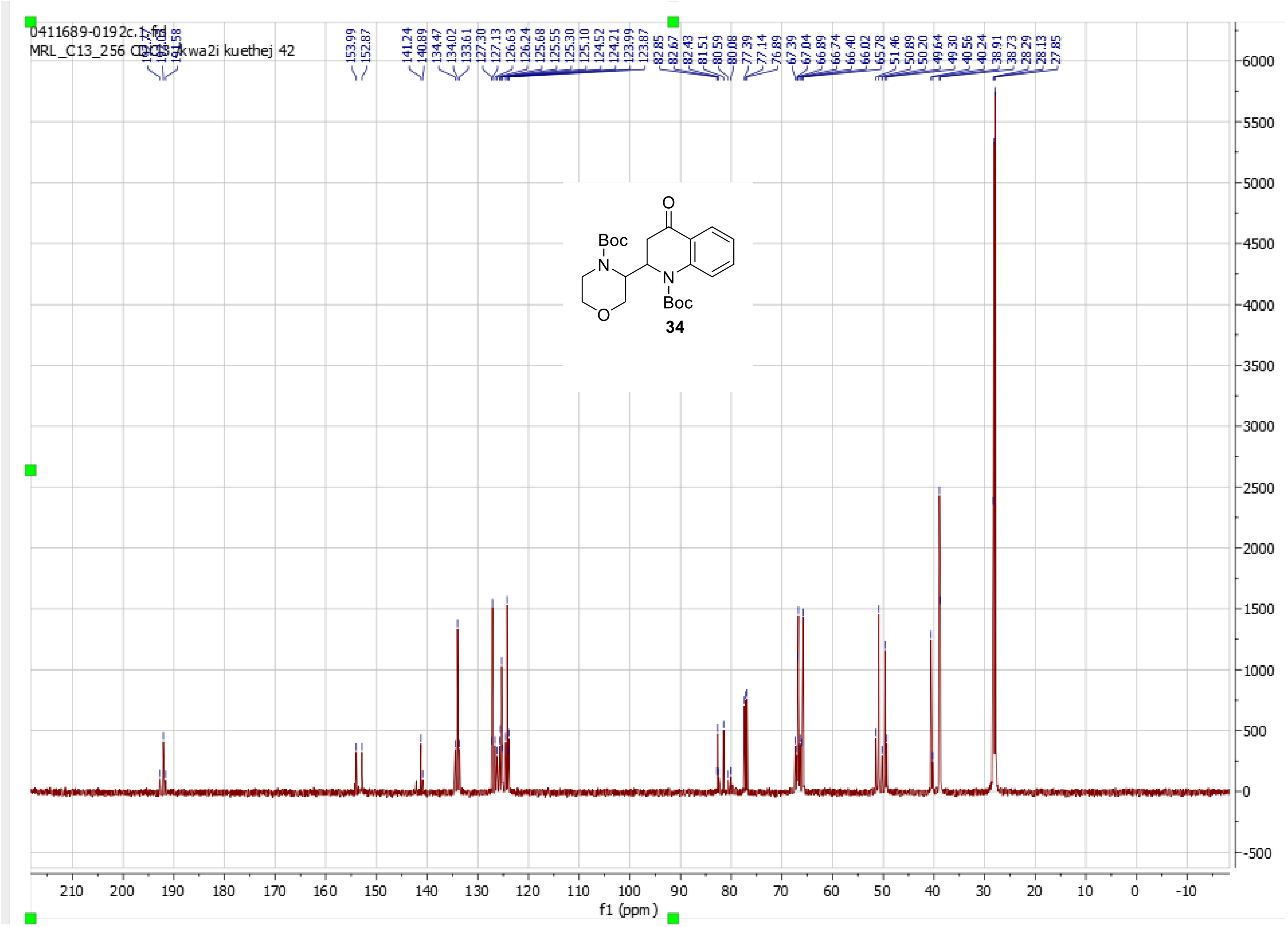

0411689-0192c.1.fid
MRL_C13_256 CDCl3 kwa2i kuethej 42
191.58
153.99
152.87
141.24
140.89
134.47
134.02
133.61
127.30
127.13
126.63
126.24
125.68
125.55
125.30
125.10
124.52
124.21
123.99
123.87
82.85
82.67
82.43
81.51
80.59
80.08
77.39
77.14
76.89
67.39
67.04
66.89
66.74
66.40
66.02
65.78
51.46
50.89
50.20
49.64
49.30
40.56
40.24
38.91
38.73
28.29
28.13
27.85
Boc
N
O
O
N
Boc
34
f1 (ppm)

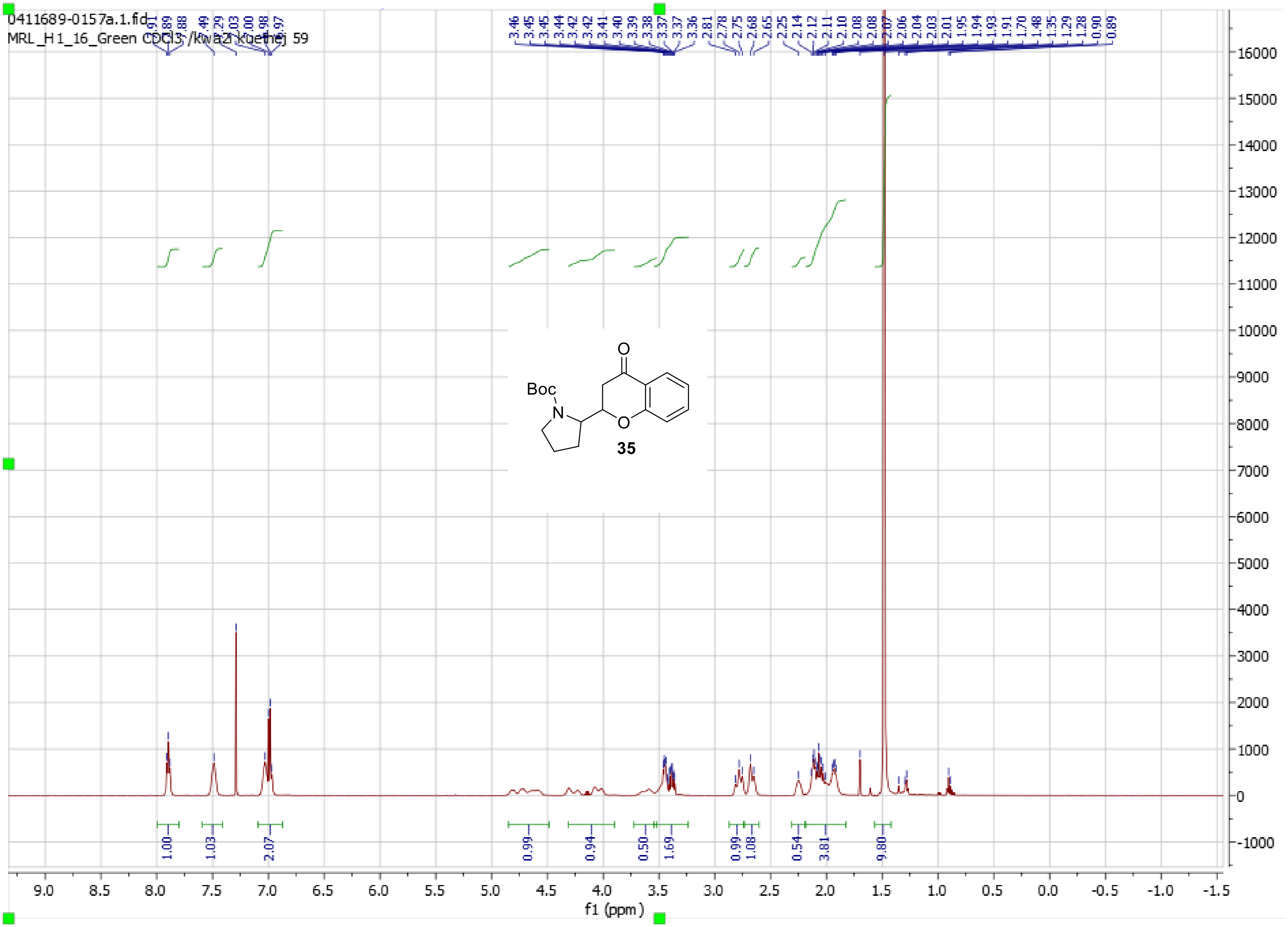
0411689-0157a.1.fid
MRL_H1_16_Green CDCl3 /kwa2l kuethej 59
7.91 7.89 7.88 7.49 7.29 7.03 7.00 6.98 6.97
3.46 3.45 3.45 3.44 3.42 3.42 3.41 3.40 3.39 3.38 3.37 3.37 3.36 2.81 2.78 2.75 2.68 2.65 2.25 2.14 2.12 2.11 2.10 2.08 2.08 2.07 2.06 2.04 2.03 2.01 1.95 1.94 1.93 1.91 1.70 1.48 1.35 1.29 1.28 0.90 0.89
1.00 1.03 2.07 0.99 0.94 0.50 1.69 0.99 1.08 0.54 3.81 9.80
Boc
N
O
O
35
f1 (ppm)

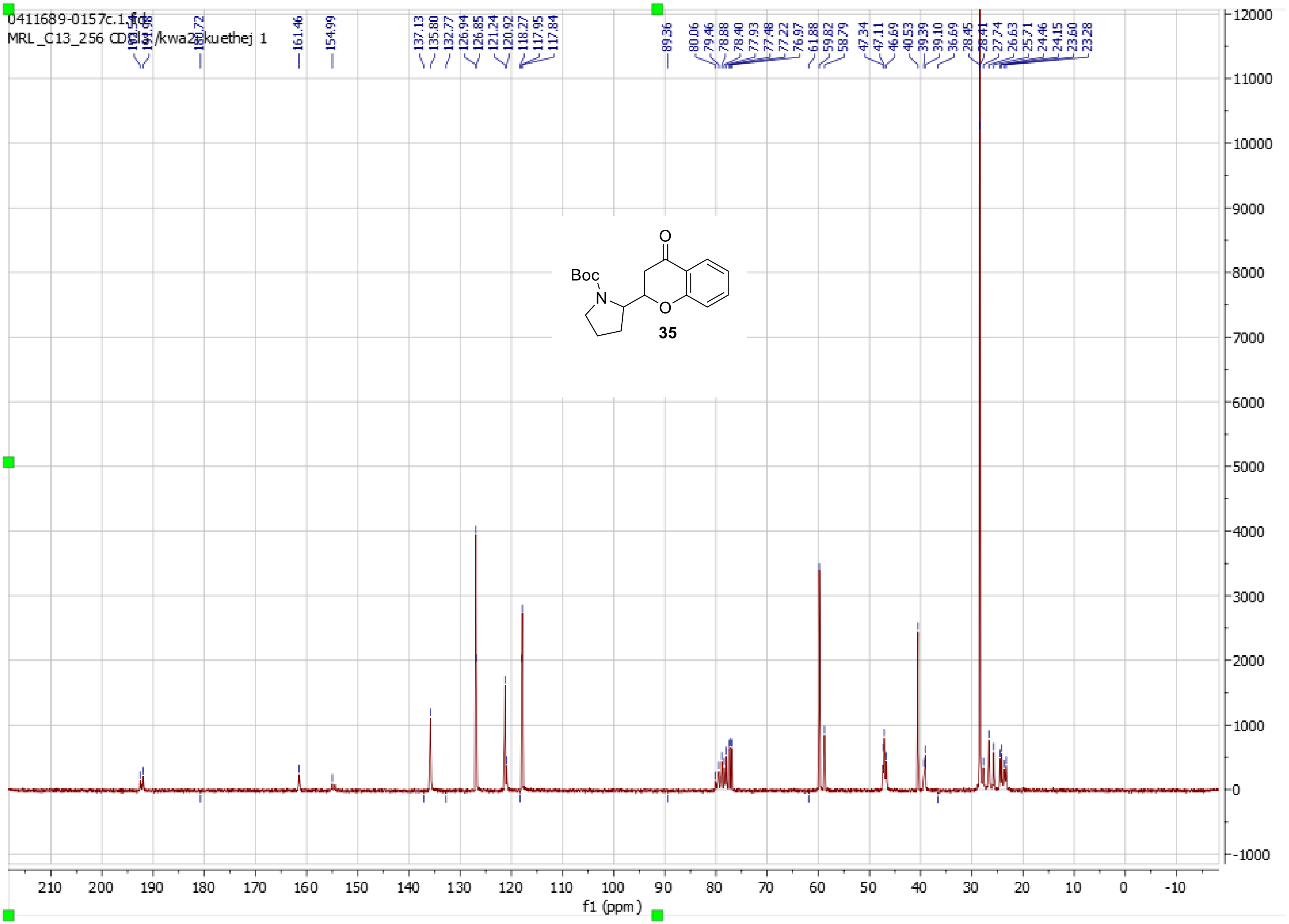
0411689-0157c.1.fid
MRL_C13_256 CDCl3 /kwa2/kuethej 1
180.72
161.46
154.99
137.13
135.80
132.77
126.94
126.85
121.24
120.92
118.27
117.95
117.84
89.36
80.06
79.46
78.88
78.40
77.93
77.48
77.22
76.97
61.88
59.82
58.79
47.34
47.11
46.69
40.53
39.39
39.10
36.69
28.45
28.41
27.74
26.63
25.71
24.46
24.15
23.60
23.28
Boc
N
O
O
35
f1 (ppm)

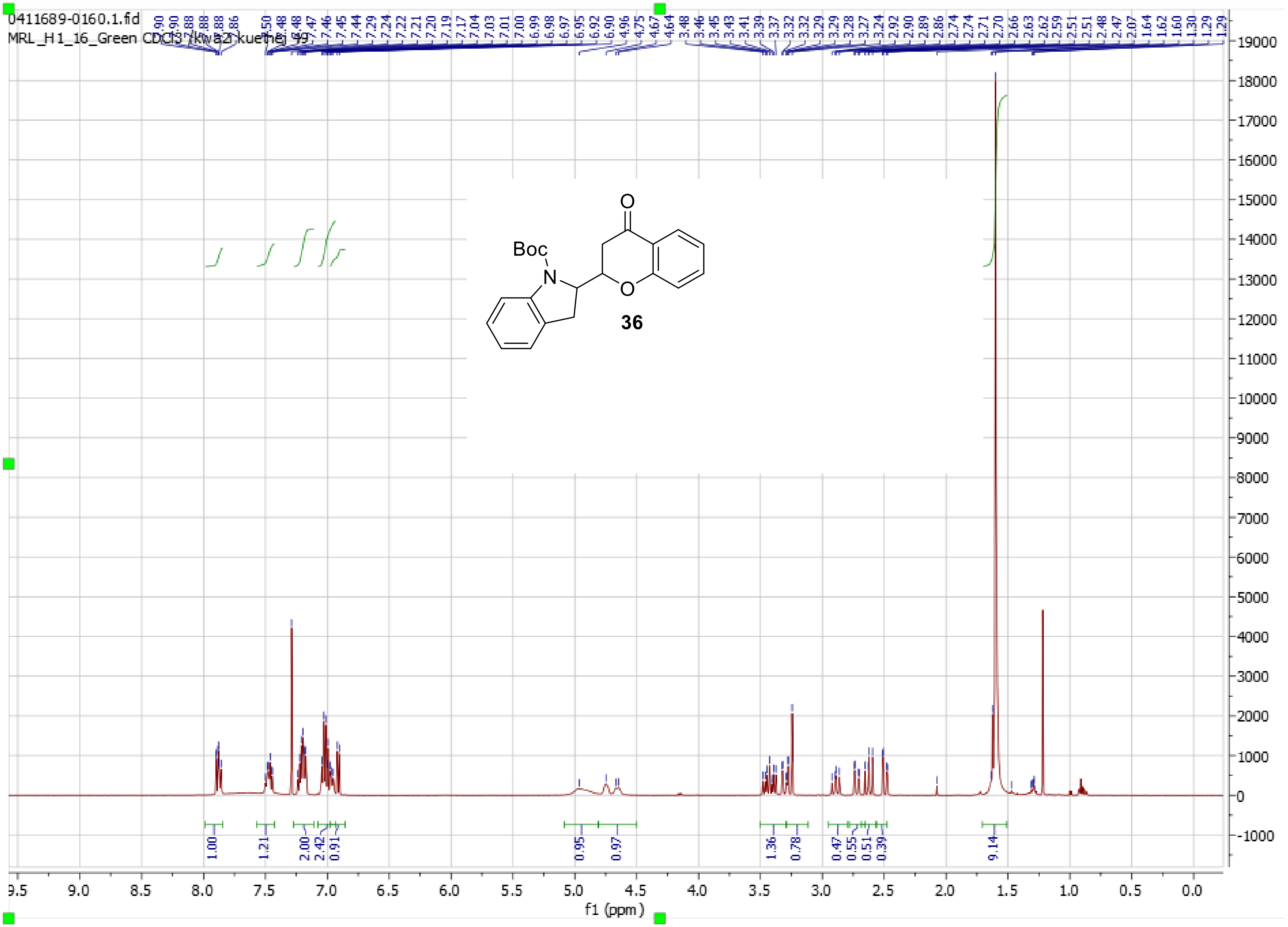
0411689-0160.1.fid
MRL_H1_16_Green CDCl3 (kwa2) kuethej 99
Boc
N
O
O
36
f1 (ppm)

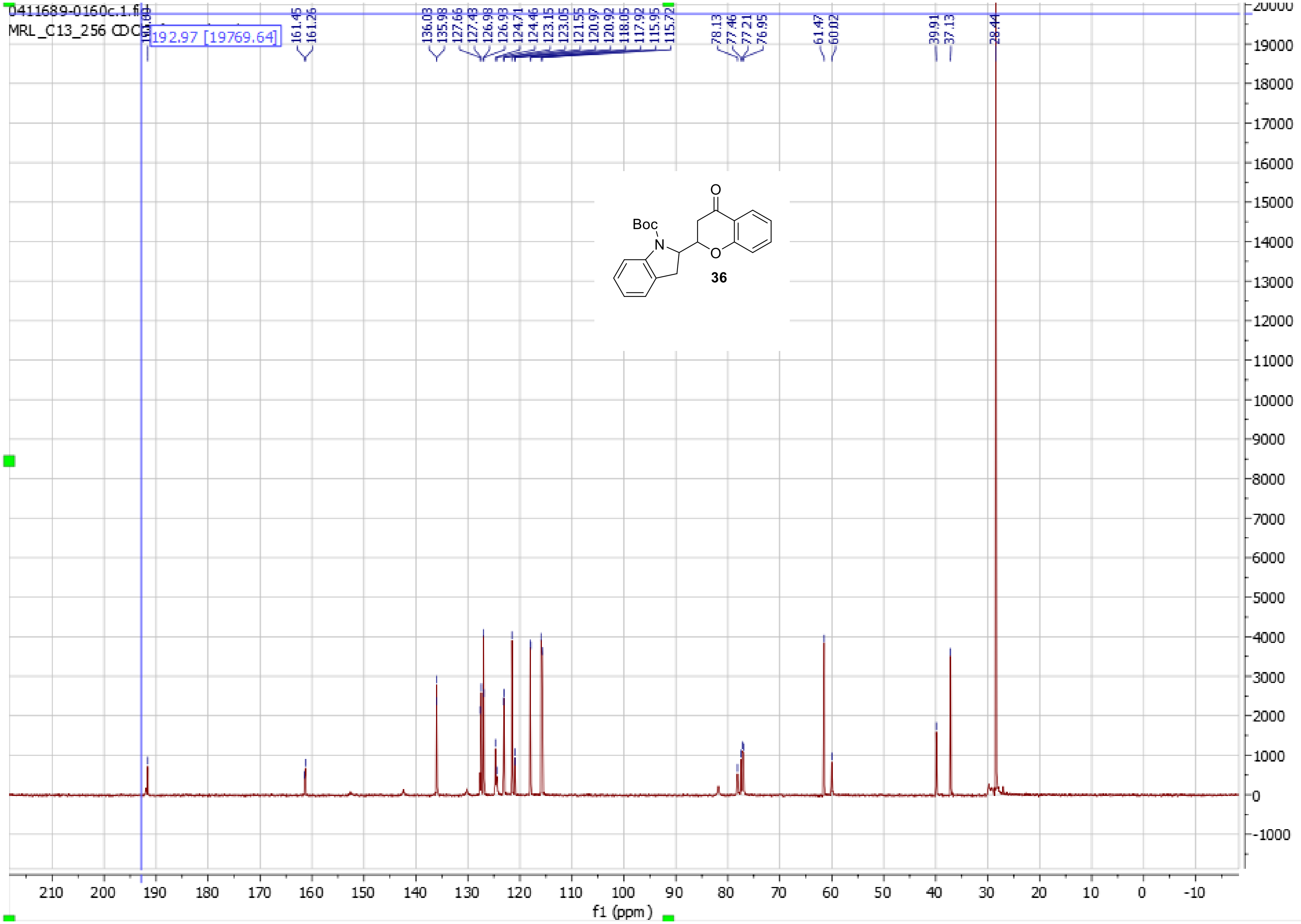
0411689-0160c.1.fid
MRL_C13_256 CDCl3
192.97 [19769.64]
161.45
161.26
136.03
135.98
127.66
127.43
126.98
126.93
124.71
124.46
123.15
123.05
121.55
120.97
120.92
118.05
117.92
115.95
115.72
78.13
77.46
77.21
76.95
61.47
60.02
39.91
37.13
28.44
Boc
N
O
O
36
f1 (ppm)

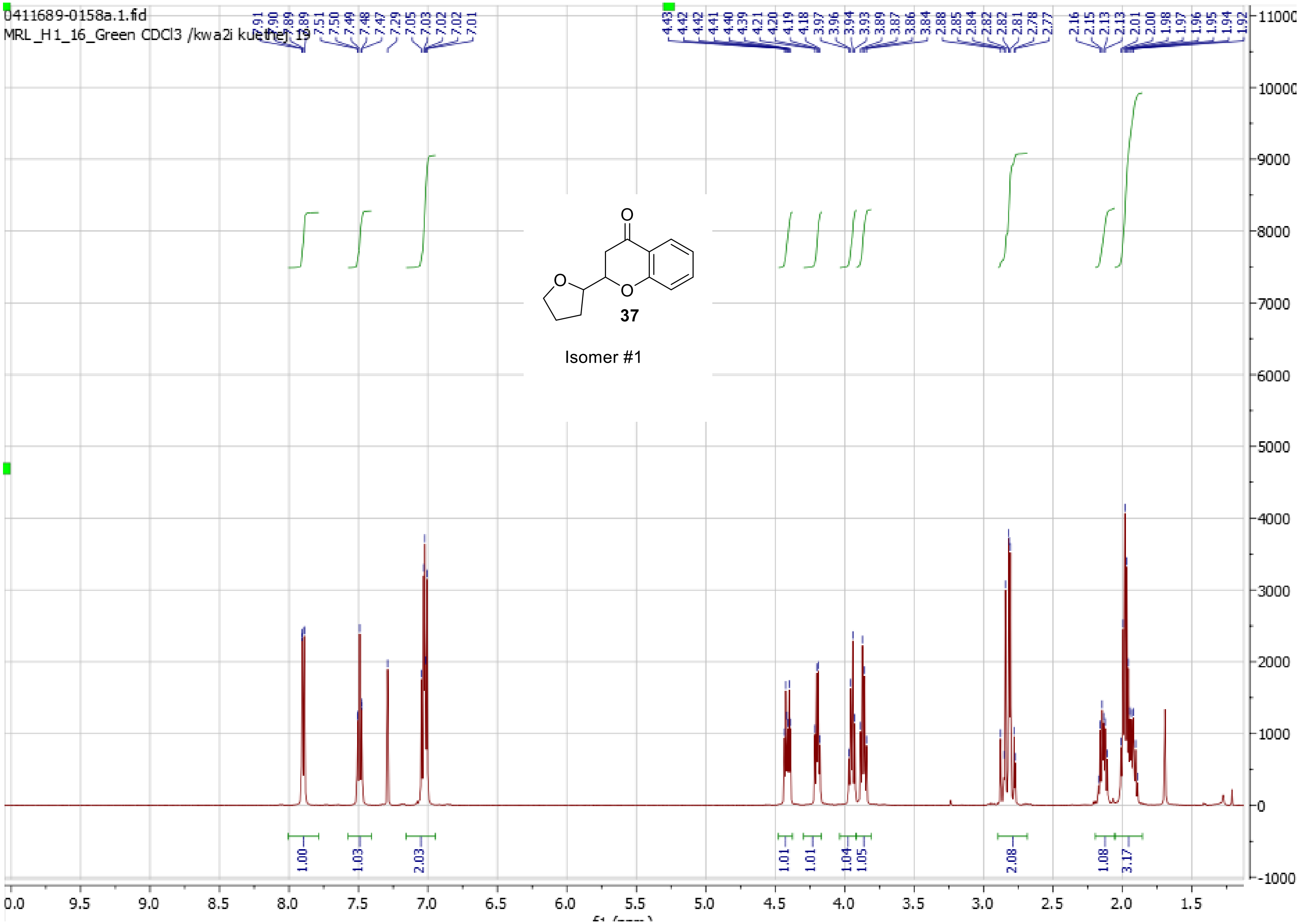
0411689-0158a.1.fid
MRL _H1_16_Green CDCl3 /kwa2i kuethe) 19
37
Isomer #1
1.00
1.03
2.03
1.01
1.01
1.04
1.05
2.08
1.08
3.17

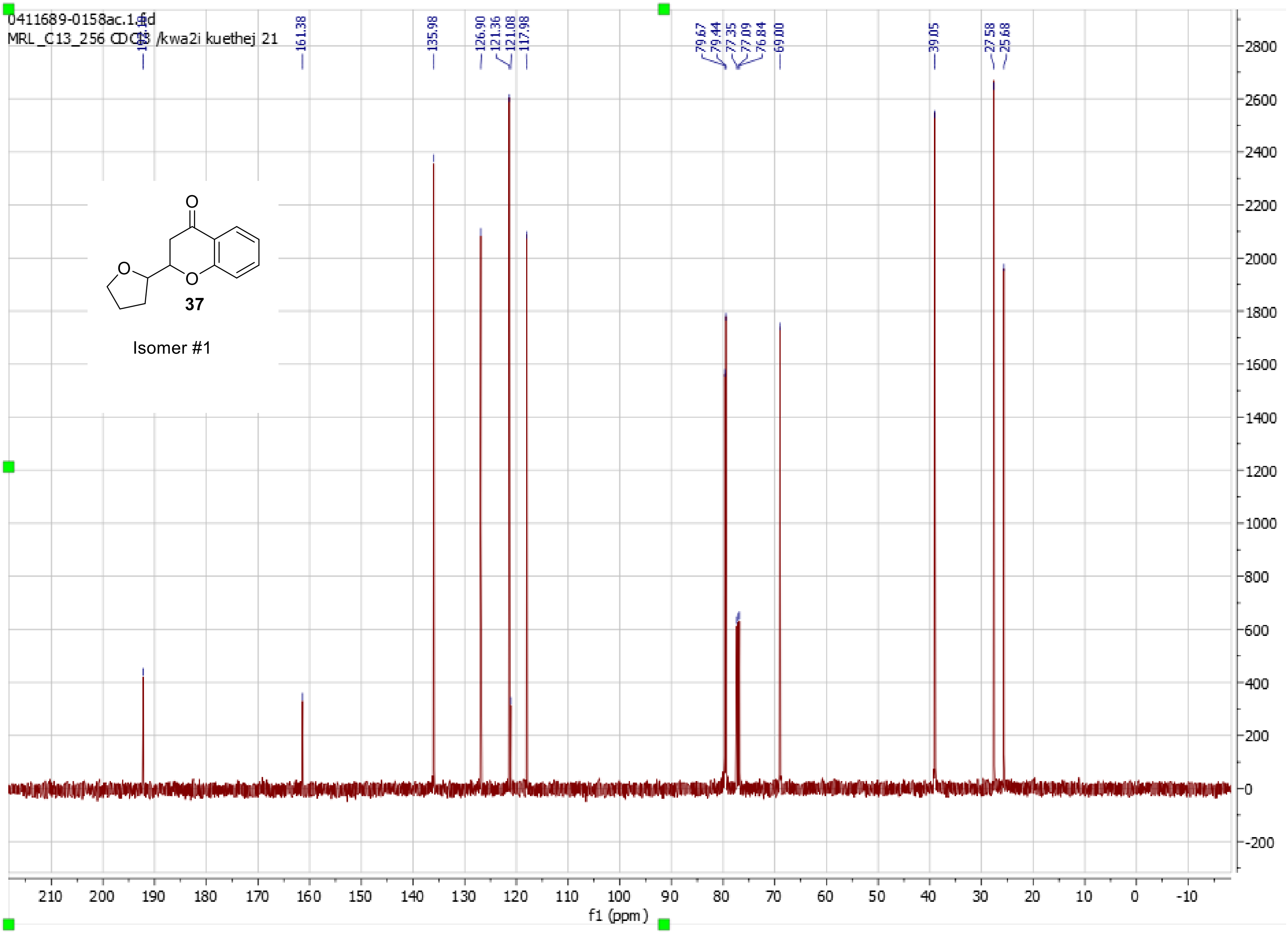

0411689-0158ac.1.fid
MRL_C13_256 CDCl3 /kwa2i kuethej 21
161.38
135.98
126.90
121.36
121.08
117.98
79.67
79.44
77.35
77.09
76.84
69.00
39.05
27.58
25.68
37
Isomer #1
f1 (ppm)

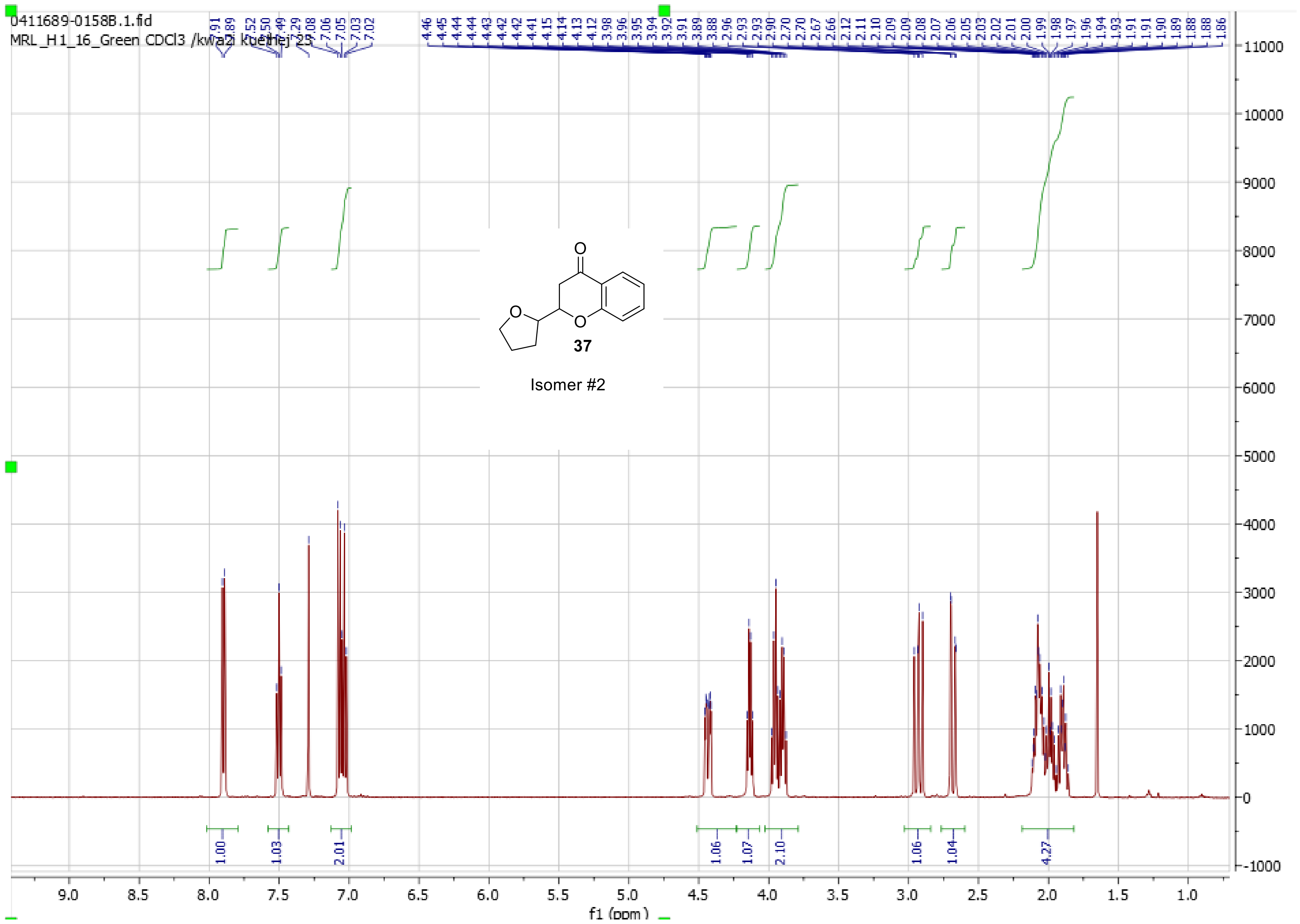

0411689-0158B.1.fid
MRL_H1_16_Green CDCl3 /kwazi Kuethej 23
37
Isomer #2
f1 (ppm)

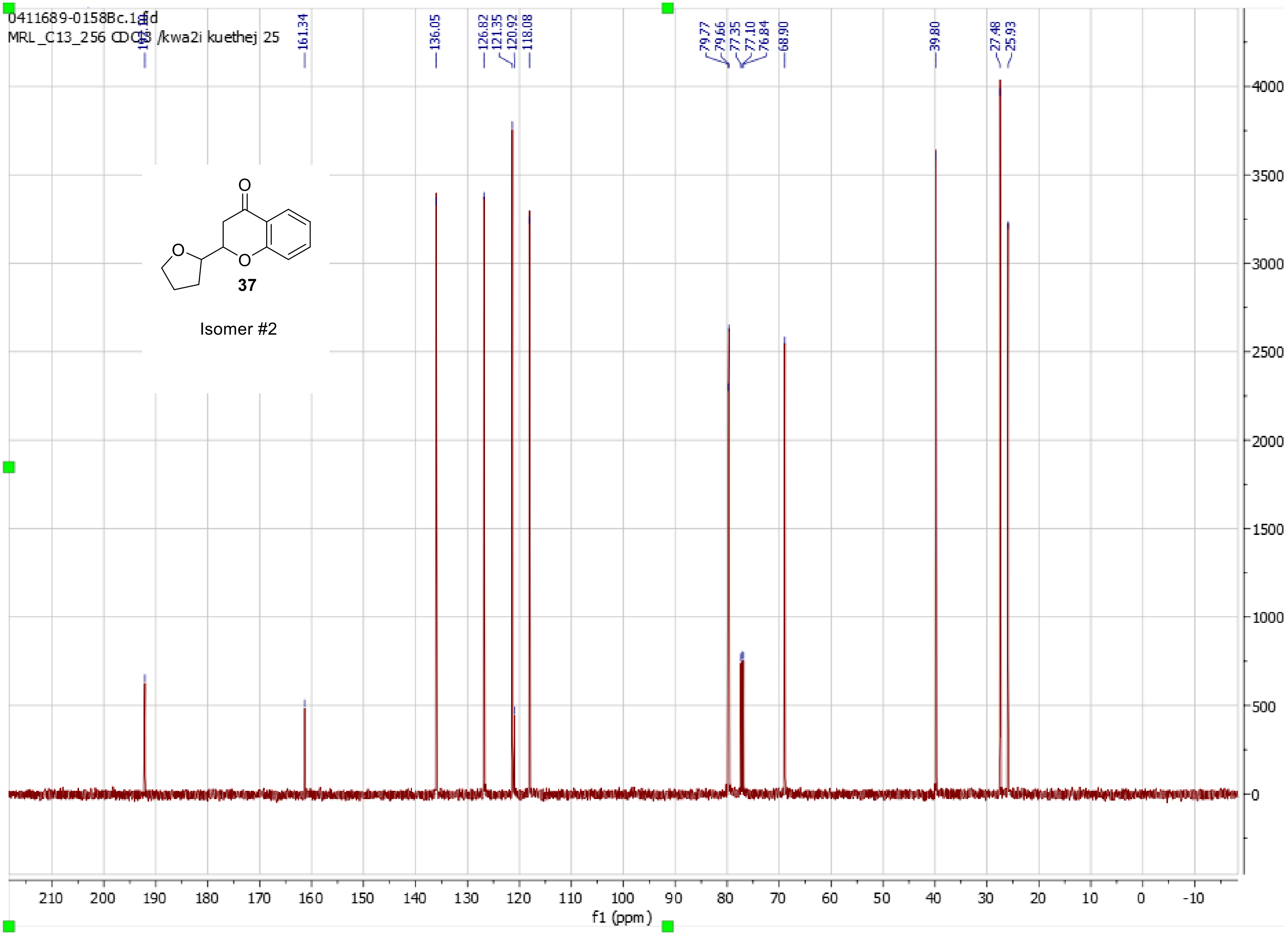
0411689-0158Bc.1.fid
MRL_C13_256 CDCl3 /kwa2i kuethej 25
161.34
136.05
126.82
121.35
120.92
118.08
79.77
79.66
77.35
77.10
76.84
68.90
39.80
27.48
25.93
O
O
O
37
Isomer #2
f1 (ppm)

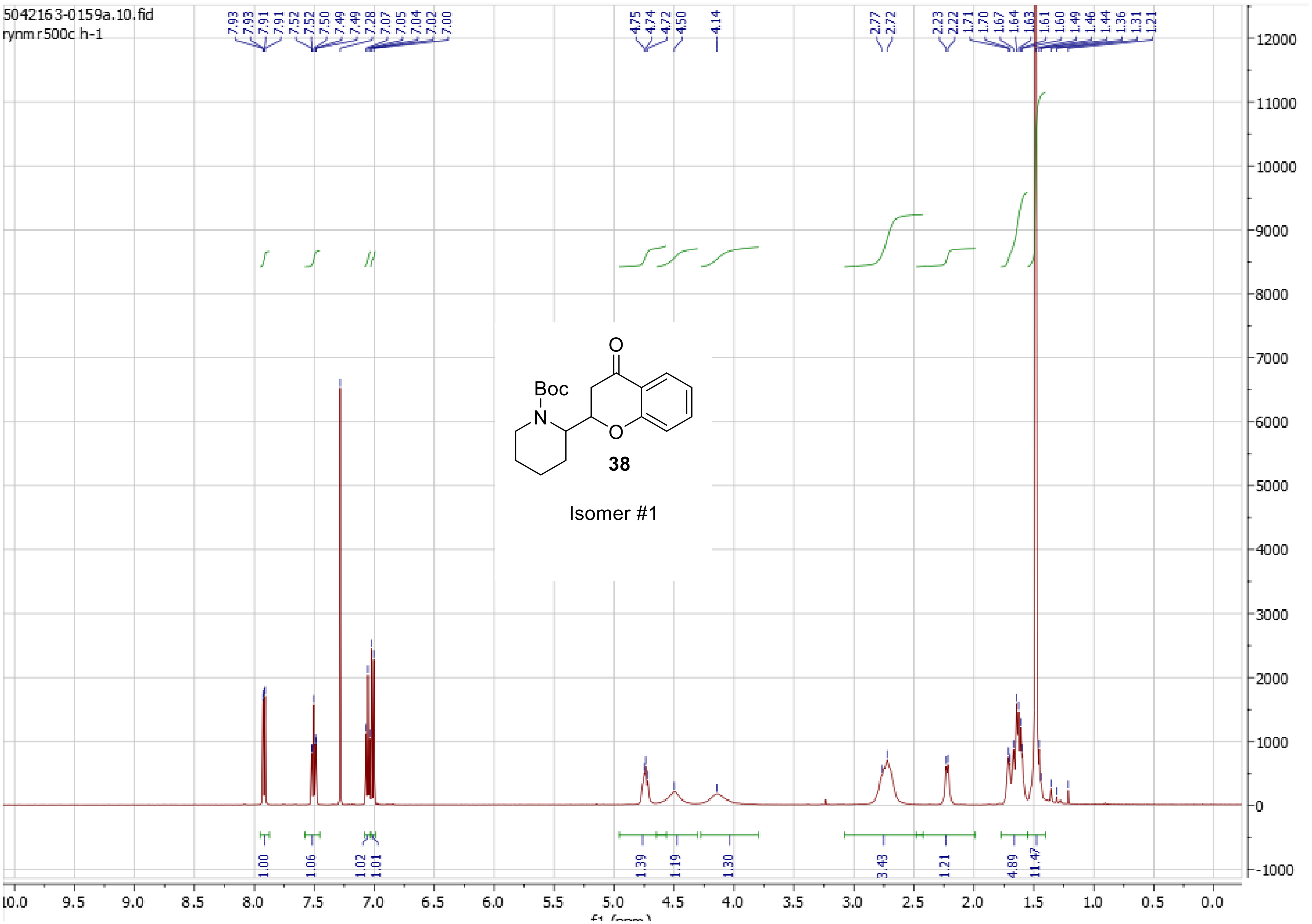

5042163-0159a.10.fid
rynmr500c h-1
7.93 7.93 7.91 7.91 7.52 7.52 7.50 7.49 7.49 7.28 7.07 7.05 7.04 7.02 7.00
4.75 4.74 4.72 4.50
4.14
2.77 2.72
2.23 2.22 1.71 1.70 1.67 1.64 1.63 1.61 1.60 1.49 1.46 1.44 1.36 1.31 1.21
Boc
N
O
O
38
Isomer #1
1.00 1.06 1.02 1.01 1.39 1.19 1.30 3.43 1.21 4.89 11.47
f1 (ppm)

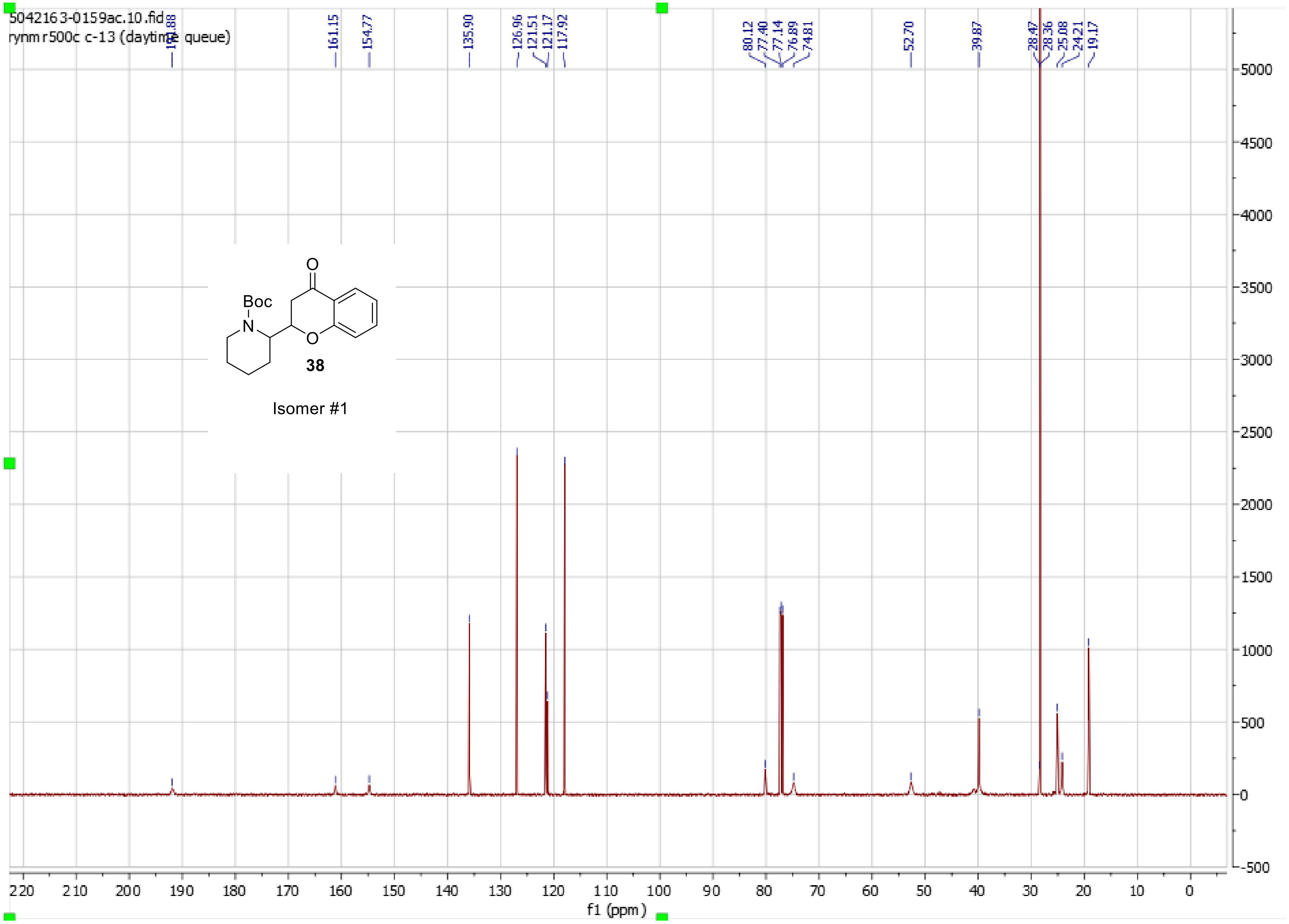

5042163-0159ac.10.fid
rynmr500c c-13 (daytime queue)
161.15
154.77
135.90
126.96
121.51
121.17
117.92
80.12
77.40
77.14
76.89
74.81
52.70
39.87
28.47
28.36
25.08
24.21
19.17
Boc
N
O
O
38
Isomer #1
f1 (ppm)

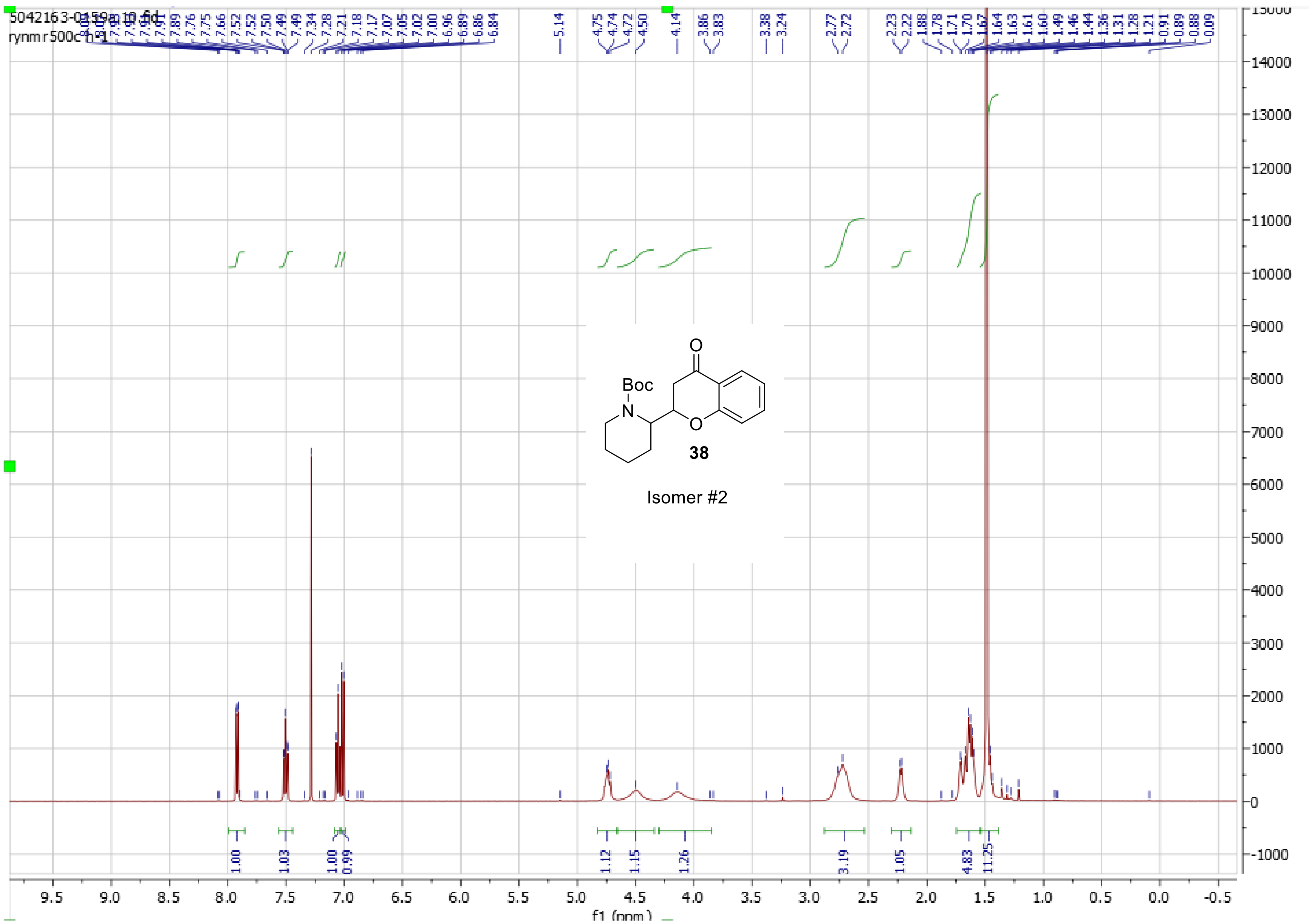

Boc
N
O
O
38
Isomer #2
1.00
1.03
1.00
0.99
1.12
1.15
1.26
3.19
1.05
4.83
11.25
9.5 9.0 8.5 8.0 7.5 7.0 6.5 6.0 5.5 5.0 4.5 4.0 3.5 3.0 2.5 2.0 1.5 1.0 0.5 0.0 -0.5
f1 (ppm)
14000 13000 12000 11000 10000 9000 8000 7000 6000 5000 4000 3000 2000 1000 0 -1000

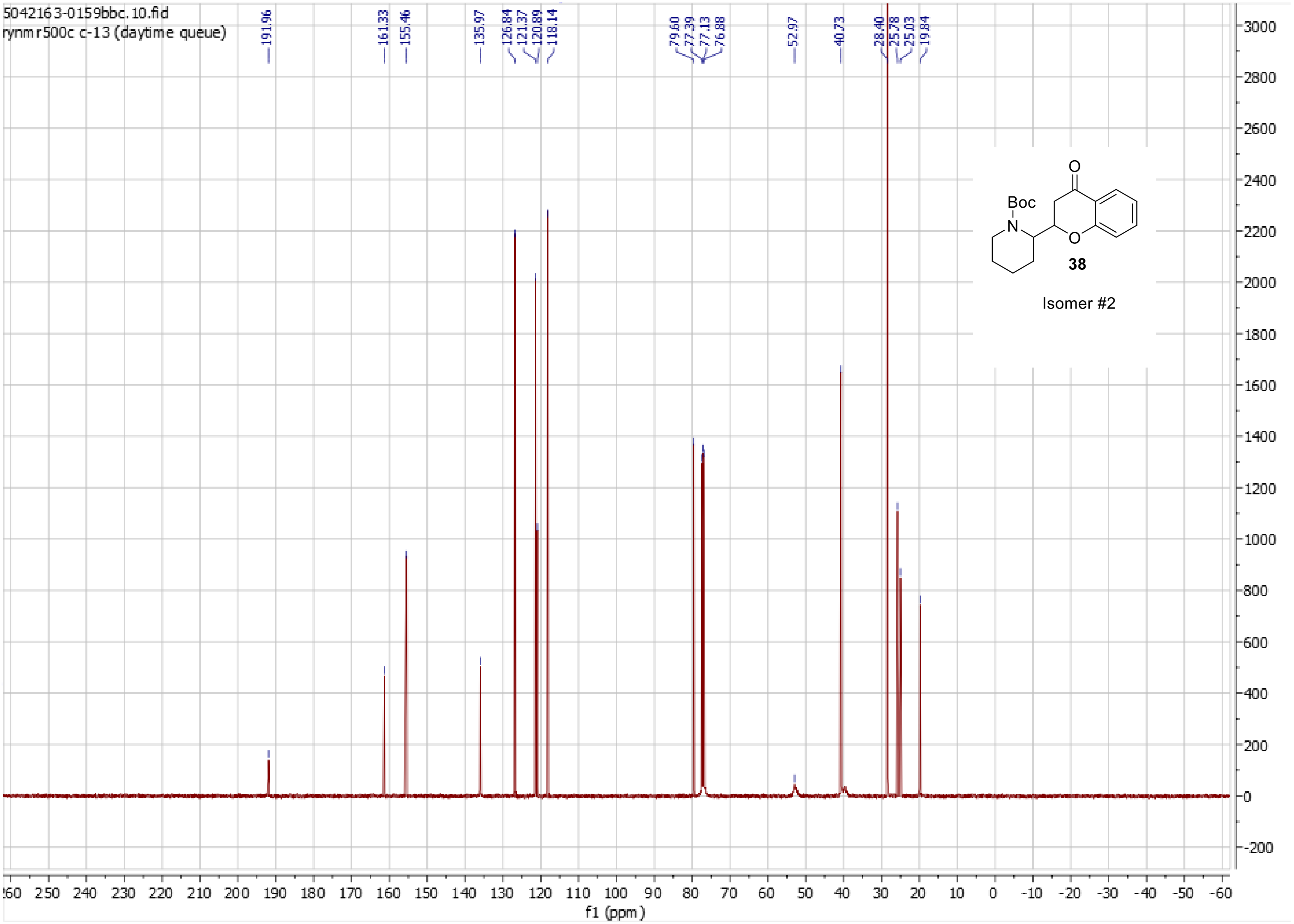
5042163-0159bbc.10.fid
rynmr500c c-13 (daytime queue)
191.96
161.33
155.46
135.97
126.84
121.37
120.89
118.14
79.60
77.39
77.13
76.88
52.97
40.73
28.40
25.78
25.03
19.84
O
Boc
N
O
38
Isomer #2
f1 (ppm)

Characterization of Compound 8:

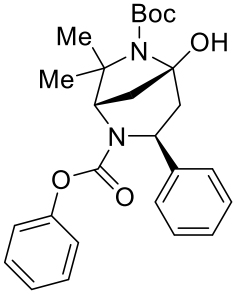

8

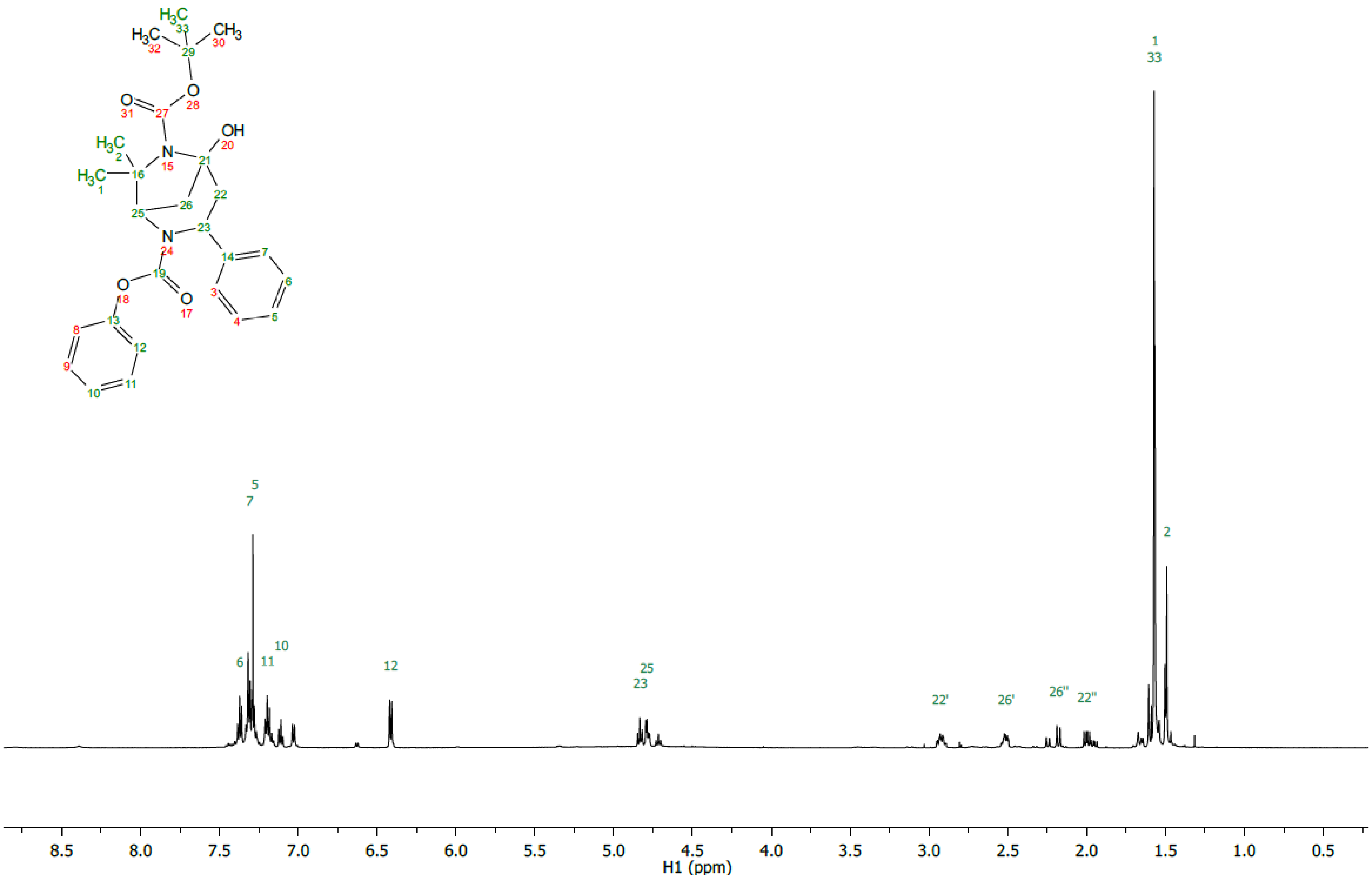

OH
5
7
6
11
10
12
25
23
22'
26'
26''
22''
1
33
2
8.5
8.0
7.5
7.0
6.5
6.0
5.5
5.0
4.5
4.0
3.5
3.0
2.5
2.0
1.5
1.0
0.5
H1 (ppm)

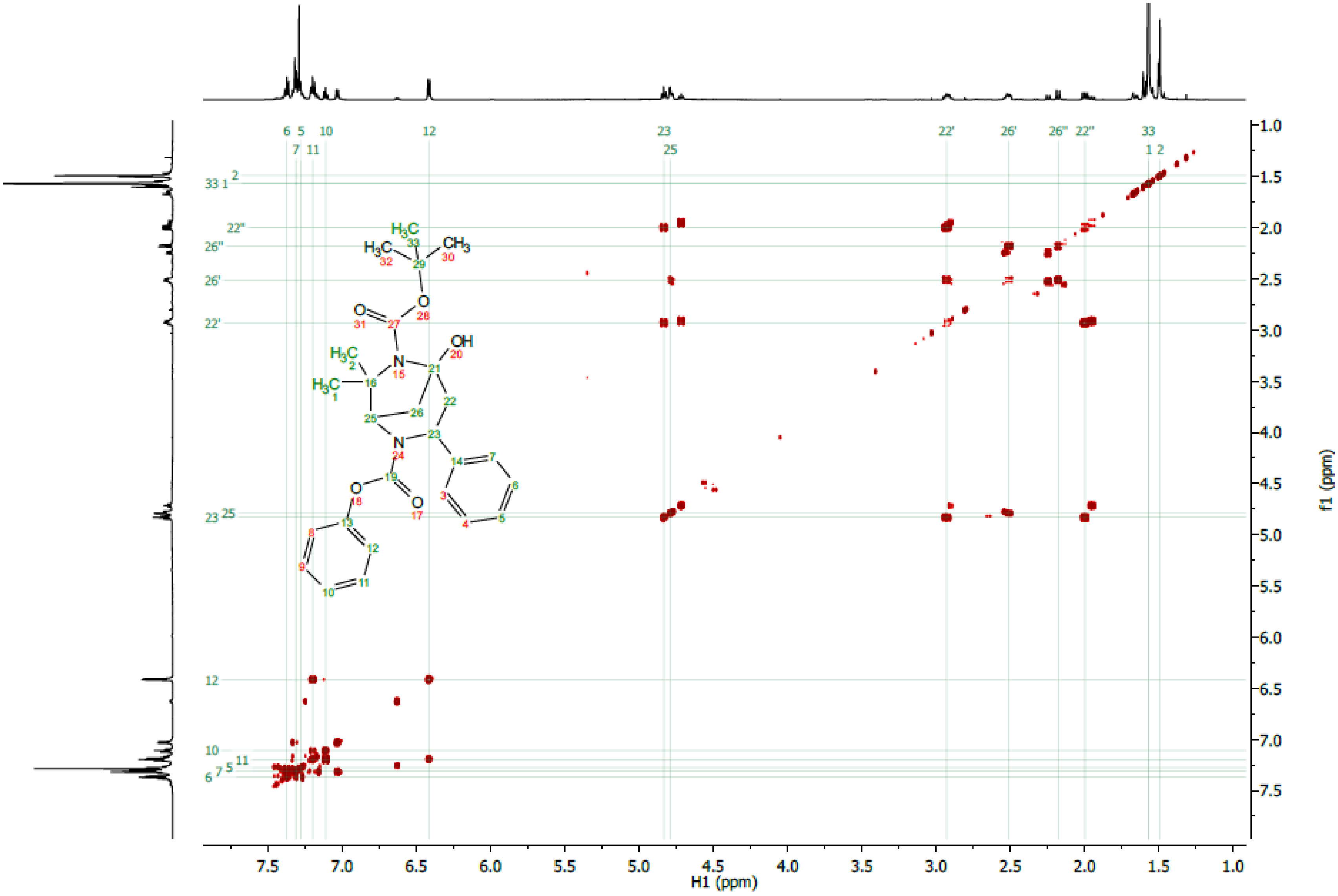

H1 (ppm)
f1 (ppm)

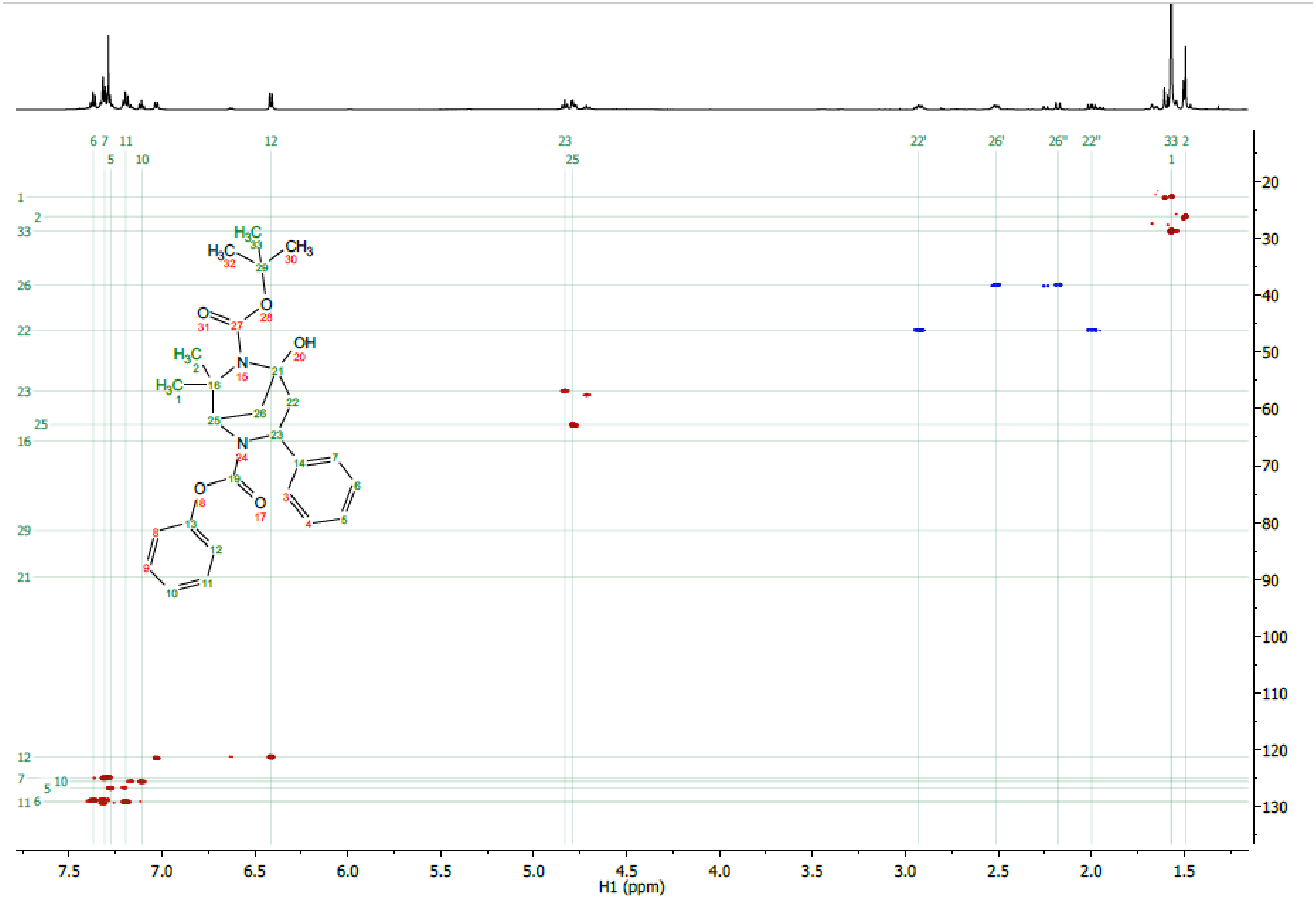

H1 (ppm)
7.5
7.0
6.5
6.0
5.5
5.0
4.5
4.0
3.5
3.0
2.5
2.0
1.5
20
30
40
50
60
70
80
90
100
110
120
130
6 7
5
11
10
12
23
25
22'
26'
26''
22''
33 2
1
OH

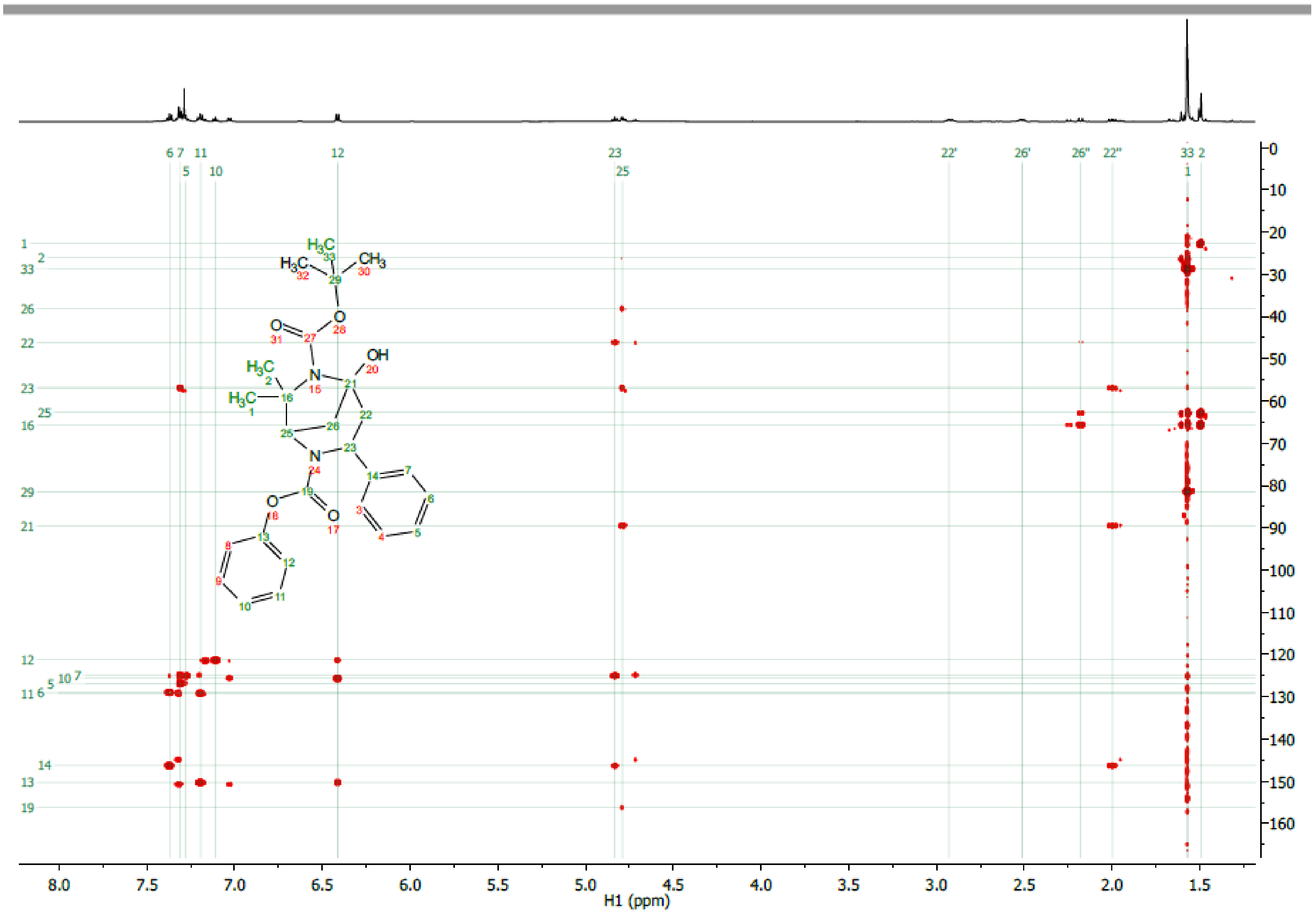
H1 (ppm)
8.0
7.5
7.0
6.5
6.0
5.5
5.0
4.5
4.0
3.5
3.0
2.5
2.0
1.5
OH
N
O
H3C
CH3

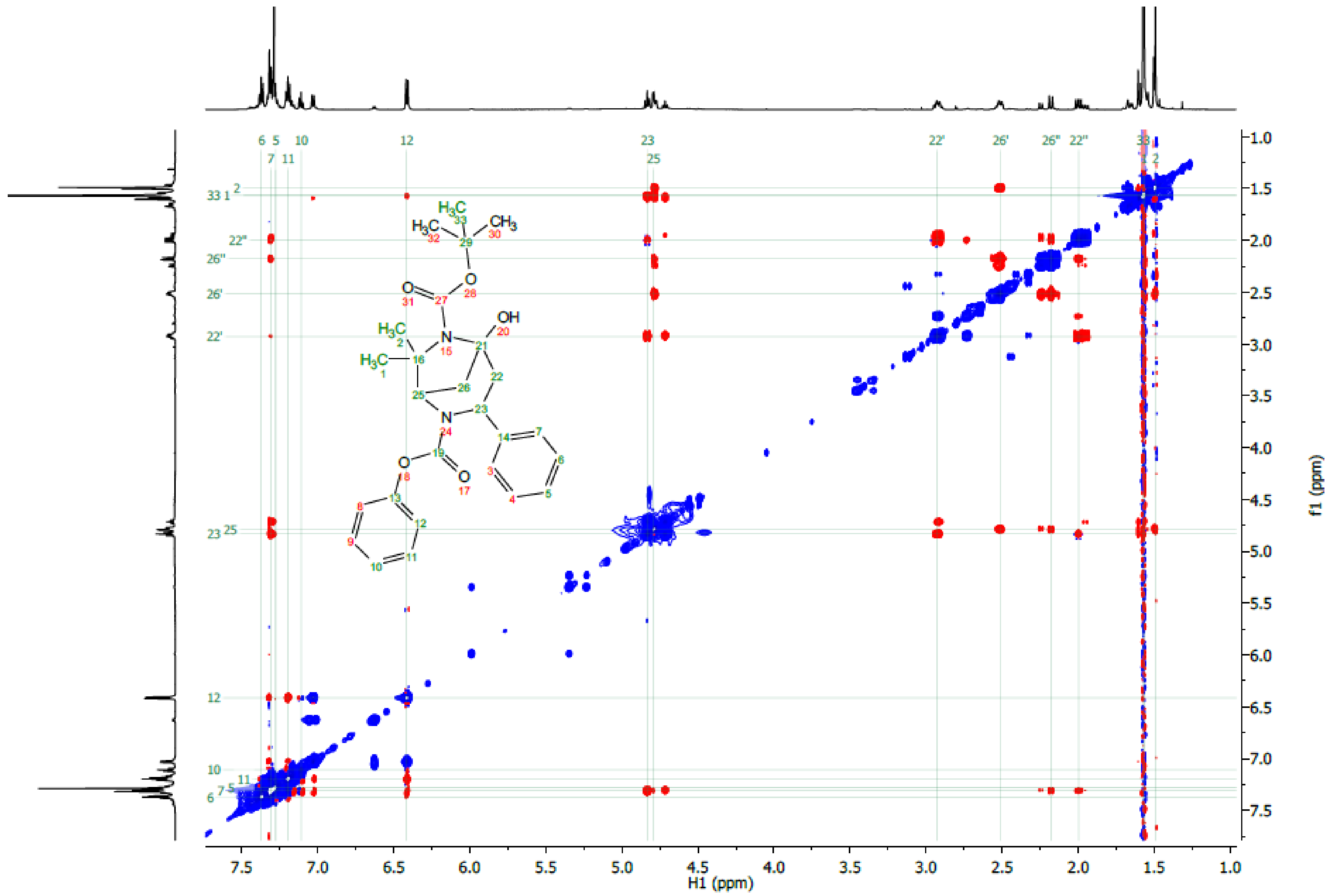

H1 (ppm)
f1 (ppm)
6 5 10 12 23 22' 26' 26'' 22'' 33
7 11 25 2
33 1 2
22''
26''
26'
22'
23 25
12
10
11
7 5
6

Characterization~~eriz~~ of Compound 9 and 10:

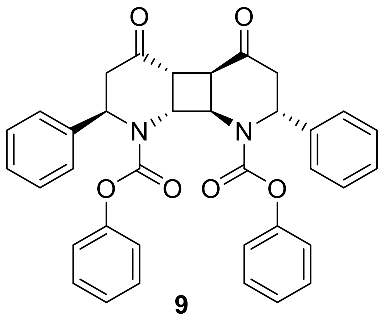

9

2:1

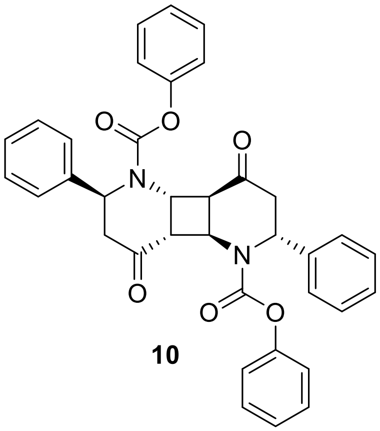

10

0411595-0059-PROTON01 — 0411689-0076 in cd3cn, 25C — Jeff Kuethe —

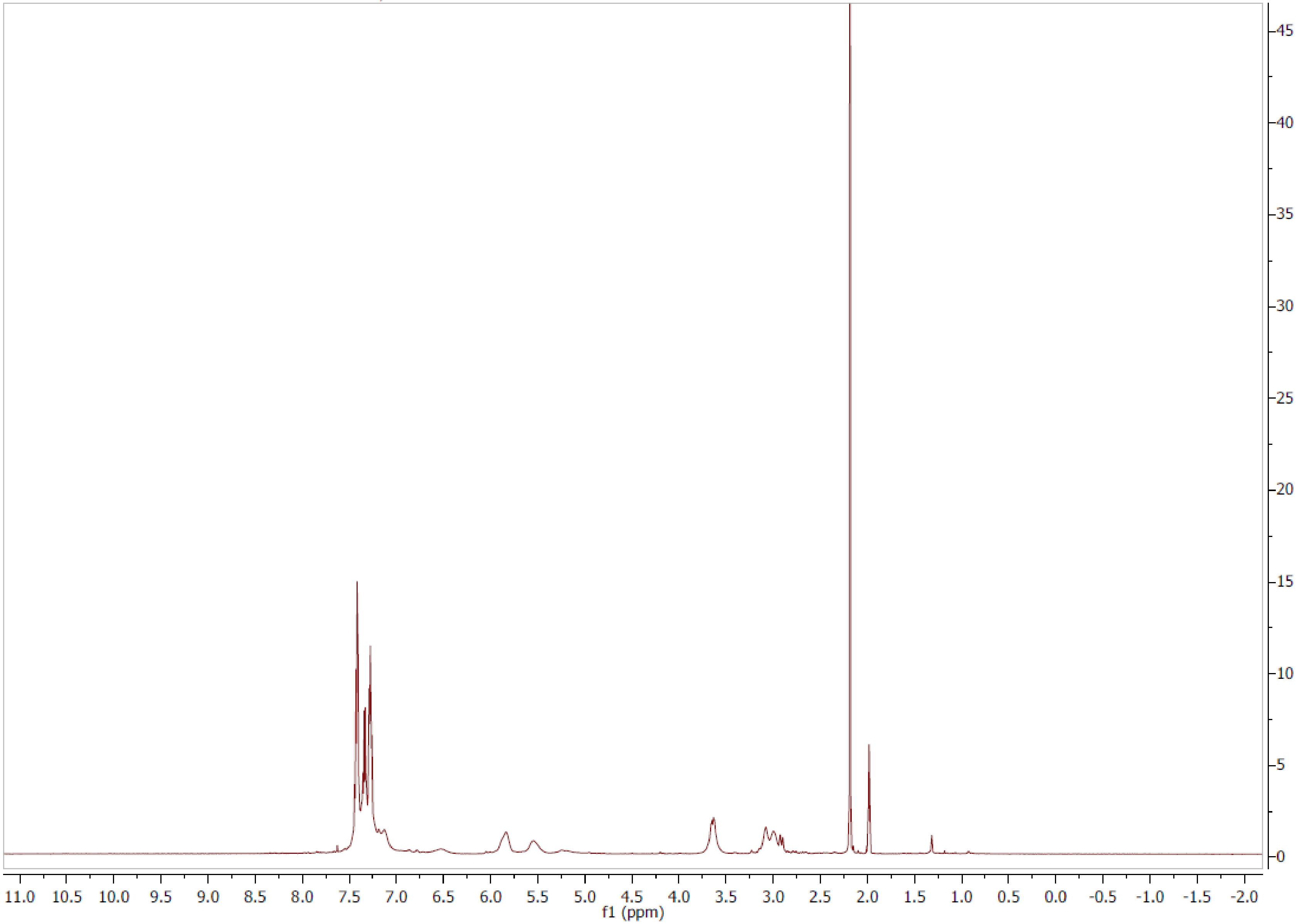

0411595-0059-PROTON04 — 0411689-0076 in cd3cn, -30C — Jeff Kuethe —

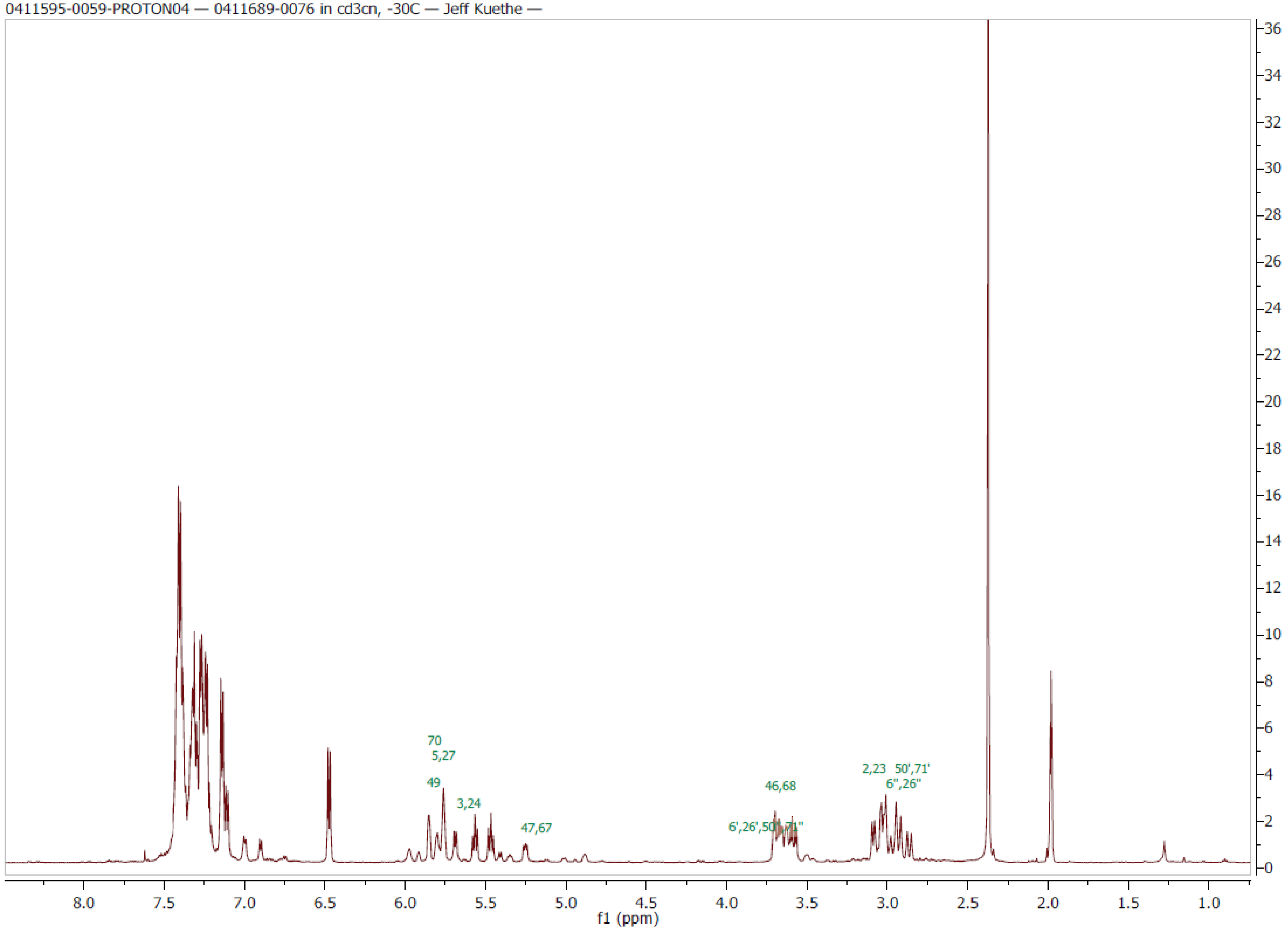

0411595-0059-gCOSY01 — 0411689-0076 in cd3cn, -30C — Jeff Kuethe —

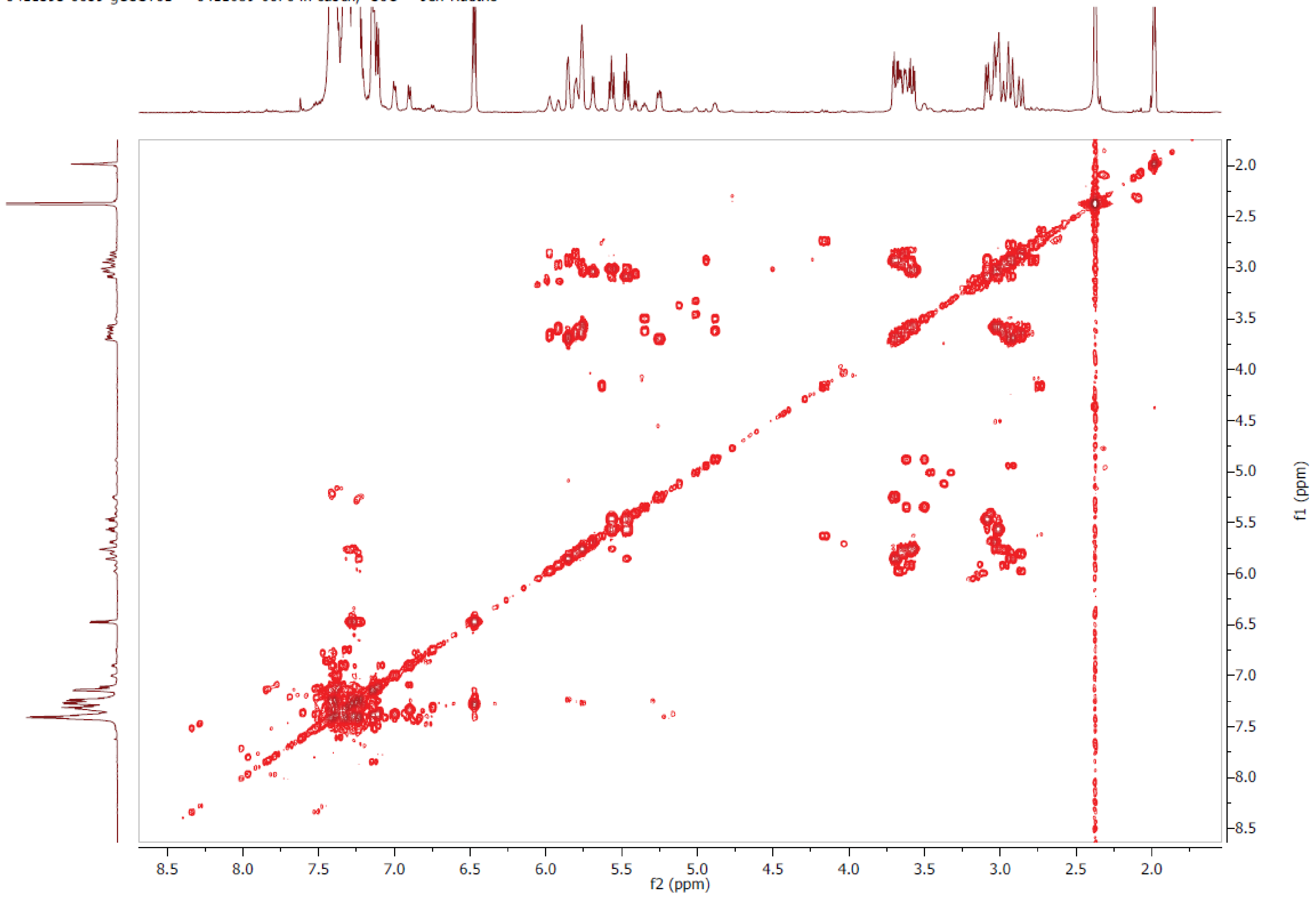

0411595-0059-ROESY01 — 0411689-0076 in cd3cn, -30C — Jeff Kuethe —

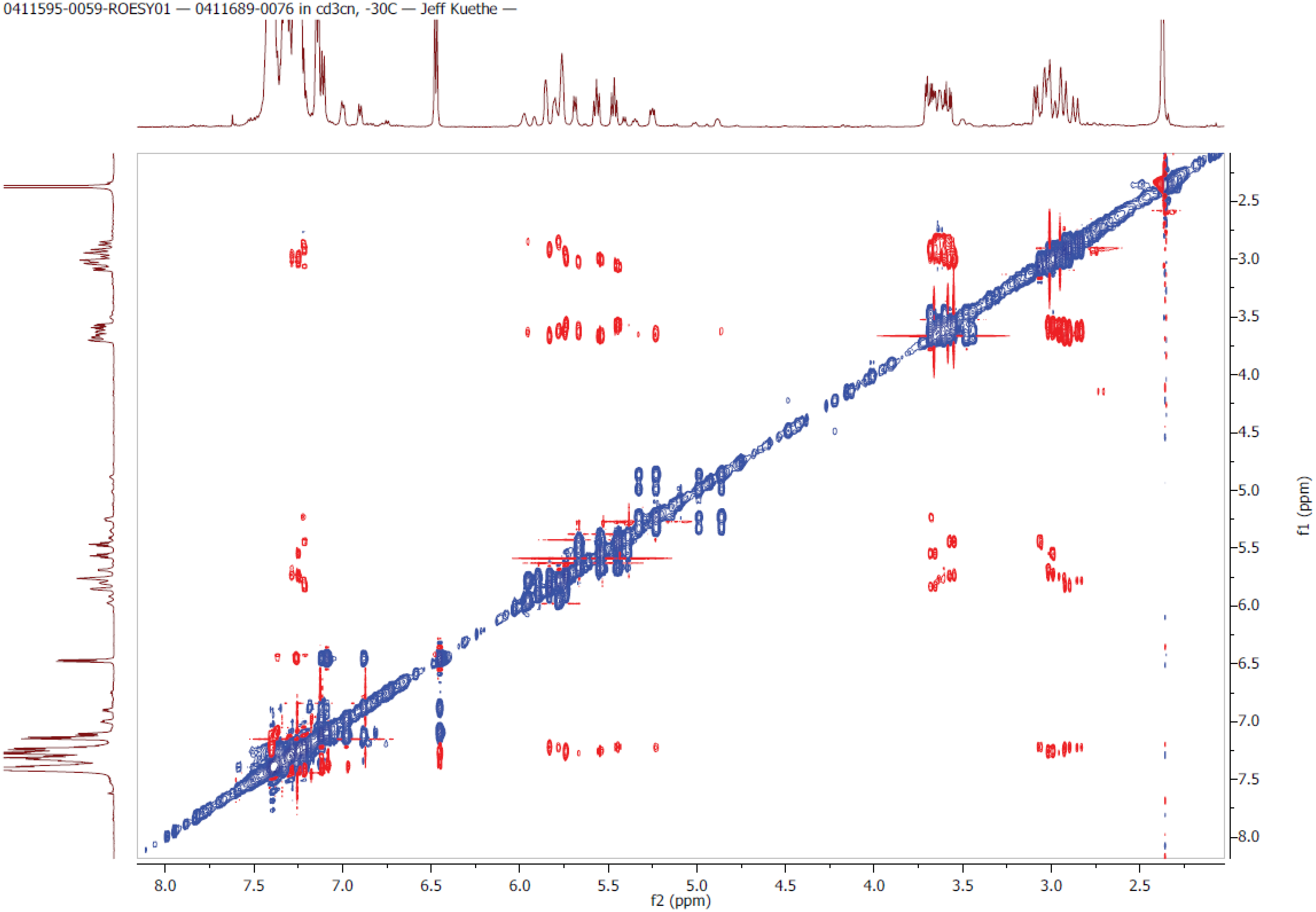

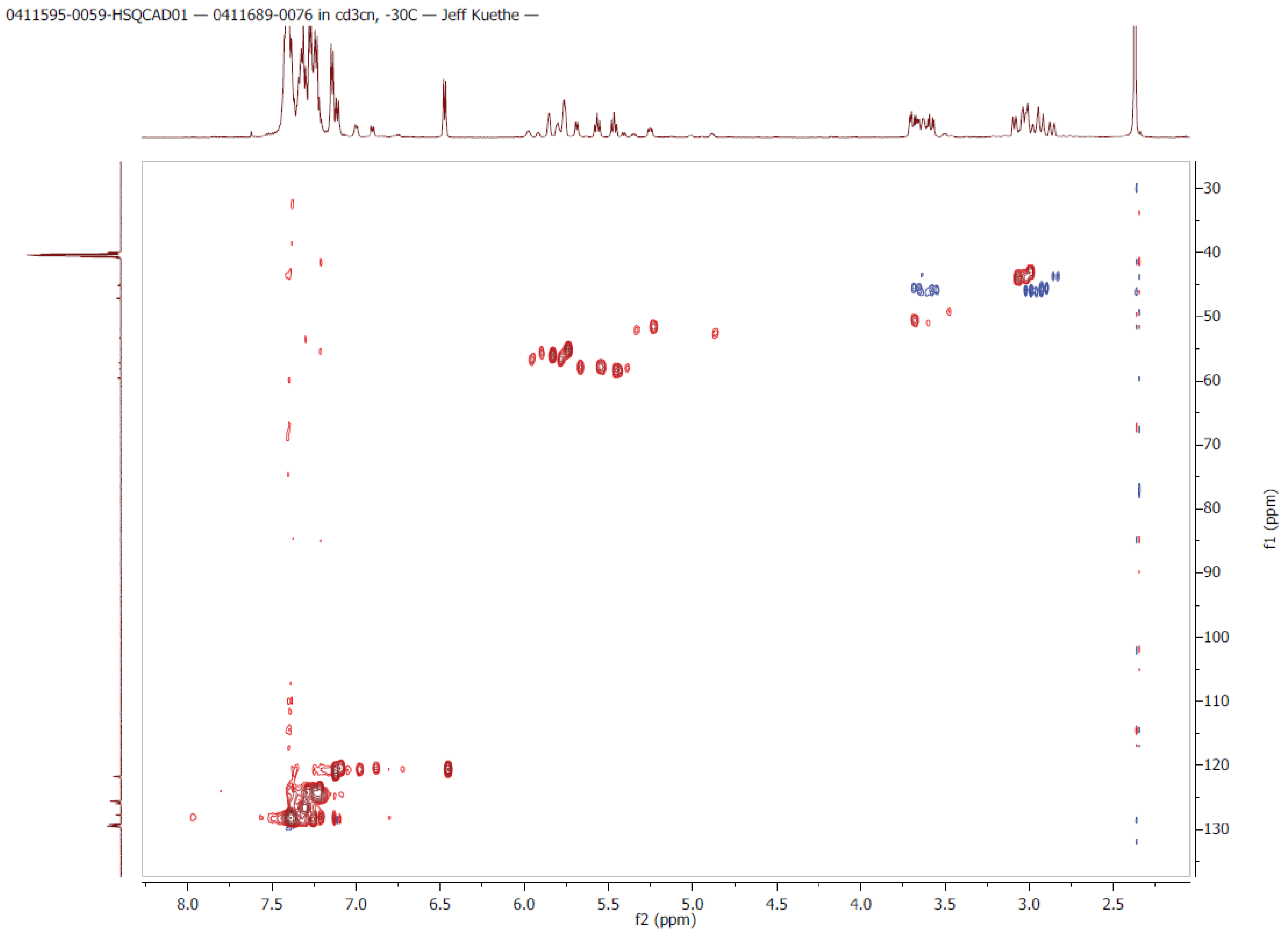
0411595-0059-HSQCAD01 — 0411689-0076 in cd3cn, -30C — Jeff Kuethe —
f2 (ppm)
f1 (ppm)
8.0
7.5
7.0
6.5
6.0
5.5
5.0
4.5
4.0
3.5
3.0
2.5
30
40
50
60
70
80
90
100
110
120
130

0411595-0059b.3.ser — 0411689-0076 in cdcl3, -10C — Jeff Kuethe

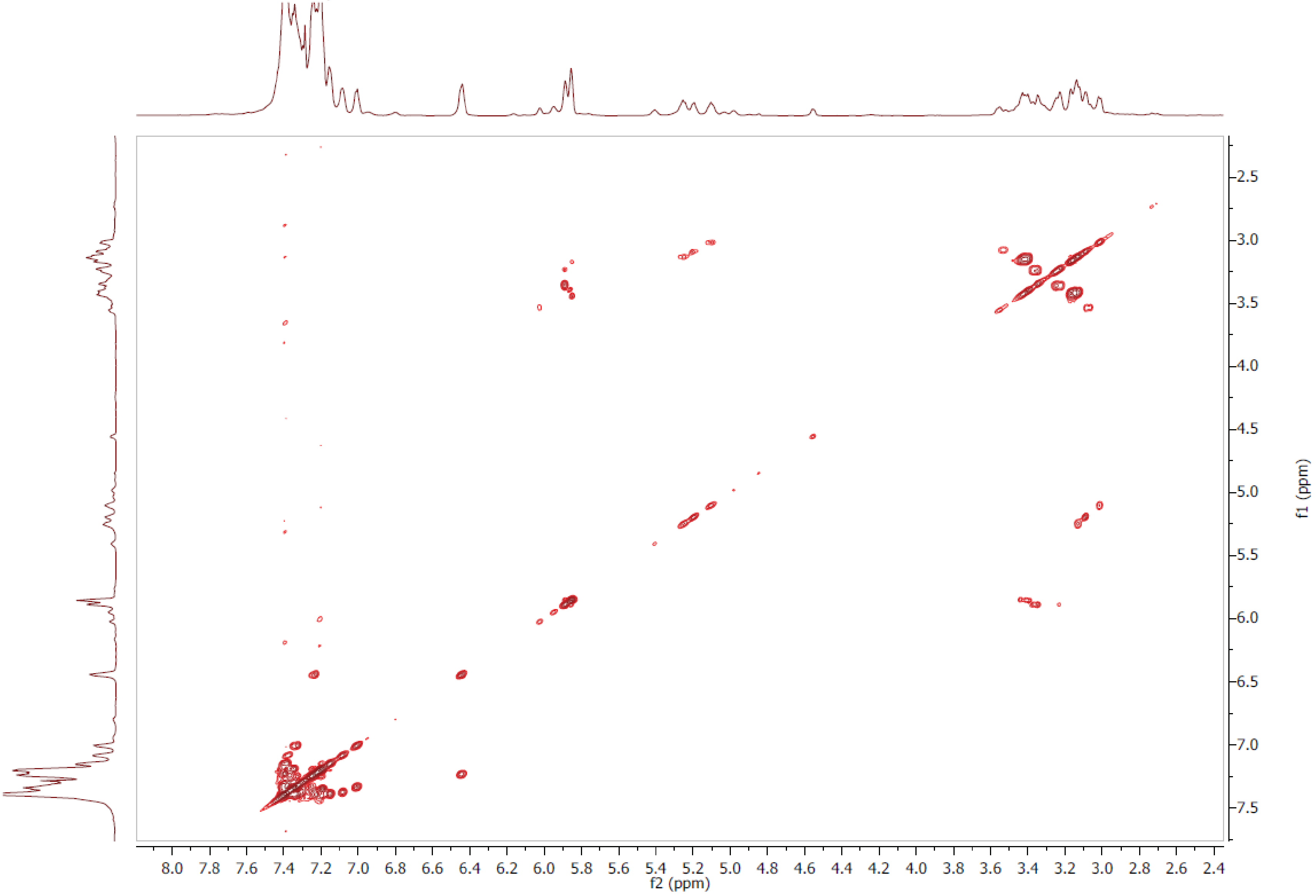

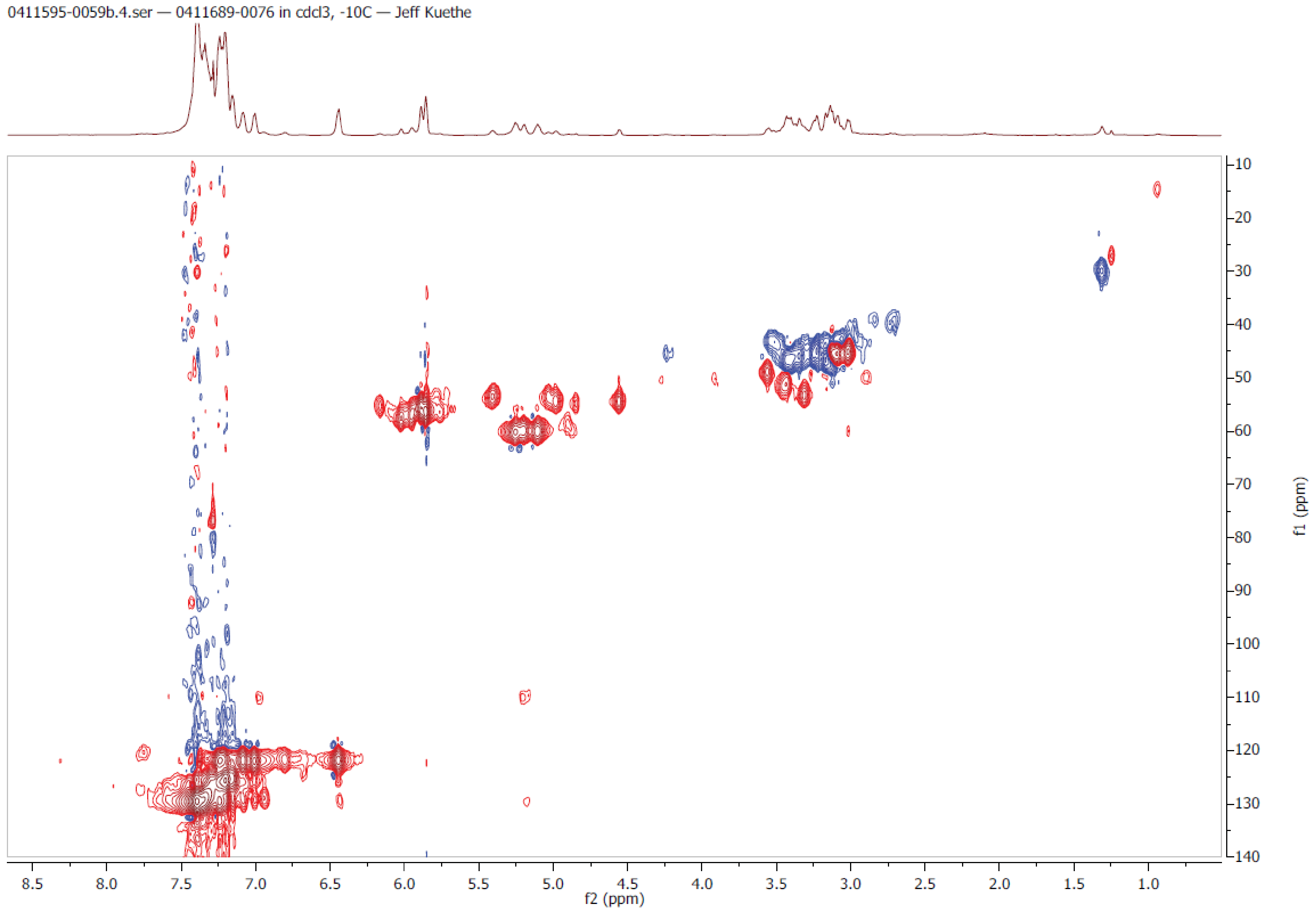
0411595-0059b.4.ser — 0411689-0076 in cdcl3, -10C — Jeff Kuethe
f2 (ppm)
f1 (ppm)
8.5
8.0
7.5
7.0
6.5
6.0
5.5
5.0
4.5
4.0
3.5
3.0
2.5
2.0
1.5
1.0
10
20
30
40
50
60
70
80
90
100
110
120
130
140

0411595-0059b.5.ser — 0411689-0076 in cdcl3, -10C — Jeff Kuethe

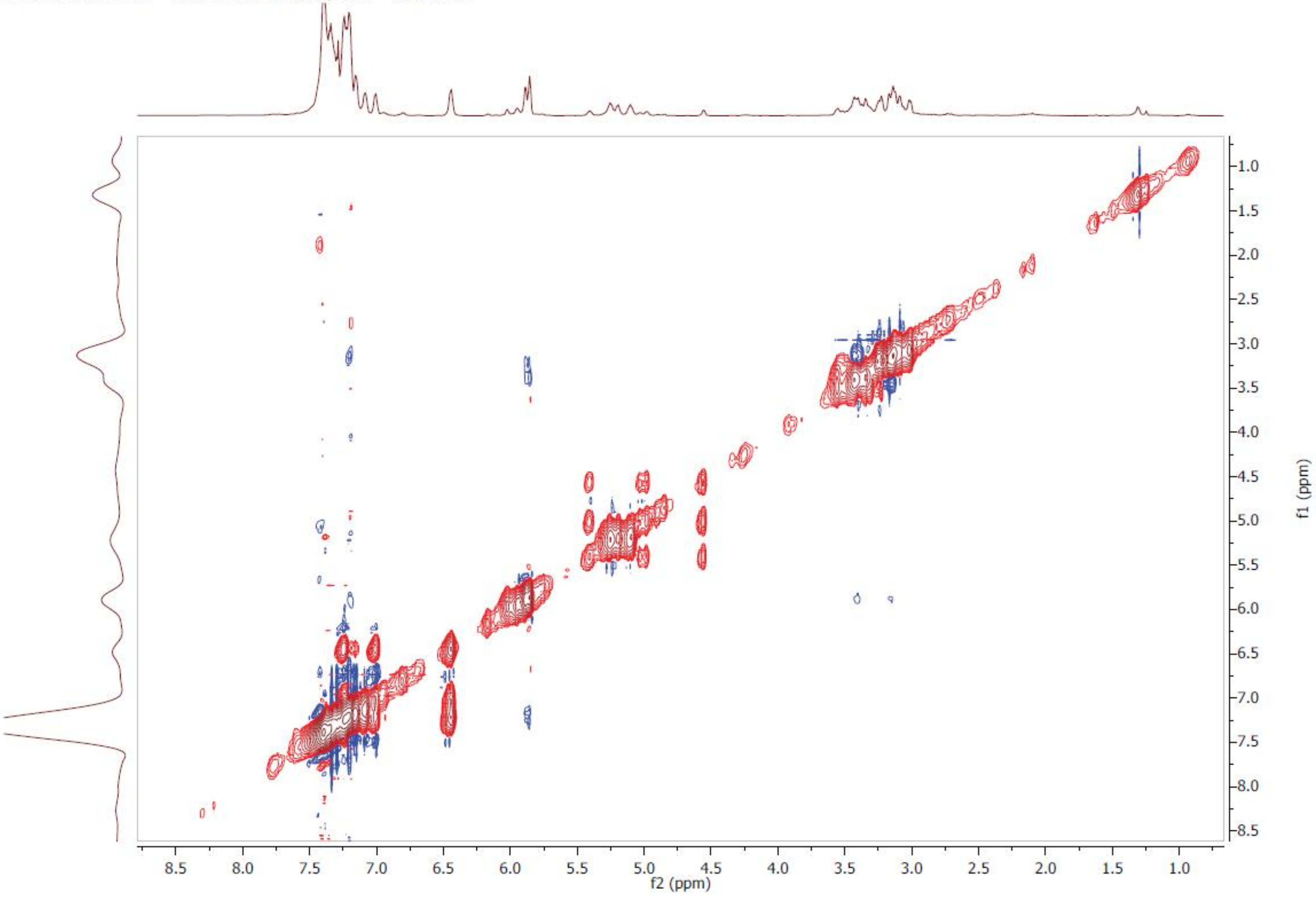

0411595-0059b.10.fid — 0411689-0076 in dmso, 75C on kw600b — Jeff Kuethe

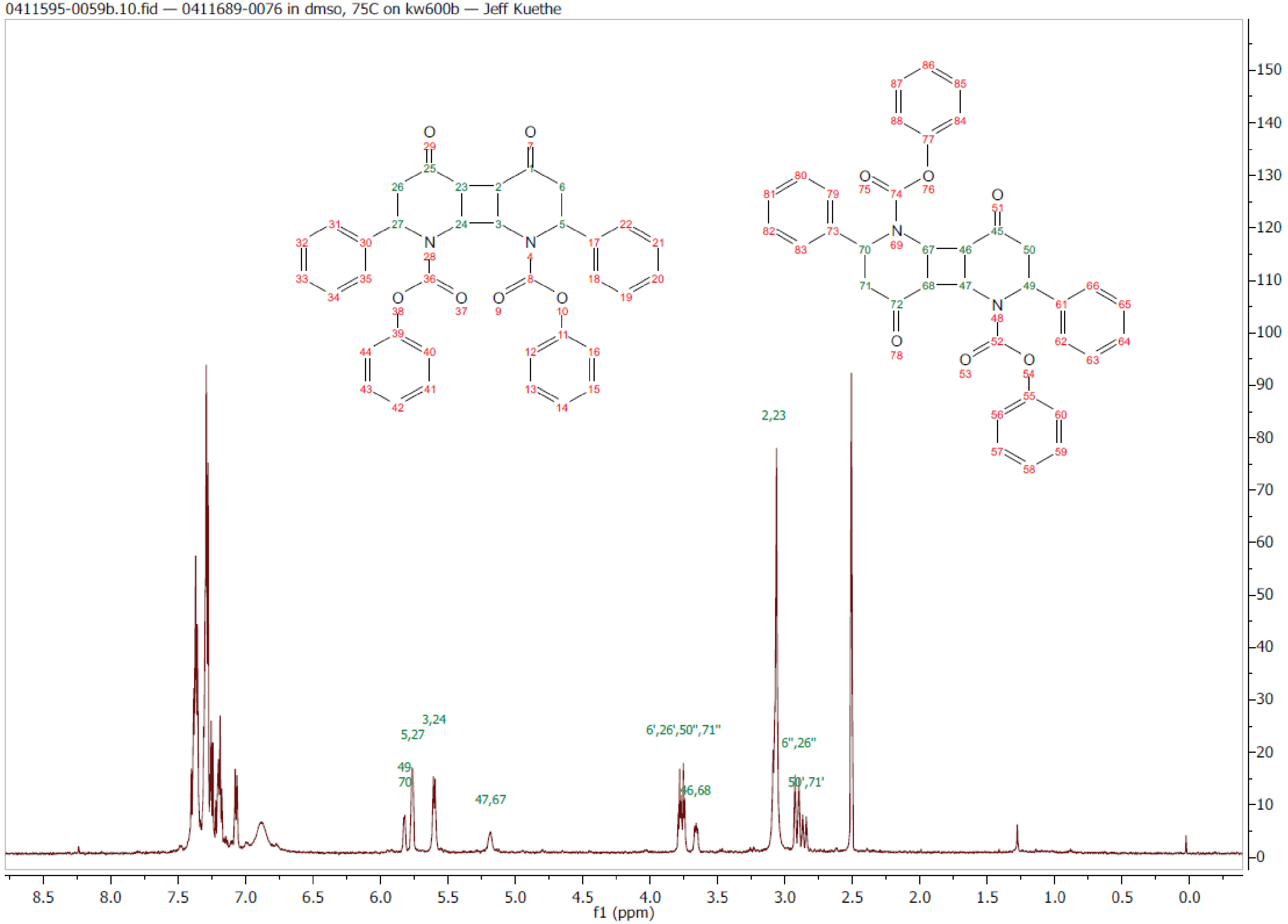

0411595-0059b.11.ser — 0411689-0076 in dmso, 75C on kw600b — Jeff Kuethe

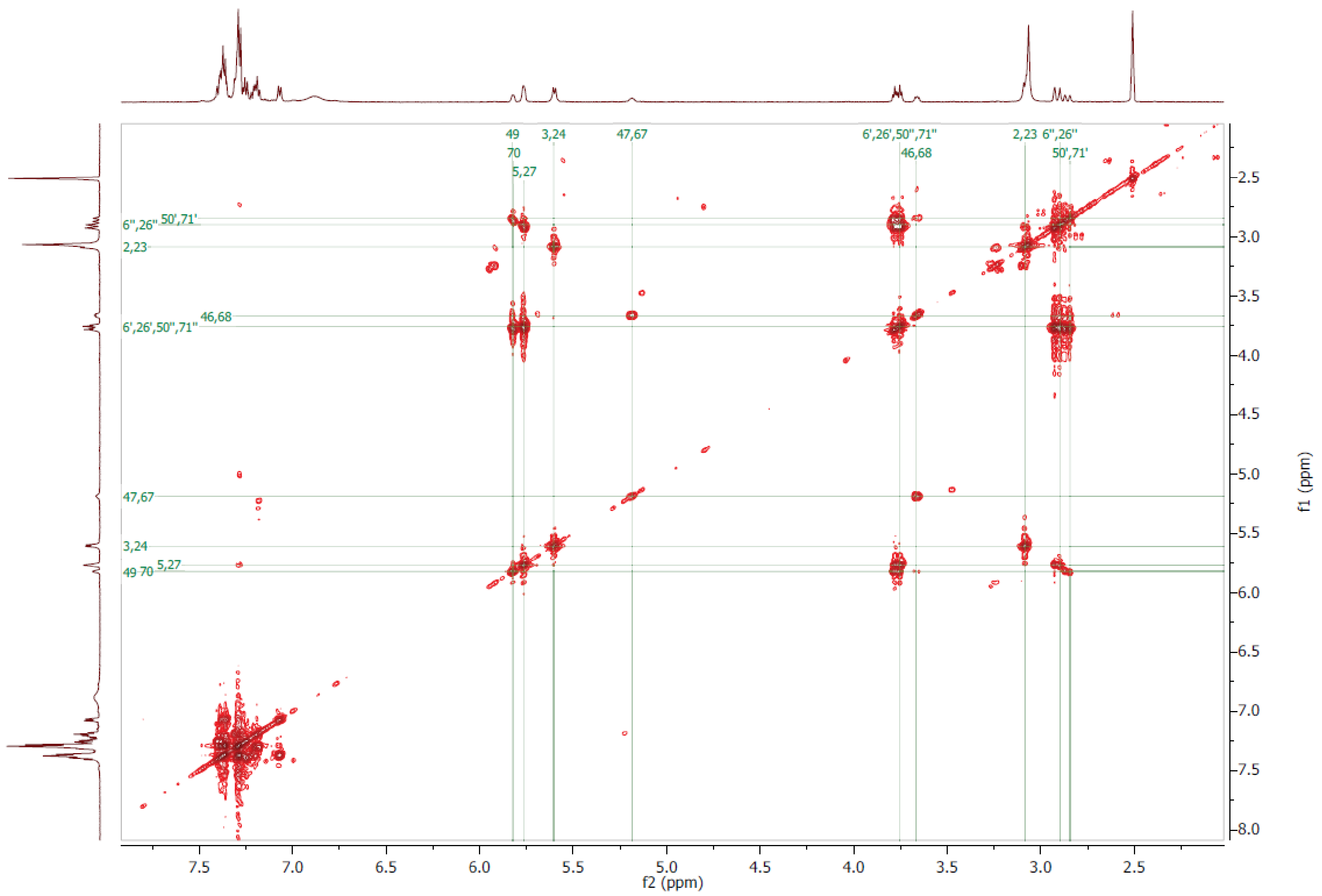

0411595-0059b.12.ser — 0411689-0076 in dmso, 75C on kw600b — Jeff Kuethe

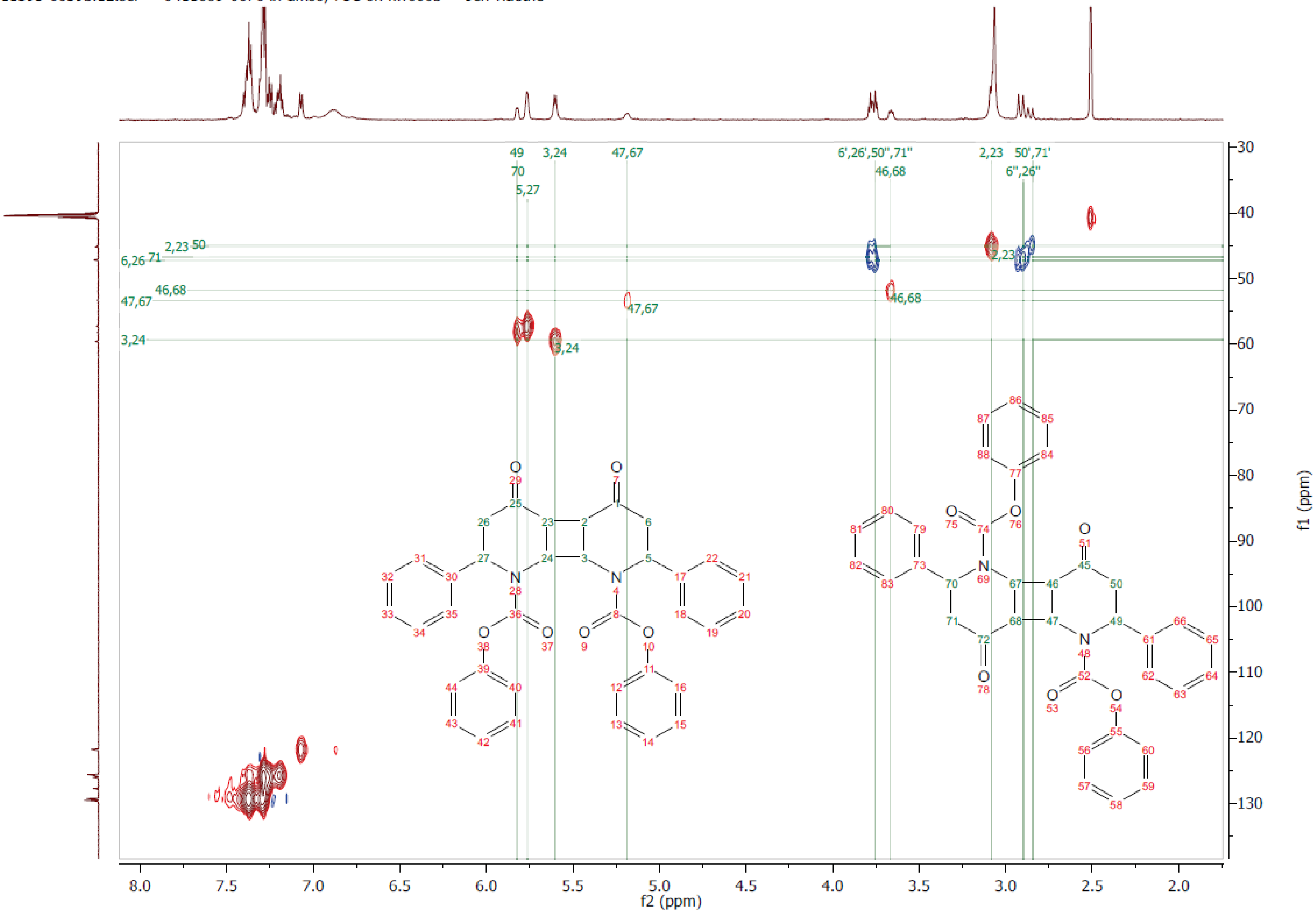

0411595-0059b.13.ser — 0411689-0076 in dmso, 75C on kw600b — Jeff Kuethe

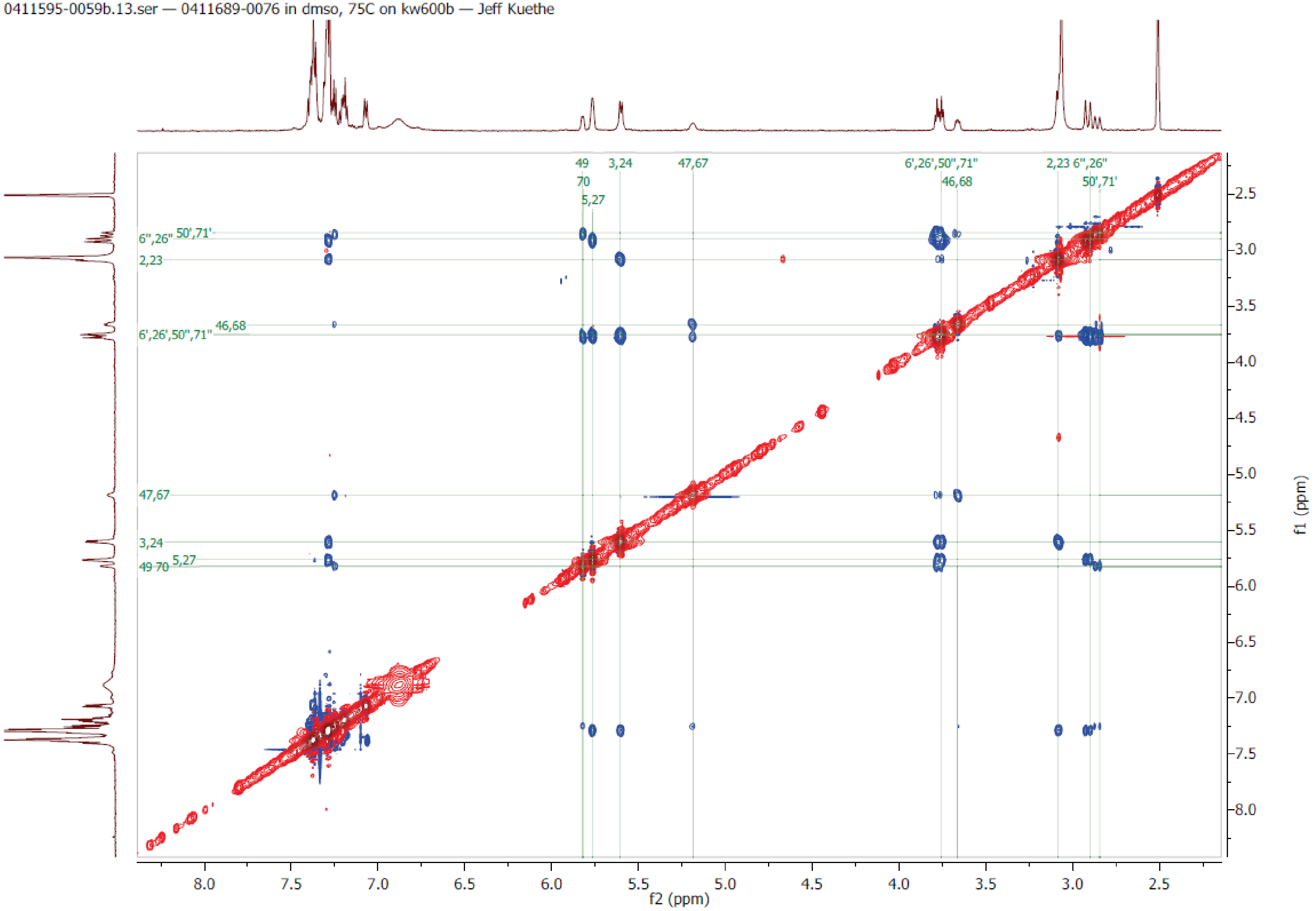

0411595-0059b.14.ser — 0411689-0076 in dmso, 75C on kw600b — Jeff Kuethe

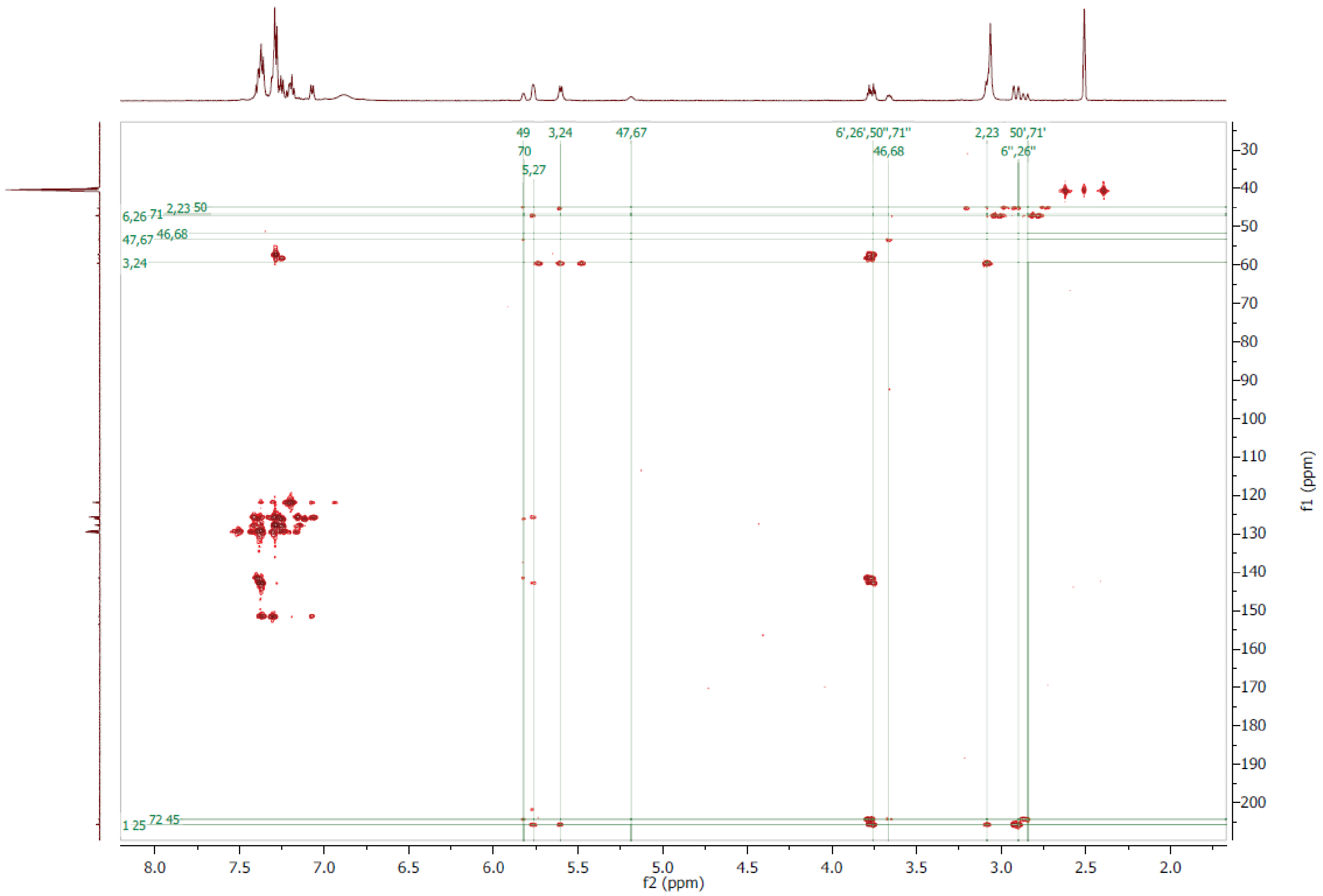

0411595-0059b.14.ser — 0411689-0076 in dmso, 75C on kw600b — Jeff Kuethe

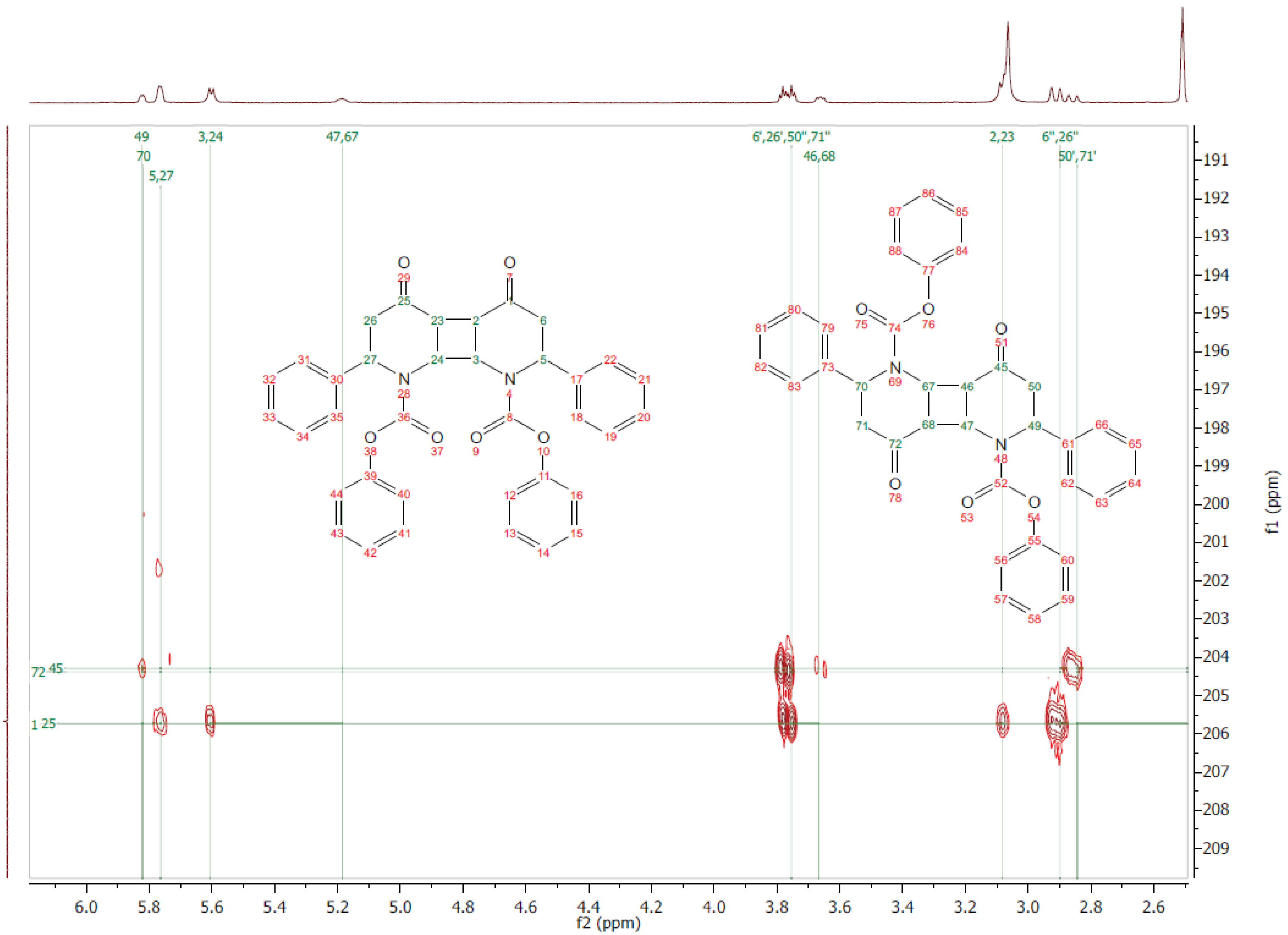

0411595-0059b.15.fid — kw600b c-13 (WALTZ16 decoupled) — 0411689-0076 in dmso, 75C on kw600b — Jeff Kuethe

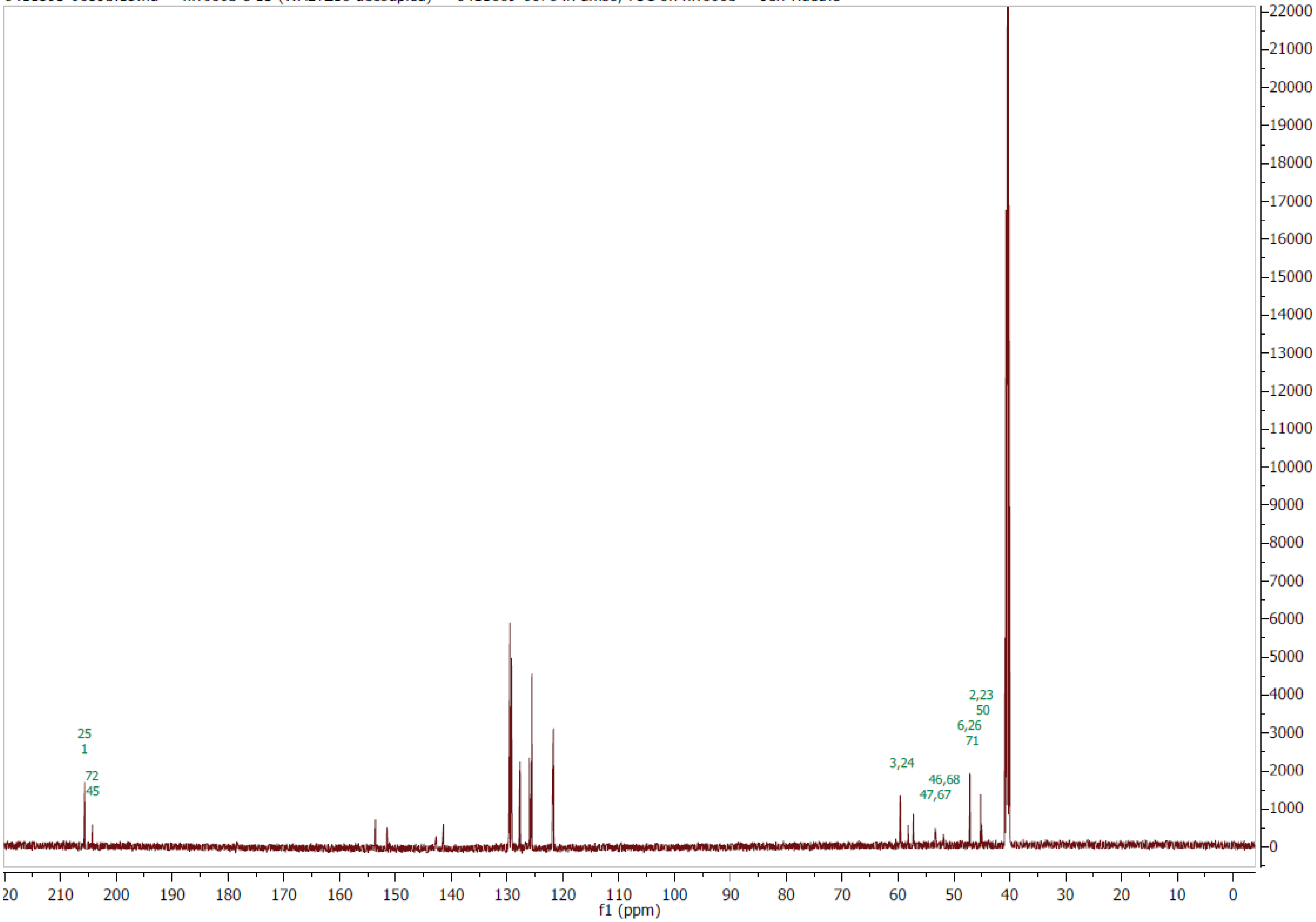

Characterization~~erization~~ of Compound 26a:

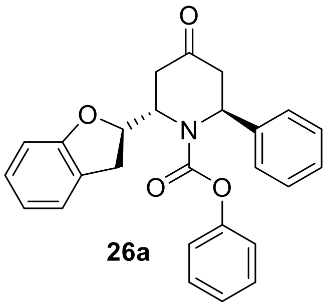

26a

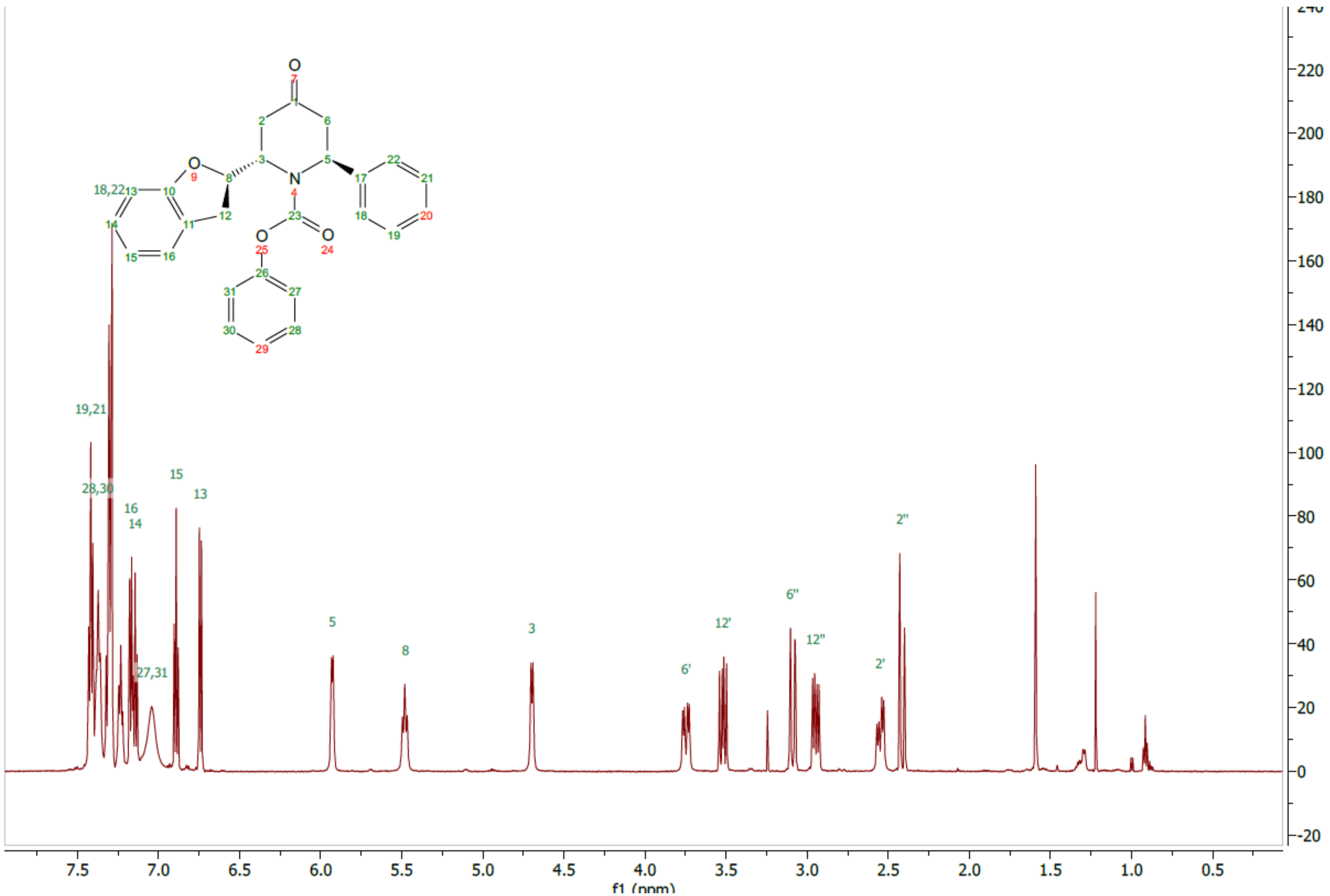
19,21
28,30
16
14
15
13
27,31
18,22
5
8
3
6'
12'
6''
12''
2'
2''
f1 (ppm)

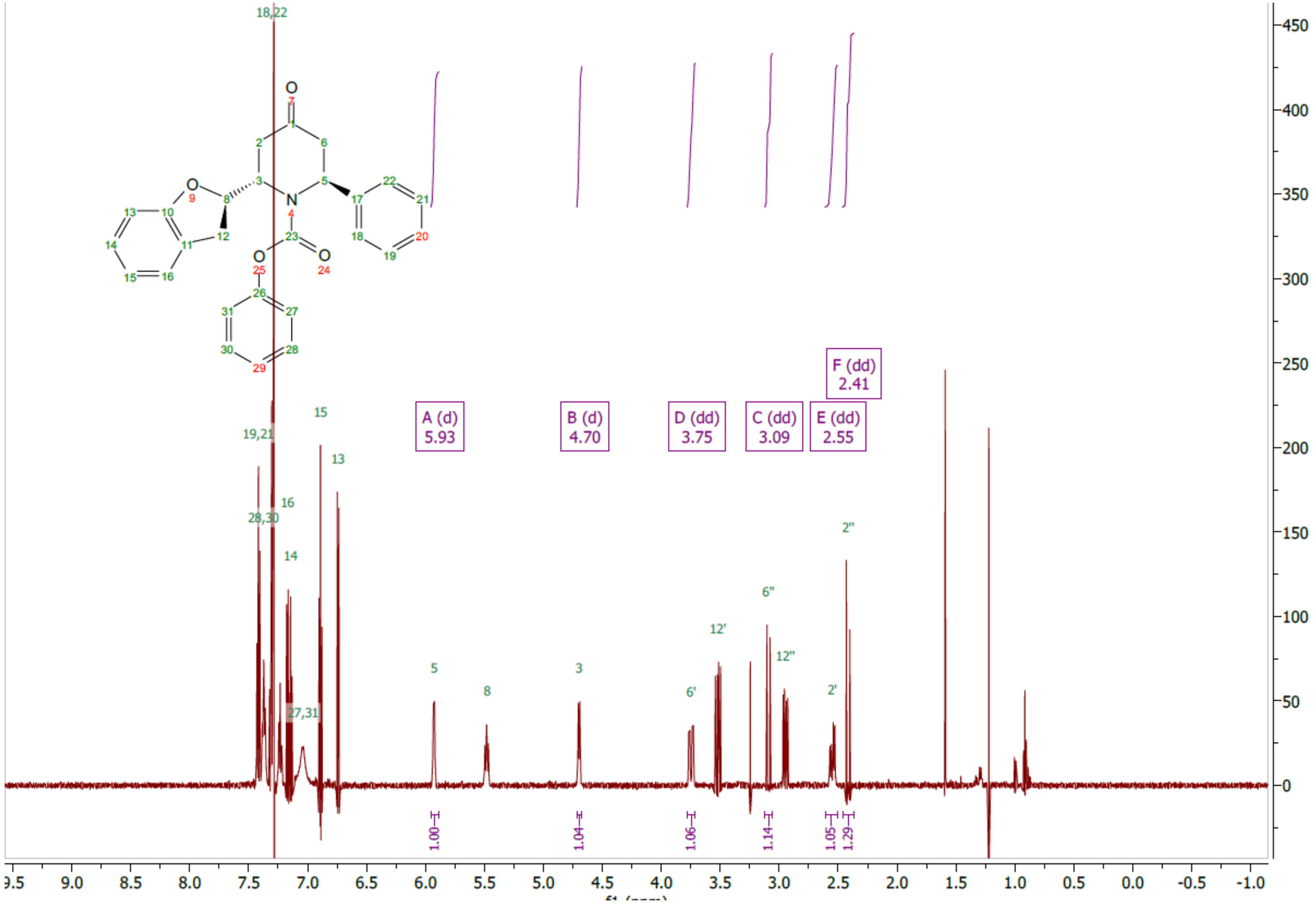

18,22
19,21
28,30
16
14
15
13
27,31
5
8
3
6'
12'
6"
12"
2'
2"
A (d)
5.93
B (d)
4.70
D (dd)
3.75
C (dd)
3.09
E (dd)
2.55
F (dd)
2.41
1.00
1.04
1.06
1.14
1.05
1.29

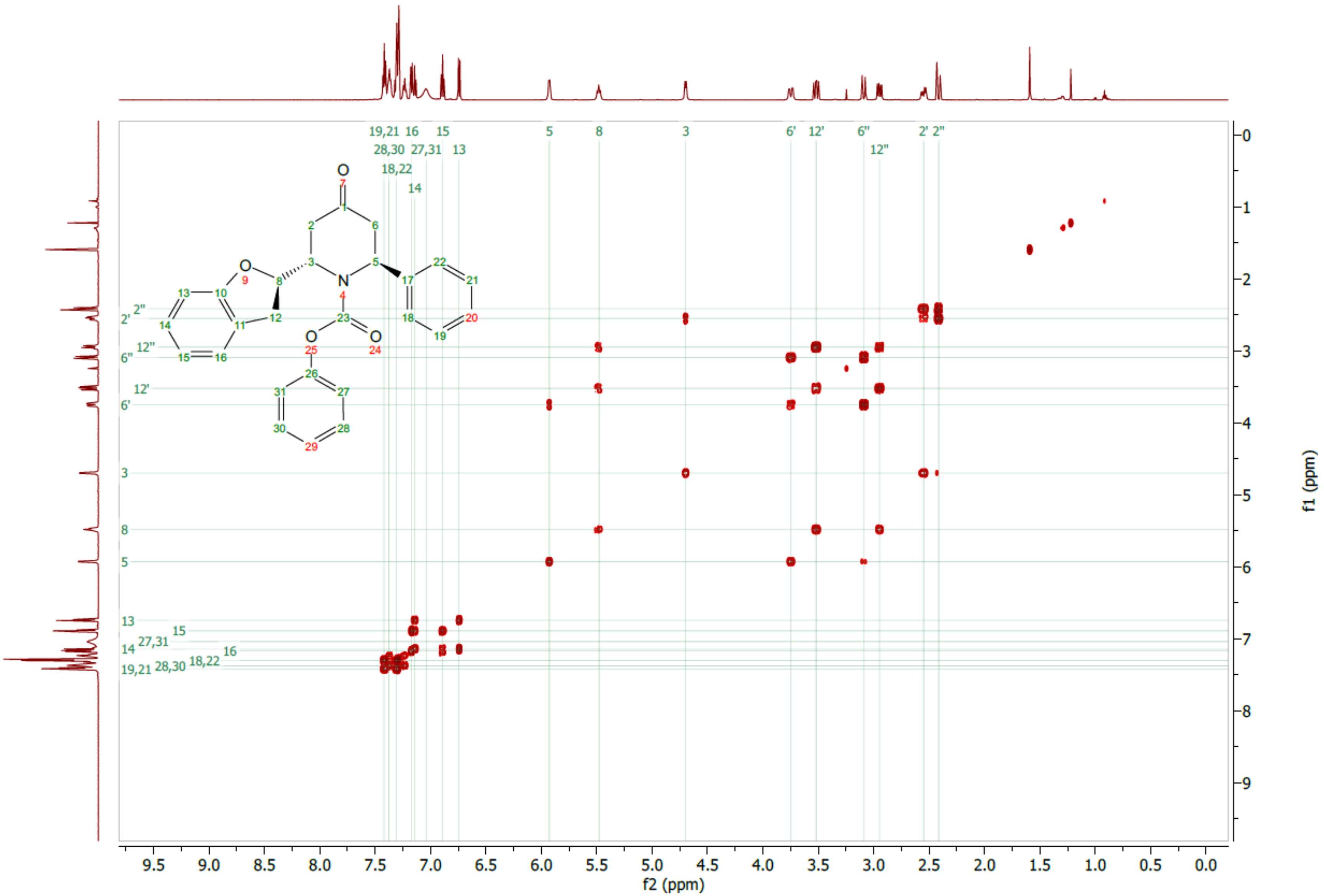

19,21 16 15 5 8 3 6' 12' 6'' 2' 2''
28,30 27,31 13 12''
18,22
14
f2 (ppm)
f1 (ppm)

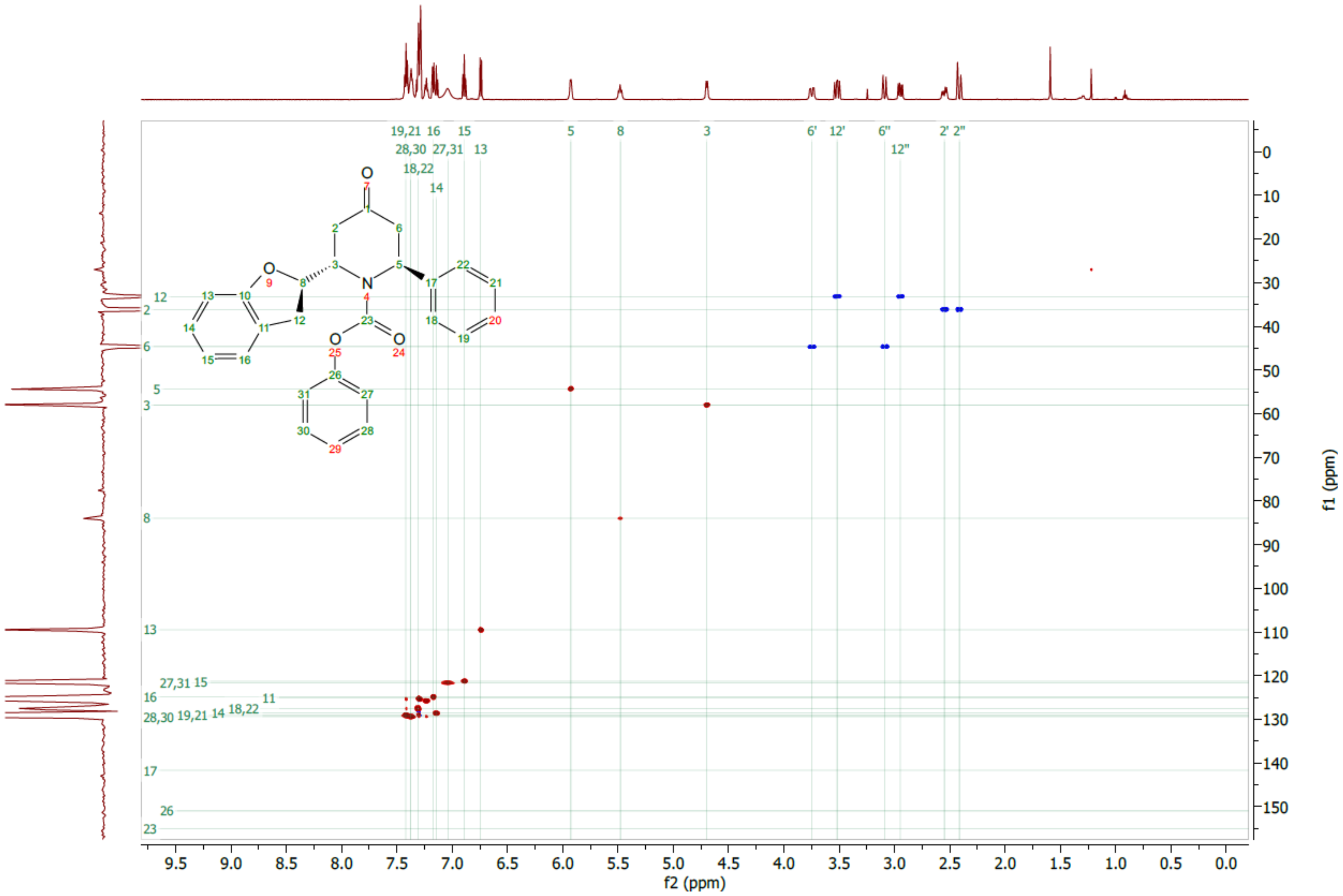

19,21
16
15
28,30
27,31
13
18,22
14
5
8
3
6'
12'
6''
12''
2'
2''
12
2
6
5
3
8
13
27,31 15
16
11
28,30 19,21 14 18,22
17
26
23
f2 (ppm)
f1 (ppm)

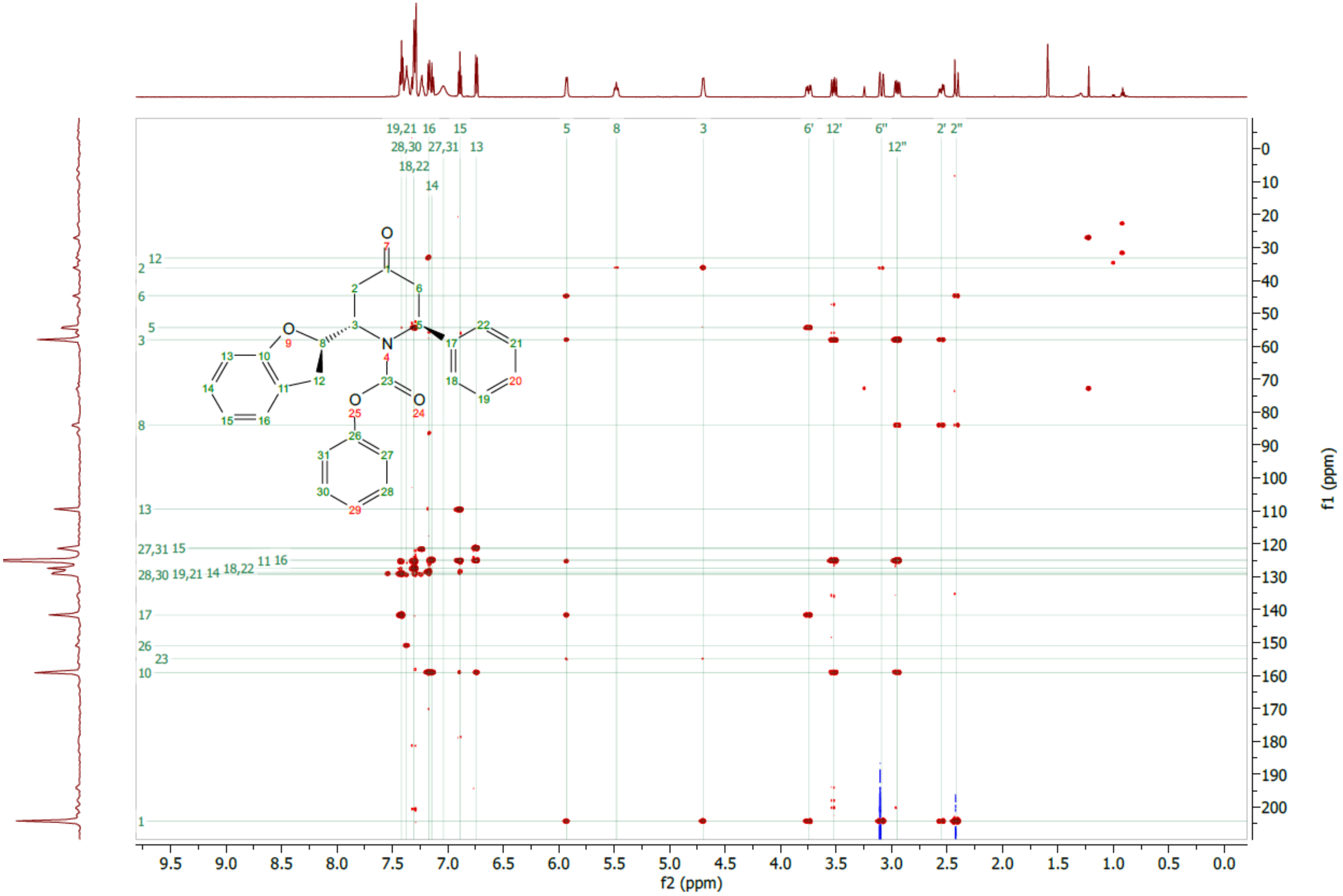

f2 (ppm)
f1 (ppm)

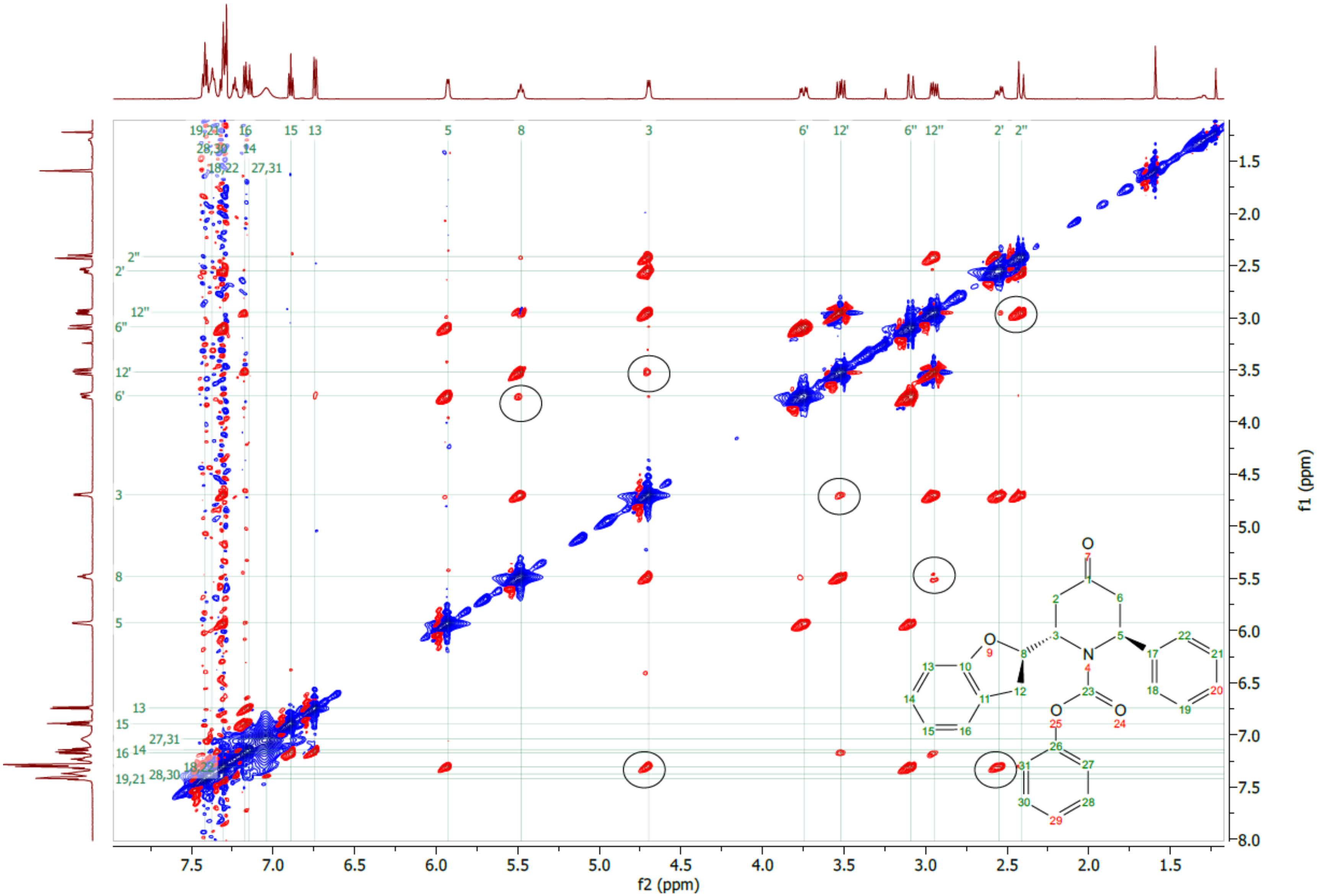

f2 (ppm)
f1 (ppm)
